# Pharmacological sedation strategies for therapeutic gastrointestinal endoscopy: a systematic review and network meta-analysis of randomised controlled trials

**DOI:** 10.3389/fphar.2026.1740424

**Published:** 2026-04-21

**Authors:** Hui Jiang, Yan Xie, Xiaoxue Qin, Qian Liu, Yaodan Zhang, Chunlin Ge, Ilyar Mamtili

**Affiliations:** Department of Anesthesiology, Shanghai Xuhui Central Hospital, Shanghai, China

**Keywords:** analgesia, cardiorespiratory safety, endoscopic retrograde cholangiopancreatography, endoscopic submucosal dissection, network meta-analysis, procedural sedation, systematic review

## Abstract

**Background:**

Therapeutic gastrointestinal endoscopy, including endoscopic retrograde cholangiopancreatography and endoscopic submucosal dissection, requires effective sedation strategies to ensure procedural success and patient safety. However, optimal pharmacological regimens remain unclear, particularly for prolonged procedures.

**Methods:**

We searched PubMed, Embase, and the Cochrane Central Register of Controlled Trials (CENTRAL) for randomised controlled trials comparing pharmacological sedation strategies in patients undergoing therapeutic gastrointestinal endoscopy. Outcomes included procedural interference events, hypoxia, hypotension, bradycardia, recovery time, induction time, satisfaction, and postoperative nausea and vomiting. Risk of bias was assessed using the Cochrane RoB 2.0 tool, and certainty of evidence was rated using GRADE framework. A frequentist random-effects network meta-analysis was conducted, along with cluster ranking of co-primary outcomes to evaluate benefit-risk trade-offs.

**Results:**

Sixty randomised controlled trials involving 7,071 patients and 32 pharmacological regimens were included. Compared with propofol-opioid, which remained the reference standard, no regimen significantly reduced procedural interference events. Ketamine-propofol demonstrated consistent advantages across hypoxia (relative risk [RR] 0.12, 95% confidence interval [CI] 0.03 to 0.59, P = 0.009; moderate certainty), hypotension (RR 0.28, 95% CI 0.09 to 0.83, P = 0.021; low certainty), and bradycardia (RR 0.11, 95% CI 0.01 to 0.86, P = 0.035; moderate certainty). Cluster rank analyses identified ketamine-propofol and lidocaine-midazolam-propofol as the highest-ranking regimens in both efficacy and safety domains. Meta-regression revealed no significant effect modifiers.

**Conclusion:**

While propofol-opioid remains the standard reference, alternative sedation strategies such as ketamine-propofol and lidocaine-midazolam-propofol offer favourable profiles for therapeutic gastrointestinal endoscopy. These findings support individualised regimen selection based on patient and procedural needs.

**Systematic Review Registration:**

https://www.crd.york.ac.uk/PROSPERO/, identifier CRD420251018215.

## 1 Introduction

Gastrointestinal endoscopic procedures, such as endoscopic retrograde cholangiopancreatography (ERCP) and endoscopic submucosal dissection (ESD), are indispensable tools in the diagnosis and treatment of digestive diseases (Crinò et al., 2025; Libânio et al., 2023). These therapeutic interventions are technically demanding and often prolonged, necessitating sedation strategies that ensure patient comfort, procedural efficiency, and physiological stability (Bruni et al., 2025; Melita et al., 2024). Selecting an appropriate sedation regimen remains a challenge, requiring a balance between cardiorespiratory safety, adequate sedation depth, and prompt recovery.

A wide range of pharmacological agents have been employed for endoscopic sedation, either as monotherapies or in combination regimens. Propofol remains the most widely used sedative due to its rapid onset and short duration, however, its use–particularly when combined with opioids–is limited by dose-dependent respiratory depression and hypotension (Sidhu et al., 2024). Despite these risks, propofol-opioid regimens remain the clinical standard in many settings (Sneyd et al., 2022). A 2018 systematic review of sedation for ERCP suggested that dexmedetomidine-based combinations showed procedural and safety benefits (Li et al., 2018). However, newer agents such as ciprofol and remimazolam, alongside tailored combination regimens, have since reshaped clinical practice (Zhou et al., 2025; Sneyd et al., 2021; Hara et al., 2023).

Although the 2023 Consensus guidelines for the perioperative management of patients undergoing ERCP provided valuable multidisciplinary recommendations on sedation and anaesthetic strategies, their recommendations were based on expert opinion rather than direct comparative evidence between pharmacological regimens (Azimaraghi et al., 2023). As highlighted in the accompanying editorial, these guidelines, while methodologically rigorous, were developed in the context of limited trial data and thus relied heavily on consensus via Delphi methods rather than quantitative synthesis (Howell and Absalom, 2023). Consequently, they do not address the relative efficacy or safety of specific sedation regimens, underscoring the need for formal comparative effectiveness research to support sedation strategy selection in complex therapeutic endoscopy.

To address this evidence gap, we conducted a systematic review and network meta-analysis of randomised controlled trials (RCTs) comparing the efficacy and safety of pharmacological sedation strategies in patients undergoing therapeutic gastrointestinal endoscopy, including ERCP and ESD. The population comprised patients undergoing therapeutic gastrointestinal endoscopy, the interventions and comparators were different pharmacological sedation regimens, and the outcomes included procedural interference events, cardiorespiratory adverse events (hypoxia, hypotension, bradycardia), recovery time, and satisfaction reported by patients and endoscopists. We further assessed the certainty of evidence using the GRADE approach and explored heterogeneity through meta-regression. These findings provide comparative evidence to inform sedation strategy selection and optimise clinical practice.

## 2 Methods

This network meta-analysis was conducted in accordance with the Preferred Reporting Items for Systematic Reviews and Meta-Analyses (PRISMA) Extension Statement for Network Meta-Analyses (Page et al., 2021). The study protocol was prospectively registered in the PROSPERO database (registration number CRD420251018215) on 24 March 2025.

### 2.1 Eligibility criteria

We included RCTs evaluating intravenous pharmacological sedation strategies in adults (aged ≥18 years) undergoing therapeutic gastrointestinal endoscopic procedures, including ERCP, ESD, or endoscopic mucosal resection (EMR). Eligible interventions included single-agent or combination regimens involving agents such as propofol, midazolam, dexmedetomidine, remimazolam, ketamine, fentanyl, or sufentanil. We excluded studies involving general anesthesia, endotracheal intubation, or neuromuscular blocking agents, as well as non-original articles (e.g., editorials, letters, case reports, conference abstracts, reviews, or meta-analyses).

### 2.2 Search strategy

We systematically searched PubMed, Embase, and the Cochrane Central Register of Controlled Trials (CENTRAL) from inception to 29 March 2025 to identify eligible RCTs. The search strategy incorporated both Medical Subject Headings (MeSH) and free-text terms related to therapeutic gastrointestinal endoscopy, sedative agents, and randomised study designs. Full search algorithms for each database are detailed in Supplementary Material S1 (Supplementary Table S1, pp 1–4). Diazepam was excluded from the search terms as it is no longer routinely used in therapeutic gastrointestinal endoscopy. Additionally, we also manually screened reference lists of relevant systematic reviews and all included studies to identify additional eligible trials.

### 2.3 Selection criteria

Two reviewers (HJ and YX) independently screened titles and abstracts of all retrieved records following manual removal of deduplicate citations. Full texts were obtained for studies that met eligibility criteria or required further assessment. Discrepancies were resolved through discussion or consultation with a third reviewer (IM). No language restrictions were applied. At the full-text stage, we excluded studies that assessed analgesic agents alone without sedative co-administration, as well as those using placebo or no intervention as the sole comparator.

### 2.4 Data extraction

Two reviewers (XQ and QL) independently extracted data from the included studies using a standardised data extraction form. The following information was collected: (i) Study characteristics: first author, year of publication, study setting, and sample size; (ii) Patient characteristics: age, sex, type of endoscopic procedure, and procedural time; (iii) Intervention details: induction regimen, maintenance regimen, and rescue regimen; (iv) Outcomes: procedural interference events, patient satisfaction, endoscopist satisfaction, induction time, hypoxia, hypotension, bradycardia, postoperative nausea and vomiting (PONV), and recovery time.

These outcomes were prespecified to reflect both the efficacy and safety dimensions of pharmacological sedation strategies. Outcome definitions followed those reported in the original studies; where multiple definitions were used, all were documented and summarised. No further reclassification was applied, except for procedural interference events, which were grouped under a unified conceptual category to enable consistent comparisons across studies. Procedural interference events were defined as patient-related events that interrupted or interfered with the endoscopic procedure, including coughing, body movement, agitation, or other reactions requiring temporary interruption of the procedure or additional sedation, as defined in the original trials. A complete list of outcome definitions and the corresponding value ranges is available in Supplementary Material S1 (Supplementary Table S2, pp 5–13).

Patient and endoscopist satisfaction were treated as continuous outcomes, and reported using various rating scales (e.g., 4-point Likert scale, 10-point numeric scale, 100-point visual analogue scale). To account for this heterogeneity, standardised mean differences (SMDs) were calculated for meta-analysis. In trials with multiple intervention arms, all eligible arms were included in the network. For outcomes reported only in graphical format, data were extracted using WebPlotDigitizer (https://apps.automeris.io/wpd4/). Any discrepancies in data extraction were resolved through discussion or consultation with a third reviewer (CG).

### 2.5 Risk of bias assessment

Two reviewers (HJ and YZ) independently assessed each study across the standard five domains using the Cochrane Risk of Bias 2.0 (RoB 2.0) tool (Sterne et al., 2019). Discrepancies were resolved through discussion or adjudication by a third reviewer (IM).

### 2.6 Certainty of evidence assessment

We assessed the certainty of evidence for each network comparison using the Grading of Recommendations, Assessment, Development, and Evaluation (GRADE) framework, as adapted for network meta-analyses by the GRADE Working Group (Puhan et al., 2014). Certainty ratings were classified as high (⊕⊕⊕⊕), moderate (⊕⊕⊕⊝), low (⊕⊕⊝⊝), or very low (⊕⊝⊝⊝). As all included studies were RCTs, the initial level for direct comparisons was categorised “high” but could be downgraded based on the following five domains: (i) ^*^Inconsistency, reflected by substantial variability in effect estimates across studies (e.g., *τ* ^2^, *I* ^2^, or Cochran’s Q); (ii) ^†^Indirectness, when study populations, interventions, or outcomes did not fully align with the target clinical question of interest; (iii) ^‡^Risk of bias, due to methodological limitations such as inadequate blinding or allocation concealment; (iv) ^¶^Imprecision, indicated by wide 95% confidence intervals (CIs) or estimates crossing the line of no effect; and (v) ^§^Publication bias, suggested by small-study effects or a P value <0.1 in Egger’s test.

### 2.7 Network geometry

We assessed the geometry of each outcome-specific network to evaluate the structure and completeness of the comparative evidence. In each graph, nodes represented interventions and edges indicated direct comparisons from at least one included trial. We examined the networks for dominant comparators, central hubs, and isolated nodes to understand the distribution of evidence and identify interventions contributing only indirect evidence.

### 2.8 Publication bias and meta-regression

To explore the presence of small-study effects indicative of publication bias, we visually inspected comparison-adjusted funnel plots for asymmetry within the network meta-analytic framework (Chaimani et al., 2013). In these plots, substantial asymmetry or a marked deviation of the central reference line–representing the adjusted average effect based on network-specific estimates that account for varying treatment comparisons–from the vertical axis may indicate publication bias or other small-study effects. The orientation of this line was also considered; a marked deviation from vertical may reflect inconsistency between treatment comparisons or other sources of systematic bias. Additionally, Egger’s regression test was applied to outcomes reported in at least 10 studies, using a P-value <0.1 to indicate potential small-study effects (Sterne et al., 2011).

For outcomes demonstrating substantial heterogeneity (defined as I^2^ > 50% or P value <0.1 from Cochran’s Q test), we conducted meta-regression analyses to explore potential sources of between-study variability. Prespecified covariates included type of endoscopic procedure (e.g., ERCP vs. ESD), mean patient age, procedural time, and overall risk of bias (categorised as low vs. unclear/high). Meta-regression was performed within a frequentist framework using the restricted maximum likelihood (REML) estimator. As prespecified, univariable meta-regression models were fitted for each covariate separately to avoid overfitting and facilitate interpretation.

### 2.9 Statistical analysis

To compare the efficacy and safety of all eligible pharmacological sedation strategies, we conducted a frequentist network meta-analysis using a generalised linear mixed model framework. Relative treatment effects were expressed as risk ratios (RRs) for binary outcomes and SMDs for continuous outcomes, each reported with corresponding 95% CIs. A common heterogeneity variance (*τ* ^2^) was assumed across all comparisons within each outcome-specific network.

To support a multidimensional assessment of benefit-risk trade-offs, we calculated surface under the cumulative ranking curve (SUCRA) values to summarise the relative ranking of interventions. Cluster plots were constructed to jointly visualise their comparative efficacy and safety; with treatments located in the favourable quadrant interpreted as demonstrating balanced performance across the examined outcomes. League tables were used to display pairwise relative effects derived from the network meta-analyses. For continuous outcomes, when standard deviations (SDs) were not reported, they were imputed following Cochrane Collaboration guidance (Higgins et al., 2024), using available statistics such as standard errors, confidence intervals, p-values, or interquartile ranges. When only medians and interquartile ranges were available, SDs were estimated using validated approximation methods.

In multi-arm trials, we accounted for the correlation between treatment arms to avoid unit-of-analysis errors. Network consistency was assessed using both global (design-by-treatment interaction model) and local (node-splitting method) approaches to detect disagreement between direct and indirect estimates (Jackson et al., 2014). If global inconsistency was detected (P < 0.05), we explored clinical and methodological heterogeneity across studies and then applied local node-splitting models. In the presence of unexplained and substantial inconsistency, we downgraded the certainty of evidence and interpreted findings with caution. For networks with persistent inconsistency, SUCRA rankings were withheld to avoid potentially misleading interpretations and instead provided a narrative synthesis or pairwise meta-analysis was instead provided. Heterogeneity was assessed using *τ* ^2^, Cochran’s Q statistic, and the I^2^ statistic (Higgins et al., 2012).

All analyses were conducted using validated software, including Stata version 16.0 (StataCorp LLC, College Station, TX, USA) and Review Manager (RevMan) version 5.4 (Cochrane Collaboration, London, UK). A two-sided P value < 0.05 was considered statistically significant.

## 3 Results

### 3.1 Study selection

**FIGURE 1:**
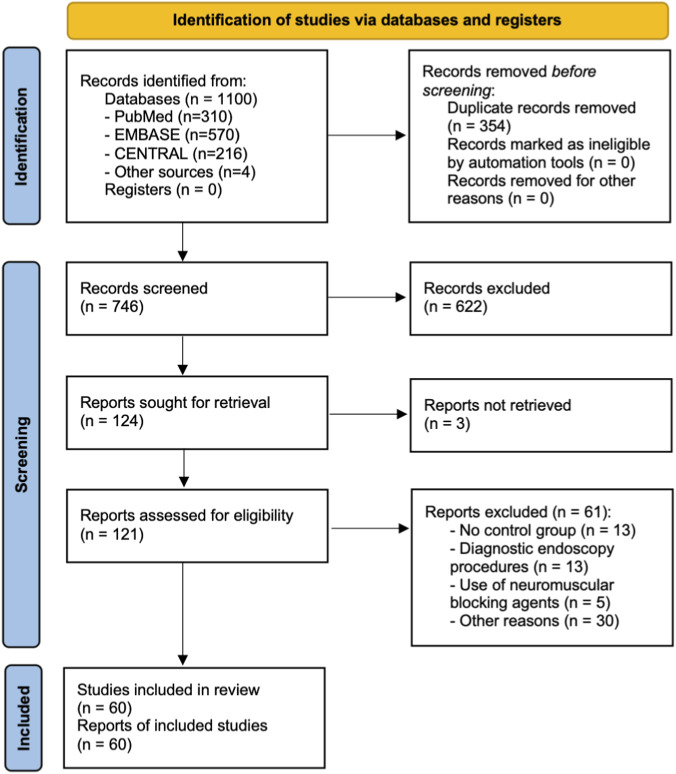
PRISMA flow diagram of the study selection process.

A total of 1,096 records were identified through electronic database searches, and an additional 4 records were retrieved by manually screening the reference lists of relevant reviews and included studies. After removing 354 duplicates, 742 unique records remained for title and abstract screening. Of these, 124 full-text articles were assessed for eligibility. Following full-text review, 60 RCTs involving 7,071 participants met the inclusion criteria (Supplementary Material S1; Supplementary Table S3, pp 14–19). The remaining 64 studies were excluded, with detailed reasons provided in Supplementary Material S1 (Supplementary Table S4, pp 20–25). A PRISMA flow diagram illustrating the study selection process is shown in Figure 1.

### 3.2 Study characteristics

The main characteristics of the 60 included RCTs are summarised in Supplementary Material S1 (Supplementary Table S5, pp 26–30). Collectively, these trials enrolled 7,071 patients undergoing therapeutic gastrointestinal endoscopic procedures, with the majority focusing on ERCP (n = 5,674) and ESD (n = 799). The remaining patients (n = 598) underwent other therapeutic interventions, such as mixed combinations of ESD, ERCP, or EMR.

Across the included studies, 32 distinct pharmacological sedation regimens were evaluated. The most frequently studied strategies were propofol-opioid combinations (n = 1,647), followed by midazolam-opioid combinations (n = 857), midazolam-propofol-opioid combinations (n = 747), and propofol monotherapy (n = 664). A complete list of interventions is provided in Supplementary Material S1 (Supplementary Table S6, p 31). Opioids agents included commonly used drugs such as fentanyl and sufentanil. Details on the types and dosing regimens of all sedative agents are summarised in Supplementary Material S1 (Supplementary Table S7, pp 32–38).

### 3.3 Risk of bias

Based on overall assessments across the seven domains of the Cochrane Risk of Bias tool, 16 studies were judged to be at low risk of bias, A further 18 were rated as having an unclear risk of bias, and 26 were deemed to be at high risk of bias. These assessments were incorporated into the GRADE certainty ratings for each network comparison. The results of the risk-of-bias assessment are provided in Supplementary Material S1 (Supplementary Table S8; Supplementary Figure S1, pp 39–40).

### 3.4 Overview of outcome analyses

This network meta-analysis initially designed to evaluate nine outcomes: procedural interference events, induction time, patient satisfaction, endoscopist satisfaction, hypoxia, hypotension, bradycardia, PONV, and recovery time. However, network consistency could not be established for several endpoints. Specifically, global inconsistency tests using the design-by-treatment interaction model revealed statistically significant inconsistency for induction time, patient satisfaction, and endoscopist satisfaction (P < 0.05 for all), thereby precluding valid network synthesis. Additionally, the network structure for PONV was fragmented, comprising three disconnected subnetworks, rendering a unified analysis infeasible.

Consequently, only five outcomes–procedural interference events, hypoxia, hypotension, bradycardia, and recovery time–were included in the network meta-analysis. The remaining outcomes were synthesised narratively based on direct comparisons. In particular, comparisons between commonly used clinical regimens, such as ketamine-propofol and propofol-opioid sedation, were emphasised in the interpretation of network estimates. Comparison-adjusted funnel plots and Egger’s test results for the five analysed outcomes are presented in Supplementary Material S1 (Supplementary Figures S2–S6; Supplementary Table S9, pp 41–43), and complete league tables of treatment effects are provided in Supplementary Material S2.

### 3.5 Procedural interference events

**FIGURE 2:**
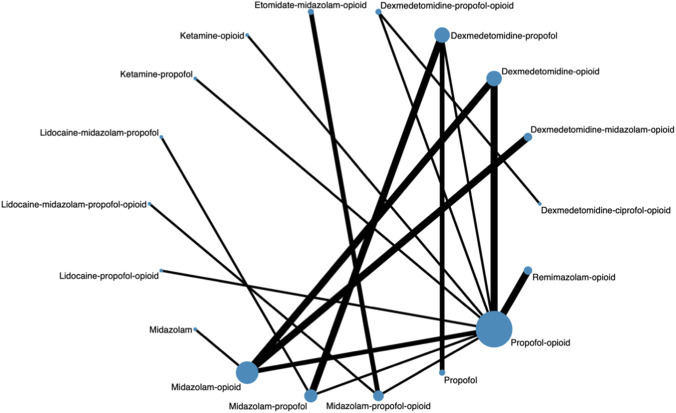
Network plot of pharmacological sedation strategies for procedural interference events.

Procedural interference events reported in the included trials mainly comprised patient movement, coughing, or other reactions requiring temporary interruption of the procedure or additional sedation. Twenty-nine RCTs involving 3,276 patients reported data on procedural interference events, evaluating 20 pharmacological sedation regimens. One study (n = 60) was excluded from the network meta-analysis as it comprised an isolated comparison (ketamine-etomidate-midazolam vs. ketamine-dexmedetomidine-midazolam) that could not be connected to the broader network (Singh et al., 2023). The final network was fully connected and comprised 18 interventions (Figure 2).

Compared with propofol-opioid sedation, none of the evaluated interventions demonstrated a statistically significant reduction in procedural interference events. However, two regimens ranked highest in SUCRA probability and demonstrated large, albeit non-significant, effect estimates: lidocaine-propofol-opioid ranked first (SUCRA: 91.8%; RR: 0.13, 95% CI: 0.02 to 1.07; P = 0.057; low certainty), and lidocaine-midazolam-propofol-opioid ranked second (SUCRA: 86.3%; RR: 0.24, 95% CI: 0.05 to 1.10; P = 0.066; very low certainty). In contrast, midazolam-opioid ranked lowest in SUCRA probability (8.1%) and was associated with a significantly higher risk of procedural interference compared with propofol-opioid (RR: 2.98, 95% CI: 1.55 to 5.73; P = 0.001; low certainty) (Table 1).

**TABLE 1:** Relative risks, SUCRA rankings, and GRADE certainty of evidence of pharmacological sedation strategies for procedural interference events.

Procedural interference events	*τ* ^2^ = 0.200; *τ* = 0.447; *I* ^2^ = 42.0%; *P* = 0.040 at Cochran’s Q test				
Treatment	RR	95% CI	P-value	SUCRA	QoE
Lidocaine–propofol–opioid	0.13	(0.02 to 1.07)	0.057	91.8	⊕⊕⊝⊝ low^*,¶^
Lidocaine–midazolam–propofol–opioid	0.24	(0.05 to 1.10)	0.066	86.3	⊕⊝⊝⊝ very low^*,†,¶^
Dexmedetomidine–ciprofol–opioid	0.34	(0.05 to 2.18)	0.253	78.8	⊕⊝⊝⊝ very low^*,†,‡,¶^
Dexmedetomidine–propofol–opioid	0.40	(0.07 to 2.18)	0.290	74.8	⊕⊝⊝⊝ very low^*,†,¶^
Dexmedetomidine–propofol	0.56	(0.17 to 1.88)	0.350	70.1	⊕⊝⊝⊝ very low^*,‡,¶^
Etomidate–midazolam–opioid	0.68	(0.25 to 1.86)	0.449	63.5	⊕⊝⊝⊝ very low^†,‡,¶^
Ketamine–propofol	0.67	(0.17 to 2.65)	0.565	61.1	⊕⊝⊝⊝ very low^*,‡,¶^
Midazolam–propofol–opioid	0.79	(0.36 to 1.74)	0.558	57.6	⊕⊕⊝⊝ low^*,¶^
Lidocaine–midazolam–propofol	0.77	(0.17 to 3.60)	0.743	57.2	⊕⊝⊝⊝ very low^*,†,‡,¶^
Propofol–opioid	–	–	–	49.0	​
Dexmedetomidine–opioid	1.19	(0.56 to 2.55)	0.646	42.1	⊕⊝⊝⊝ very low^*,†,‡,¶^
Dexmedetomidine–midazolam–opioid	1.45	(0.64 to 3.28)	0.373	33.3	⊕⊝⊝⊝ very low^*,†,¶^
Ketamine–opioid	1.41	(0.70 to 2.88)	0.338	33	⊕⊝⊝⊝ very low^*,‡,¶^
Midazolam	1.99	(0.28 to 14.14)	0.493	28.8	⊕⊝⊝⊝ very low^*,†,‡,¶^
Remimazolam–opioid	1.71	(0.83 to 3.54)	0.144	25.3	⊕⊝⊝⊝ very low^*,‡,¶^
Midazolam–propofol	1.93	(0.62 to 5.99)	0.254	22.8	⊕⊕⊝⊝ very low^*,‡,¶^
Propofol	2.79	(0.48 to 16.18)	0.252	16.6	⊕⊝⊝⊝ very low^*,†,‡,¶^
**Midazolam–opioid**	**2.98**	**(1.55 to 5.73)**	**0.001**	**8.1**	**⊕⊕⊝⊝ low** ^*,†,‡^

Bold values indicate statistically significant results (P < 0.05).

* Inconsistency; † Indirectness; ‡ Risk of bias; ¶ Imprecision; § Publication bias.

Additional details for procedural interference events are provided in Supplementary Material S1 (Supplementary Tables S10–S12; Supplementary Figure S7, pp 44–45).

### 3.6 Cardiorespiratory adverse events

Fifty RCTs (n = 6,338) reported data on hypoxia, evaluating 29 pharmacological sedation regimens. Ketamine-dexmedetomidine ranked highest in SUCRA (90.1%) and was associated with the greatest reduction in hypoxia risk (RR: 0.03; 95% CI: 0.01 to 0.48; P = 0.014; low certainty). Lidocaine-midazolam-propofol ranked second (SUCRA: 87.3%) and also showed significant benefit (RR: 0.05; 95% CI: 0.01 to 0.46; P = 0.008; low certainty). Ketamine-propofol ranked third (SUCRA: 76.7%) and was associated with a significant reduction in hypoxia (RR: 0.12; 95% CI: 0.03 to 0.59; P = 0.009; moderate certainty) (Table 2). Additional details for hypoxia are provided in Supplementary Material S1 (Supplementary Tables S13–S15; Supplementary Figures S8–S9, pp 46–48).

**TABLE 2:** Relative risks, SUCRA values, and GRADE certainty of evidence for pharmacological sedation strategies for cardiorespiratory adverse events.

Hypoxia	*τ* ^2^ = 0.110; *τ* = 0.332; *I* ^2^ = 22.0%; *P* = 0.150 at Cochran’s Q test				
Treatment	RR	95% CI	*P*-value	SUCRA	QoE
**Ketamine–dexmedetomidine**	**0.03**	**(0.01 to 0.48)**	**0.014**	**90.1**	⊕⊕⊝⊝ low^‡,§^
**Lidocaine–midazolam–propofol**	**0.05**	**(0.01 to 0.46)**	**0.008**	**87.3**	⊕⊕⊝⊝ low^†,§^
**Ketamine–propofol**	**0.12**	**(0.03 to 0.59)**	**0.009**	**76.7**	⊕⊕⊕⊝ moderate^§^
Ketamine–etomidate–midazolam	0.11	(0.01 to 2.13)	0.196	71.7	⊕⊝⊝⊝ very low^†,¶,§^
**Dexmedetomidine–ciprofol–opioid**	**0.15**	**(0.03 to 0.93)**	**0.041**	**71.2**	⊕⊝⊝⊝ very low^†,‡,§^
**Dexmedetomidine–opioid**	**0.18**	**(0.05 to 0.65)**	**0.009**	**70.2**	⊕⊕⊝⊝ low^‡,§^
**Ketamine–dexmedetomidine–midazolam**	0.16	(0.02 to 1.68)	0.127	66.5	⊕⊝⊝⊝ very low^†,‡,¶,§^
**Dexmedetomidine–midazolam–opioid**	**0.21**	**(0.07 to 0.65)**	**0.007**	**65.8**	⊕⊝⊝⊝ very low^†,‡,§^
Etomidate–midazolam–opioid	0.19	(0.02 to 1.44)	0.109	64.4	⊕⊝⊝⊝ very low^†,‡,¶,§^
Ketamine–midazolam–propofol	0.21	(0.03 to 1.62)	0.135	61.1	⊕⊝⊝⊝ very low^†,¶,§^
**Ketamine–opioid**	**0.25**	**(0.06 to 0.98)**	**0.046**	**60.2**	⊕⊕⊝⊝ low^‡,§^
**Dexmedetomidine–propofol**	**0.27**	**(0.09 to 0.82)**	**0.021**	**58.3**	⊕⊝⊝⊝ very low^†,‡,§^
Midazolam–propofol	0.26	(0.06 to 1.11)	0.069	58.2	⊕⊝⊝⊝ very low^†,‡,¶,§^
**Dexmedetomidine**	**0.29**	**(0.09 to 0.99)**	**0.048**	**57.1**	⊕⊝⊝⊝ very low^†,‡,§^
Dexmedetomidine–midazolam	0.21	(0.01 to 18.07)	0.494	56.4	⊕⊝⊝⊝ very low^†,¶,§^
Dexmedetomidine–midazolam–propofol	0.24	(0.01 to 9.41)	0.442	54.4	⊕⊝⊝⊝ very low^†,¶,§^
Ketamine–propofol–opioid	0.36	(0.13 to 1.00)	0.050	48.9	⊕⊕⊝⊝ low^¶,§^
**Remimazolam–opioid**	**0.46**	**(0.25 to 0.84)**	**0.012**	**41.7**	⊕⊕⊝⊝ low^‡,§^
Ciprofol–opioid	0.47	(0.15 to 1.42)	0.180	41.1	⊕⊕⊝⊝ low^¶,§^
Remimazolam	0.52	(0.08 to 3.49)	0.504	38.6	⊕⊝⊝⊝ very low^†,‡,¶,§^
Propofol	0.52	(0.23 to 1.20)	0.125	36.4	⊕⊝⊝⊝ very low^†,‡,¶,§^
Lidocaine–midazolam–propofol–opioid	0.54	(0.18 to 1.66)	0.284	35.3	⊕⊝⊝⊝ very low^†,¶,§^
Lidocaine–propofol–opioid	0.69	(0.11 to 4.40)	0.694	29.5	⊕⊕⊝⊝ low^¶,§^
Dexmedetomidine–propofol–opioid	0.72	(0.24 to 2.18)	0.562	27.3	⊕⊕⊝⊝ low^¶,§^
Midazolam–opioid	0.74	(0.42 to 1.31)	0.305	24.2	⊕⊝⊝⊝ very low^‡,¶,§^
Midazolam	1.02	(0.30 to 3.45)	0.971	17.1	⊕⊝⊝⊝ very low^†,‡,¶,§^
Propofol–opioid	–	–	–	14.2	​
Midazolam–propofol–opioid	0.99	(0.47 to 2.09)	0.989	14.1	⊕⊝⊝⊝ very low^†,¶,§^
Esktamine–propofol	1.31	(0.43 to 3.95)	0.633	11.9	⊕⊕⊝⊝ low^¶,§^

Hypotension	*τ* ^2^ = 0.140; *τ* = 0.374; *I* ^2^ = 35.0%; *P* = 0.050 at Cochran’s Q test				
Treatment	RR	95%CI	*P*-value	SUCRA	QoE
Ketamine–dexmedetomidine	0.06	(0.01 to 1.06)	0.054	90.4	⊕⊝⊝⊝ very low^*,‡,¶,§^
**Lidocaine–midazolam–propofol**	**0.08**	**(0.01 to 0.92)**	**0.043**	**89.6**	⊕⊝⊝⊝ very low^*,†,§^
**Ketamine–propofol**	**0.28**	**(0.09 to 0.83)**	**0.021**	**80.8**	⊕⊕⊝⊝ low^*,§^
**Remimazolam–opioid**	**0.46**	**(0.31 to 0.69)**	**<0.001**	**70.3**	⊕⊝⊝⊝ very low^*,‡,§^
Remimazolam	0.37	(0.01 to 10.76)	0.564	62.9	⊕⊝⊝⊝ very low^*,†,‡,¶,§^
Etomidate–midazolam–opioid	0.50	(0.10 to 2.59)	0.407	62.6	⊕⊝⊝⊝ very low^*,†,‡,¶,§^
Esktamine–propofol	0.56	(0.23 to 1.34)	0.192	62.4	⊕⊝⊝⊝ very low^*,¶,§^
Dexmedetomidine–propofol–opioid	0.63	(0.29 to 1.38)	0.248	58.4	⊕⊝⊝⊝ very low^*,¶,§^
Midazolam	0.64	(0.20 to 1.99)	0.438	58.4	⊕⊝⊝⊝ very low^*,†,‡,¶,§^
Midazolam–propofol	0.65	(0.19 to 2.22)	0.492	57	⊕⊝⊝⊝ very low^*,‡,¶,§^
Dexmedetomidine–midazolam–propofol	0.65	(0.01 to 39.23)	0.837	51.9	⊕⊝⊝⊝ very low^*,†,¶,§^
Ketamine–midazolam–propofol	0.65	(0.01 to 39.23)	0.837	51.9	⊕⊝⊝⊝ very low^*,†,¶,§^
Dexmedetomidine-midazolam	0.65	(0.01 to 189.07)	0.882	49.5	⊕⊝⊝⊝ very low^*,†,‡,¶,§^
Ciprofol–opioid	0.87	(0.44 to 1.71)	0.689	62.4	⊕⊝⊝⊝ very low^*,¶,§^
Propofol–opioid	–	–	–	39.5	​
Dexmedetomidine–opioid	1.06	(0.42 to 2.66)	0.905	38.5	⊕⊝⊝⊝ very low^*,‡,¶,§^
Propofol	1.12	(0.42 to 2.99)	0.829	36.3	⊕⊝⊝⊝ very low^*,†,‡,¶,§^
Lidocaine–midazolam–propofol–opioid	1.17	(0.35 to 3.92)	0.801	34.2	⊕⊝⊝⊝ very low^*,†,¶,§^
Midazolam–opioid	1.24	(0.70 to 2.19)	0.468	30.6	⊕⊝⊝⊝ very low^*,‡,¶,§^
Dexmedetomidine	1.43	(0.40 to 5.14)	0.580	27.1	⊕⊝⊝⊝ very low^*,†,‡,¶,§^
Dexmedetomidine–propofol	1.57	(0.52 to 4.76)	0.429	23.1	⊕⊝⊝⊝ very low^*,‡,¶,§^
Dexmedetomidine–midazolam–opioid	1.73	(0.55 to 5.38)	0.346	21.5	⊕⊝⊝⊝ very low^*,†,‡,¶,§^
Midazolam–propofol–opioid	2.39	(0.99 to 5.78)	0.052	9.3	⊕⊝⊝⊝ very low^*,¶,§^

Bradycardia	*τ* ^2^ = 0.510; *τ* = 0.714; *I* ^2^ = 56.0%; *P* = 0.001 at Cochran’s Q test				
Treatment	RR	95%CI	*P*-value	SUCRA	QoE
**Ketamine–propofol**	**0.11**	**(0.01 to 0.86)**	**0.035**	**85.0**	⊕⊕⊕⊝ moderate^*^
Lidocaine–midazolam–propofol–opioid	0.14	(0.01 to 3.60)	0.233	79.9	⊕⊝⊝⊝ very low^*,†,¶^
Etomidate–midazolam–opioid	0.19	(0.02 to 1.76)	0.143	78.3	⊕⊝⊝⊝ very low^*,†,‡,¶^
Ketamine–dexmedetomidine	0.20	(0.01 to 4.14)	0.301	73.1	⊕⊝⊝⊝ very low^*,‡,¶^
**Remimazolam–opioid**	**0.45**	**(0.30 to 0.69)**	**<0.001**	**67.5**	⊕⊕⊝⊝ low^*,‡^
Midazolam	0.31	(0.01 to 9.01)	0.492	67.3	⊕⊝⊝⊝ very low^*,†,‡,¶^
Midazolam–propofol–opioid	0.68	(0.18 to 2.60)	0.578	54.2	⊕⊝⊝⊝ very low^*,¶^
Midazolam–opioid	0.72	(0.41 to 1.25)	0.245	53.7	⊕⊝⊝⊝ very low^*,‡,¶^
Dexmedetomidine–opioid	0.81	(0.08 to 7.88)	0.857	49.2	⊕⊝⊝⊝ very low^*,‡,¶^
Remimazolam	0.92	(0.10 to 8.76)	0.939	46.9	⊕⊝⊝⊝ very low^*,†,‡,¶^
Propofol	0.92	(0.28 to 2.99)	0.884	46.8	⊕⊝⊝⊝ very low^*,†,‡,¶^
Propofol–opioid	–	–	–	42.8	​
Dexmedetomidine–midazolam–propofol	1.45	(0.01 to 269.83)	0.890	42	⊕⊝⊝⊝ very low^*,†,¶^
Dexmedetomidine–midazolam	1.45	(0.01 to 977.68)	0.912	41.6	⊕⊝⊝⊝ very low^*,†,‡,¶^
Ketamine–midazolam–propofol	1.45	(0.01 to 269.79)	0.890	40.3	⊕⊝⊝⊝ very low^*,†,‡,¶^
Midazolam–propofol	1.45	(0.04 to 48.14)	0.837	39.6	⊕⊝⊝⊝ very low^*,†,‡,¶^
Esktamine–propofol	1.43	(0.25 to 8.32)	0.692	35.5	⊕⊕⊝⊝ low^*,¶^
Dexmedetomidine–midazolam–opioid	1.86	(0.46 to 7.58)	0.388	27.5	⊕⊝⊝⊝ very low^*,†,‡,¶^
**Dexmedetomidine–propofol–opioid**	**3.55**	**(1.84 to 6.84)**	**<0.001**	**15.3**	⊕⊕⊕⊝ moderate^*^
Dexmedetomidine–propofol	4.34	(0.92 to 20.51)	0.064	13.5	⊕⊝⊝⊝ very low^*,†,‡,¶^

Bold values indicate statistically significant results (P < 0.05).

* Inconsistency; † Indirectness; ‡ Risk of bias; ¶ Imprecision; § Publication bias.

Forty-seven RCTs (n = 5,653) reported on hypotension across 25 sedation regimens. One study was excluded due to an isolated comparator (ketamine-etomidate-midazolam vs. ketamine-dexmedetomidine-midazolam) (Singh et al., 2023). The final network comprised 23 interventions and was fully connected. Lidocaine-midazolam-propofol ranked second in SUCRA (89.6%) and significantly reduced the risk of hypotension vs. propofol-opioid (RR: 0.08; 95% CI: 0.01 to 0.92; P = 0.043; very low certainty). Ketamine-propofol also demonstrated benefit (SUCRA: 80.8%; RR: 0.28; 95% CI: 0.09 to 0.83; P = 0.021; low certainty), and remimazolam-opioid showed protective effects (SUCRA: 70.3%; RR: 0.46; 95% CI: 0.31 to 0.69; P < 0.001; very low certainty) (Table 2). Additional details for hypotension are provided in Supplementary Material S1 (Supplementary Tables S16–S18; Supplementary Figures S10–S11, pp 49–51).

Thirty-six RCTs (n = 4,324) evaluated bradycardia across 26 regimens. One trial was excluded due to an isolated node (ketamine-etomidate-midazolam vs. ketamine-dexmedetomidine-midazolam), resulting in a final network of 25 connected interventions (Singh et al., 2023). Ketamine-propofol ranked highest (SUCRA: 85.0%) and significantly reduced bradycardia vs. propofol-opioid (RR: 0.11; 95% CI: 0.01 to 0.86; P = 0.035; moderate certainty). Remimazolam-opioid also reduced bradycardia risk (SUCRA: 67.5%; RR: 0.45; 95% CI: 0.30 to 0.69; P < 0.001; low certainty) (Table 2). Additional details for bradycardia are provided in Supplementary Material S1 (Supplementary Tables S19–S21; Supplementary Figures S12–S13, pp 52–54).

### 3.7 Recovery time

A total of 46 RCTs involving 6,085 patients evaluated 30 pharmacological sedation regimens for recovery time. Ketamine-propofol-opioid ranked highest in SUCRA (99.7%) and demonstrated the most substantial reduction in recovery time (SMD: −3.15; 95% CI: −4.55 to −1.75; P < 0.001; low certainty). In contrast, dexmedetomidine-midazolam-propofol ranked lowest (SUCRA: 2.2%) and was associated with the longest recovery duration (SMD: 2.95; 95% CI: 1.30 to 4.60; P < 0.001; very low certainty). Dexmedetomidine-dexmedetomidine-midazolam also resulted in a significantly prolonged recovery time (SUCRA: 3.3%; SMD: 2.76; 95% CI: 0.84 to 4.68; P = 0.005; very low certainty) (Table 3). Additional details for recovery time are provided in Supplementary Material S1 (Supplementary Tables S22–S24; Supplementary Figures S14–S15, pp 55–57).

**TABLE 3:** Relative risks, SUCRA values, and GRADE certainty of evidence for pharmacological sedation strategies for recovery time.

Recovery time	*τ* ^2^ = 0.410; *τ* = 0.640; *I* ^2^ = 51.0%; *P* = 0.001 at Q test				
Treatment	SMD	95%CI	P-value	SUCRA	QoE
**KET-PF-OP**	**−3.15**	**(−4.55 to −1.75)**	**<0.001**	**99.7**	⊕⊕⊝⊝ low^*,§^
DEX	−1.03	(−2.70 to 0.65)	0.229	84.6	⊕⊝⊝⊝ very low^*,†,‡,¶,§^
CFL-OP	−0.75	(−2.13 to 0.63)	0.287	77.6	⊕⊝⊝⊝ very low^*,¶,§^
PF	−0.35	(−1.29 to 0.59)	0.465	72	⊕⊝⊝⊝ very low^*,†,‡,¶,§^
KET-DEX-MDZ-PF-OP	−0.51	(−2.35 to 1.32)	0.582	71.3	⊕⊝⊝⊝ very low^*,†,‡,¶,§^
RMZ-OP	−0.33	(−0.96 to 0.29)	0.297	70.9	⊕⊝⊝⊝ very low^*,‡,¶,§^
KET-MDZ-PF-OP	−0.51	(−2.35 to 1.32)	0.582	70.8	⊕⊝⊝⊝ very low^*,†,‡,¶,§^
LIDO-MDZ-PF	−0.32	(−2.05 to 1.41)	0.718	65.5	⊕⊝⊝⊝ very low^*,†,‡,¶,§^
DEX-PF	−0.18	(−1.20 to 0.84)	0.728	65.1	⊕⊝⊝⊝ very low^*,‡,¶,§^
LIDO-MDZ-PF-OP	−0.11	(−1.39 to 1.17)	0.867	61.9	⊕⊝⊝⊝ very low^*,†,¶,§^
PF-OP	–	–	–	58.4	​
LIDO-PF-OP	0	(−1.45 to 1.45)	1.000	57.1	⊕⊝⊝⊝ very low^*,¶,§^
ESK-PF	0	(−1.39 to 1.39)	1.000	56.1	⊕⊝⊝⊝ very low^*,¶,§^
DEX-MDZ-OP	0.15	(−0.92 to 1.22)	0.779	53.7	⊕⊝⊝⊝ very low^*,†,¶,§^
KET-PF	0.13	(−1.25 to 1.52)	0.852	52.6	⊕⊝⊝⊝ very low^*,¶,§^
KET-ETM-MDZ	0.23	(−2.21 to 2.68)	0.851	49.8	⊕⊝⊝⊝ very low^*,†,¶,§^
DEX-PF-OP	0.24	(−0.79 to 1.26)	0.650	49.4	⊕⊝⊝⊝ very low^*,¶,§^
RMZ	0.41	(−1.28 to 2.11)	0.634	42.8	⊕⊝⊝⊝ very low^*,†,‡,¶,§^
DEX-MDZ	0.50	(−1.42 to 2.42)	0.612	42.3	⊕⊝⊝⊝ very low^*,†,‡,¶,§^
ETM-MDZ-OP	0.50	(−0.76 to 1.75)	0.439	39.3	⊕⊝⊝⊝ very low^*,†,‡,¶,§^
MDZ	0.59	(−1.06 to 2.25)	0.481	38.4	⊕⊝⊝⊝ very low^*,†,‡,¶,§^
MDZ-PF	0.48	(−0.49 to 1.46)	0.332	38.2	⊕⊝⊝⊝ very low^*,¶,§^
MDZ-OP	0.61	(−0.25 to 1.46)	0.163	33.6	⊕⊝⊝⊝ very low^*,¶,§^
KET-OP	0.73	(−0.71 to 2.17)	0.323	32.3	⊕⊝⊝⊝ very low^*,‡,¶,§^
DEX-CFL-OP	0.84	(−0.88 to 2.55)	0.340	32.1	⊕⊝⊝⊝ very low^*,†,‡,¶,§^
MDZ-PF-OP	00.65	(−0.12 to 1.42)	0.100	31.4	⊕⊝⊝⊝ very low^*,¶,§^
KET-DEX	0.99	(−0.44 to 2.42)	0.175	26.4	⊕⊝⊝⊝ very low^*,‡,¶,§^
KET-MDZ-PF	1.07	(−0.19 to 2.34)	0.095	21.5	⊕⊝⊝⊝ very low^*,†,‡,¶,§^
**KET-DEX-MDZ**	**2.76**	**(0.84 to 4.68)**	**0.005**	**3.3**	⊕⊝⊝⊝ very low^*,†,‡,§^
**DEX-MDZ-PF**	**2.95**	**(1.30 to 4.60)**	**<0.001**	**2.2**	⊕⊝⊝⊝ very low^*,†,‡,§^

Bold values indicate statistically significant results (P < 0.05).

* Inconsistency; † Indirectness; ‡ Risk of bias; ¶ Imprecision; § Publication bias.

### 3.8 Cluster ranking analyses

**FIGURE 3:**
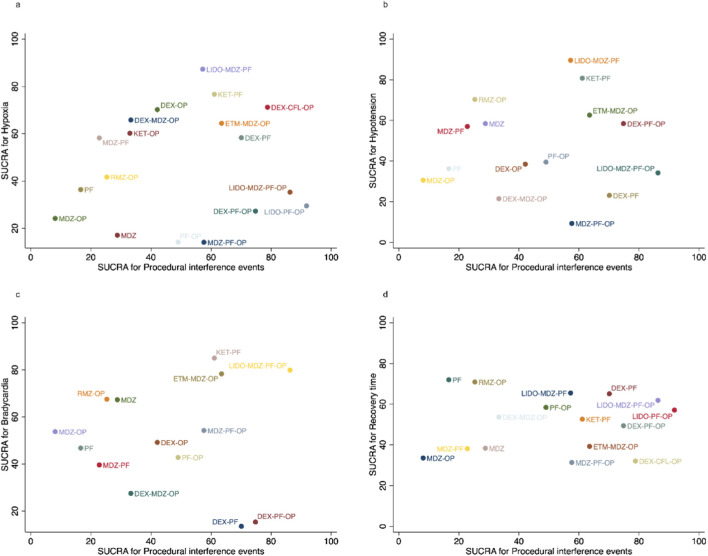
Cluster ranking plots of SUCRA values for procedural interference events and safety outcomes: **(a)** hypoxia, **(b)** hypotension, **(c)** bradycardia, and **(d)** recovery time.

Cluster ranking analyses jointly compared procedural interference events alongside hypoxia, hypotension, bradycardia, and recovery time. Lidocaine-midazolam-propofol and ketamine-propofol consistently appeared in the favourable quadrant for hypoxia (Figure 3a) and ranked highly for hypotension (Figure 3b). For bradycardia (Figure 3c), lidocaine-midazolam-propofol-opioid and ketamine-propofol showed favourable results. Notably, ketamine-propofol performed consistently well across all three cardiorespiratory comparisons. In comparison with recovery time (Figure 3d), lidocaine-propofol-opioid and lidocaine-midazolam-propofol-opioid clustered within the favourable quadrant.

### 3.9 Meta regression

None of the prespecified covariates–including procedure type, mean patient age, procedural time, and overall risk of bias–showed a statistically significant association with these outcomes analysed (all P > 0.05). Overall risk of bias showed a borderline association with bradycardia (t = −1.950, P = 0.059). Meta-regression was not performed for hypoxia due to low heterogeneity (*τ* ^*2*^ = 0.110; *I* ^*2*^ = 22.0%; P = 0.150 from Cochran’s Q test). Full regression results are provided in Supplementary Material S1 (Supplementary Table S25, p 58).

### 3.10 Narrative synthesis for unanalysed outcomes

As noted above, induction time, patient satisfaction, endoscopist satisfaction, and PONV were excluded from in the network meta-analysis due to methodological limitations. This section provides a narrative synthesis of the available findings from direct comparisons involving propofol-opioid across eligible studies.

For induction time, eight studies provided direct comparisons using propofol-opioid as the reference, evaluating seven alternative sedation regimens (Ding et al., 2024; Dong et al., 2023; Han et al., 2019; Kim et al., 2016; Lee et al., 2015; Srivastava et al., 2021; Tian et al., 2024; Zhou et al., 2024). Among these, ciprofol-opioid, dexmedetomidine-opioid, and remimazolam-opioid were associated with longer induction times compared with propofol-opioid. In contrast, lidocaine-propofol-opioid demonstrated a shorter induction time.

For endoscopist satisfaction, eleven studies provided direct comparisons using propofol-opioid as the reference, evaluating eleven alternative sedation regimens. Among these, dexmedetomidine-propofol-opioid was associated with higher satisfaction scores compared with propofol-opioid, whereas ketamine-opioid and midazolam-propofol were associated with lower satisfaction scores.

For patient satisfaction, ten studies provided direct comparisons using propofol-opioid as the reference, involving nine alternative sedation regimens. Midazolam-propofol-opioid was associated with higher patient satisfaction than propofol-opioid. For remimazolam-opioid, findings were inconsistent across two studies–one reported superior satisfaction compared with propofol-opioid, while the other found no significant difference.

For PONV, seven studies reported direct comparisons using propofol-opioid as the reference, encompassing seven alternative sedation regimens. Ketamine-propofol-opioid was associated with a lower incidence of PONV compared with propofol-opioid.

## 4 Discussion

### 4.1 Summary of key findings

In this comprehensive analysis of 60 randomised trials (57 included in the network meta-analysis) comprising 7,071 patients undergoing therapeutic gastrointestinal endoscopy, we compared the efficacy and safety profiles of 32 pharmacological sedation strategies. For complex procedures such as ERCP and ESD, no regimen significantly outperformed propofol-opioid in reducing procedural interference events, although combinations such as lidocaine-propofol-opioid and lidocaine-midazolam-propofol-opioid demonstrated favourable trends.

Regarding cardiorespiratory adverse events, ketamine-propofol consistently demonstrated superior safety across outcomes including hypoxia, hypotension, and bradycardia. Remimazolam-opioid also showed favourable effects in mitigating these events. Regarding recovery time, ketamine-propofol-opioid was associated with the most rapid emergence, whereas dexmedetomidine-containing regimens tended to delay recovery.

These findings suggest that while propofol-opioid remains an effective reference standard, specific alternative regimens may offer a more favourable balance between procedural efficacy and physiological stability, particularly in higher-risk or prolonged endoscopic interventions. Cluster ranking analyses supported these conclusions, with ketamine-propofol consistently occupying favourable quadrants across multiple benefit-risk comparisons. Collectively, these results provide an evidence-based framework for tailoring sedation strategies to the procedural and physiological demands of advanced endoscopic techniques.

### 4.2 Comparison with previous literature

In contrast to previous network meta-analyses which predominantly addressed diagnostic or short-duration endoscopic procedures, our study specifically focused specifically on therapeutic interventions such as ERCP and ESD, which are more invasive, prolonged, and physiologically demanding. Notably, a recent large-scale network meta-analysis published in 2025 evaluated 152 RCTs but excluded ERCP entirely and limited inclusion to procedures typically lasting less than 30 min (Li et al., 2025). Although that analysis reaffirmed propofol-opioid as the reference standard and identified etomidate-opioid and esketamine-remimazolam as promising alternatives, its findings may not be applicable to therapeutic endoscopy. Our study builds upon this previous work by including 60 trials exclusively focused on therapeutic procedures, thereby addressing a significant evidence gap in procedural sedation. This procedure-specific approach ensures that our findings more accurately reflect the sedation requirements and physiological challenges inherent to complex interventions such as ERCP and ESD. While the methodological framework was broadly comparable to earlier analyses, the targeted focus of our study enhances the clinical relevance of the findings within real-world therapeutic settings.

Several studies have evaluated sedation regimens for ERCP using both pairwise and network meta-analyses. A recent network meta-analysis dedicated to ERCP identified ketamine-propofol as a high-performing regimen for safety outcomes, aligning with our findings, although it did not evaluate ESD or recovery-related endpoints (Liu et al., 2025). Similarly, a pairwise meta-analysis comparing propofol-ketamine versus propofol-fentanyl similarly reported reduced lower incidences of hypotension and postoperative nausea in the ketamine group, consistent with our findings regarding the favourable hemodynamic profile of ketamine-based combinations (Rees et al., 2025).

Beyond the selection of pharmacological agents, comparative studies have also explored sedation depth and airway management. Prior reviews comparing monitored anesthesia care (MAC) with general anesthesia for ERCP have demonstrated comparable safety profiles, with MAC associated with faster recovery (Dhaliwal et al., 2021). As the majority of sedation regimens evaluated in our network fall within the MAC paradigm, our findings offer clinically actionable insights for optimizing pharmacologic strategies in MAC without resorting to general anesthesia. Likewise, a meta-analysis comparing intubated versus non-intubated approaches during ERCP underscored the trade-off between hypoxia prevention and recovery delay—further reinforcing the imperative to optimise pharmacological regimens that balance efficacy, safety, and recovery without invasive airway management (Zhang et al., 2025).

### 4.3 Potential mechanisms and interpretations

The differential efficacy and safety profiles observed across sedation regimens may be attributed to distinct pharmacodynamic properties and interactions. For instance, ketamine-propofol and lidocaine-midazolam-propofol consistently demonstrated favourable outcomes across multiple domains. The synergistic interaction between ketamine and propofol–commonly referred to as “ketofol” – is known to provide balanced sedation and analgesia while attenuating the hypotension and respiratory depression commonly associated with propofol monotherapy (Green et al., 2015; Jalili et al., 2016). Ketamine’s sympathomimetic properties likely contribute to its reduced incidence of hypotension and bradycardia, while its NMDA receptor antagonism may provide analgesic advantages, reducing patient movement during complex procedures (Zanos et al., 2018; Doleman et al., 2023). However, ketamine’s dissociative properties may increase the risk of emergence phenomena, such as hallucinations, dysphoria, and agitation, particularly when used at higher doses or in susceptible populations including critically ill patients (Gitlin et al., 2020; Serpa et al., 2025). Although these effects are less common when ketamine is co-administered with propofol or benzodiazepines, they remain a pertinent consideration during sedation planning (Natoli, 2021).

Intravenous lidocaine, when used as an adjunct, may enhance sedation quality through several mechanisms, including modulation of nociceptive pathways, inhibition of spinal and peripheral nerve conduction, and blunting of airway reflexes (Fernández-Martínez et al., 2025; Yang et al., 2020). These effects may account for the consistently favourable performance of lidocaine-based regimens, especially in scenarios requiring deep sedation without compromising respiratory stability (Barbosa et al., 2025). In contrast, dexmedetomidine-containing regimens (e.g., dexmedetomidine-midazolam-propofol, dexmedetomidine-propofol-opioid) were associated with prolonged recovery times, likely attributable to the α2-adrenergic agonist’s context-sensitive half-life and residual sedative effects (Yu et al., 2022). Although dexmedetomidine offers analgesia and anxiolysis with minimal respiratory depression, its bradycardic and hypotensive effects–particularly when combined with other central depressants–may limit its applicability in procedures necessitating rapid turnover or hemodynamic stability is paramount (Verret et al., 2024; Sin et al., 2022).

In addition, remimazolam-opioid regimens also demonstrated a favourable safety profile, particularly in relation to hypotension and bradycardia (Barbosa et al., 2024). Remimazolam selectively targets GABA-A receptors and undergoes organ-independent metabolism via tissue esterases, enabling rapid and predictable recovery, even in elderly patients or those with hepatic or renal dysfunction (Lee and Shirley, 2021). When combined with opioids, remimazolam appears to maintain hemodynamic stability without compromising sedation depth, although further studies are needed to optimise dosing and evaluate its effects on patient satisfaction (Dong et al., 2023; Xiao et al., 2025).

Collectively, these mechanistic insights reinforce the notion that tailored pharmacological combinations may represent optimal sedation strategies for high-risk or prolonged gastrointestinal interventions. The integration of agents with distinct receptor targets, metabolism pathways, and side effect profiles facilitates the development of individualised regimens that align with both procedural and patient-specific demands. Such mechanistic understanding may support the design of more precise, patient-tailored sedation strategies in therapeutic gastrointestinal endoscopy.

### 4.4 Clinical implications and practical relevance

Our findings carry important implications for sedation management in advanced gastrointestinal endoscopy. While propofol-opioid combinations remain the most widely used and were reaffirmed as the reference regimen in our analysis, several alternative strategies demonstrated comparable or superior profiles depending on specific safety or recovery priorities. In particular, ketamine-propofol showed consistently favourable performance across all cardiorespiratory outcomes, making it a strong candidate for patients undergoing prolonged or hemodynamically challenging procedures such as ERCP and ESD. Likewise, lidocaine-based combinations (e.g., lidocaine-midazolam-propofol, lidocaine-propofol-opioid) ranked highly in both efficacy and safety domains, and may be considered as valuable adjuncts for enhancing sedation depth and reduce airway reactivity. These regimens, when applied within monitored anesthesia care (MAC) frameworks, offer the potential to deliver deep yet physiologically stable sedation without necessitating airway instrumentation or general anesthesia.

The favourable cardiovascular profiles observed with remimazolam-opioid regimens, particularly their association with reduced hypotension and bradycardia, support their potential utility in elderly patients or those with underlying cardiovascular comorbidities. However, these benefits must be weighed against variable effects on induction time and patient satisfaction, which remain less well defined and warrant further investigation. Conversely, dexmedetomidine-based regimens–while effective in enhancing satisfaction and analgesia–were consistently associated with delayed recovery and an increased risk of bradycardia. These regimens may be more appropriate in procedural contexts where prolonged sedation is acceptable or desirable, such as in complex interventions requiring post-procedural observation or in low-turnover settings where rapid recovery is not prioritised.

These differentiated benefit-risk profiles underscore the importance of individualised sedation strategies that are tailored to patient characteristics, procedural complexity, and institutional capabilities. By providing a structured, comparative assessment of sedation regimens, our findings support informed, evidence-based selection of pharmacological strategies tailored to the demands of therapeutic endoscopy. Future research may explore the development of algorithm-assisted sedation planning tools for sedation planning, incorporating factors such as procedural risk, recovery goals, and comorbidity profiles to optimise regimen selection in a patient- centred manner.

### 4.5 Strengths and limitations

This study represents the most comprehensive synthesis to date of pharmacological sedation strategies for therapeutic gastrointestinal endoscopy, incorporating 60 RCTs and over 7,000 patients. By focusing exclusively on advanced procedures such as ERCP and ESD, we addressed a critical evidence gap left by previous network meta-analyses, which primarily examined diagnostic or short-duration interventions. Our methodological rigor–encompassing prespecified outcome definitions, duplicate data extraction, RoB 2.0 – based risk of bias assessments, and GRADE certainty evaluations adapted for network meta-analysis–ensured a transparent and reliable appraisal of the evidence. In addition, the inclusion of cluster ranking and meta-regression analyses allowed for a multidimensional evaluation of benefit-risk profiles, enhancing the clinical interpretability of our findings across diverse procedural settings and patient populations.

Nevertheless, several limitations should be acknowledged. First, although outcome definitions were extracted and summarised as reported in the original studies, substantial heterogeneity remained in the definitions of key adverse events across trials. For example, hypotension was variably defined as an absolute systolic blood pressure threshold, a relative decrease from baseline, or changes in mean arterial pressure, while hypoxia was reported using different oxygen saturation cut-offs and bradycardia using different heart rate thresholds. Pooling these outcomes within single network nodes may have introduced important clinical heterogeneity and may have affected the magnitude, comparability, and ranking of treatment effects. In addition, procedural interference events were reported using heterogeneous definitions across trials and were treated as a composite outcome in this analysis, which may mask potential differences between specific types of procedural interruptions. Second, network consistency could not be confirmed for several outcomes, including induction time and satisfaction measures, precluding their inclusion in the network synthesis and limiting the scope of comparative inferences. Third, although we excluded studies involving general anesthesia or neuromuscular blockade, heterogeneity may still have arisen from differences in anesthesiologist involvement, sedation protocols, and monitoring standards. Fourth, opioid agents were grouped as a single class despite pharmacokinetic and pharmacodynamic differences among fentanyl, sufentanil, remifentanil, and other agents. While this simplified classification was necessary to ensure network connectivity, it may obscure regimen-specific effects on cardiorespiratory parameters and recovery trajectories. Finally, many treatment comparisons were informed by sparse direct evidence or yielded imprecise estimates, resulting in low or very low certainty ratings for a substantial proportion of network estimates. Future studies should prioritise the standardisation of outcome definitions and the inclusion of clinically meaningful endpoints relevant to procedural sedation. Well-powered, head-to-head trials comparing promising regimens–such as ketamine-propofol or remimazolam-opioid–are warranted to generate more robust and generalisable evidence. As the demand for complex endoscopic interventions continues to grow, optimising sedation strategies will remain a key component of enhancing patient safety and procedural success.

## 5 Conclusion

In conclusion, this network meta-analysis provides the most comprehensive and up-to-date synthesis of sedation strategies for complex therapeutic gastrointestinal endoscopic procedures such as ERCP and ESD. While propofol-opioid combinations remain a widely accepted reference regimen, alternative strategies–including ketamine-propofol, lidocaine-midazolam-propofol, and remimazolam-opioid–demonstrated favourable performance across multiple domains, particularly in terms of cardiorespiratory stability and recovery efficiency. These findings support a more individualised approach to sedation management, enabling clinicians to tailor pharmacologic regimens according to patient comorbidities, procedural demands, and institutional practices.

## Data Availability

The original contributions presented in the study are included in the article/Supplementary Material, further inquiries can be directed to the corresponding author.
