# Supplementary material for: Pharmacological sedation strategies for therapeutic gastrointestinal endoscopy: a systematic review and network meta-analysis of randomised controlled trials
Source: Front Pharmacol. 2026 Apr 21;17:1740424. doi: 10.3389/fphar.2026.1740424 (PMC13138993; doi:10.3389/fphar.2026.1740424)
Supplement: Supplementary file 2 [file Table1.docx]

**Table 1 Search strategy**

| **Step** | **Search strategy** **in PubMed** |
| --- | --- |
| #1 | **1. ((((((((((((((Hypnotics and Sedatives[MeSH Terms]) OR (Anesthetics, Intravenous[MeSH Terms])) OR (Propofol[MeSH Terms])) OR (Benzodiazepines[MeSH Terms])) OR (Midazolam[MeSH Terms])) OR (Dexmedetomidine[MeSH Terms])) OR (Etomidate[MeSH Terms])) OR (Remimazolam[Supplementary Concept])) OR (Propofol[Title/Abstract])) OR (Benzodiazepines[Title/Abstract])) OR (Remimazolam[Title/Abstract])) OR (Midazolam[Title/Abstract])) OR (Dexmedetomidine[Title/Abstract])) OR (Etomidate[Title/Abstract])) OR (Ciprofol[Title/Abstract]) (147344)** |
| #2 | **2. (((((((((((((((((((((((Anesthesia and Analgesia[MeSH Terms]) OR (Analgesics, Opioid[MeSH Terms])) OR (Fentanyl[MeSH Terms])) OR (Sufentanil[MeSH Terms])) OR (Remifentanil[MeSH Terms])) OR (Alfentanil[MeSH Terms])) OR (Meperidine[MeSH Terms])) OR (Hydromorphone[MeSH Terms])) OR (Nalbuphine[MeSH Terms])) OR (Butorphanol[MeSH Terms])) OR (Buprenorphine[MeSH Terms])) OR (Oxycodone[MeSH Terms])) OR (Dezocine[Supplementary Concept])) OR (Fentanyl[Title/Abstract])) OR (Sufentanil[Title/Abstract])) OR (Remifentanil[Title/Abstract])) OR (Alfentanil[Title/Abstract])) OR (Meperidine[Title/Abstract])) OR (Hydromorphone[Title/Abstract])) OR (Nalbuphine[Title/Abstract])) OR (Butorphanol[Title/Abstract])) OR (Buprenorphine[Title/Abstract])) OR (Oxycodone[Title/Abstract])) OR (Dezocine[Title/Abstract]) (342705)** |
| #3 | **3. (((((((((Anesthetics, General[MeSH Terms]) OR (Esketamine[Supplementary Concept])) OR (Ketamine[MeSH Terms])) OR (Tramadol[MeSH Terms])) OR (Lidocaine[MeSH Terms])) OR (Esketamine[Title/Abstract])) OR (Ketamine[Title/Abstract])) OR (Tramadol[Title/Abstract])) OR (Lidocaine[Title/Abstract])) OR (Ketofol[Title/Abstract]) (94785)** |
| #4 | **4. (((((((((((((((((((((((Endoscopic Mucosal Resection[MeSH Terms]) OR (Ultrasonography, Endoscopic[MeSH Terms])) OR (Endoscopic Mucosal Resection[Title/Abstract])) OR (Endoscopic Submucosal Dissections[Title/Abstract])) OR (Endoscopic Full Thickness Resection[Title/Abstract])) OR (Submucosal Tunneling Endoscopic Resection[Title/Abstract])) OR (Endoscopic Mucous Membrane Resection[Title/Abstract])) OR (Strip Biopsy[Title/Abstract])) OR (Endoscopic Submucosal Excavation[Title/Abstract])) OR (Per-Oral Endoscopic Myotomy[Title/Abstract])) OR (Percutaneous Endoscopic Gastrostomy[Title/Abstract])) OR (Endoscopic ultrasonography[Title/Abstract])) OR (Endoscopic Ultrasound-guided Gastrojejunostomy[Title/Abstract])) OR (Cholangiopancreatography, Endoscopic Retrograde[MeSH Terms])) OR (Endoscopic retrograde cholangiopancreatography[Title/Abstract])) OR (Endoscopic Retrograde Cholangiopancreatographies[Title/Abstract])) OR (EMR[Title/Abstract])) OR (ESD[Title/Abstract])) OR (EFTR[Title/Abstract])) OR (STER[Title/Abstract])) OR (POEM[Title/Abstract])) OR (EUS[Title/Abstract])) OR (EUS-GJ[Title/Abstract])) OR (ERCP[Title/Abstract]) (77197)** |
| #5 | **5. ((((Random*) OR (Random Allocation[MeSH Terms])) OR (Randomized controlled trials[MeSH Terms])) OR (Randomized Controlled Trials as Topic)) OR (Randomized controlled trial[Publication Type]) (1866079)** |
| #6 | **6. (1 OR 2 OR 3) AND 4 AND 5 (310)** |
| **Step** | **Search strategy in EMBASE** |
| #1 | 'hypnotic sedative agent'/exp **(585464)** |
| #2 | 'intravenous anesthetic agent'/exp **(3438)** |
| #3 | 'propofol'/exp OR 'propofol' **(83222)** |
| #4 | 'benzodiazepine derivative'/exp OR 'benzodiazepine derivative' **(359707)** |
| #5 | 'remimazolam'/exp OR 'remimazolam' **(1057)** |
| #6 | 'midazolam'/exp OR 'midazolam' **(70273)** |
| #7 | 'dexmedetomidine'/exp OR 'dexmedetomidine' **(23915)** |
| #8 | 'etomidate'/exp OR etomidate **(10513)** |
| #9 | 'ciprofol'/exp OR ciprofol **(160)** |
| #10 | #1–#9/OR **(747955)** |
| #11 | 'anesthesiological procedure'/exp OR 'anesthesiological procedure' **(1097615)** |
| #12 | 'narcotic analgesic agent'/exp OR 'narcotic analgesic agent' **(451256)** |
| #13 | 'fentanyl derivative'/exp OR 'fentanyl derivative' **(115882)** |
| #14 | 'sufentanil'/exp OR 'sufentanil' **(14584)** |
| #15 | 'remifentanil'/exp OR 'remifentanil' **(21700)** |
| #16 | 'alfentanil'/exp OR 'alfentanil' **(7641)** |
| #17 | 'pethidine'/exp OR 'pethidine' **(26998)** |
| #18 | 'hydromorphone'/exp OR 'hydromorphone' **(14879)** |
| #19 | 'nalbuphine'/exp OR 'nalbuphine' **(4171)** |
| #20 | 'butorphanol'/exp OR 'butorphanol' **(7723)** |
| #21 | 'buprenorphine'/exp OR 'buprenorphine' **(32657)** |
| #22 | 'oxycodone'/exp OR 'oxycodone' **(27808)** |
| #23 | 'dezocine'/exp OR 'dezocine' **(708)** |
| #24 | #11–#23/OR **(1374959)** |
| #25 | 'anesthetic agent'/exp OR 'anesthetic agent' **(780220)** |
| #26 | 'esketamine'/exp OR 'esketamine' **(2742)** |
| #27 | 'ketamine'/exp OR 'ketamine' **(76210)** |
| #28 | 'tramadol'/exp OR 'tramadol' **(33997)** |
| #29 | 'lidocaine'/exp OR 'lidocaine' **(106251)** |
| #30 | 'ketofol'/exp OR ketofol **(275)** |
| #31 | #25–#30/OR **(878677)** |
| #32 | 'endoscopic mucosal resection'/exp OR 'endoscopic mucosal resection' OR (endoscopic AND mucosal AND ('resection'/exp OR resection)) **(26022)** |
| #33 | 'endoscopic ultrasonography'/exp OR 'endoscopic ultrasonography' **(45519)** |
| #34 | 'endoscopic submucosal dissection'/exp OR 'endoscopic submucosal dissection' OR (endoscopic AND submucosal AND ('dissection'/exp OR dissection)) **(18495)** |
| #35 | 'endoscopic full thickness resection'/exp OR 'endoscopic full thickness resection' OR (endoscopic AND full AND ('thickness'/exp OR thickness) AND ('resection'/exp OR resection)) **(3420)** |
| #36 | 'submucosal tunneling endoscopic resection'/exp OR 'submucosal tunneling endoscopic resection' OR (submucosal AND ('tunneling'/exp OR tunneling) AND endoscopic AND ('resection'/exp OR resection)) **(807)** |
| #37 | 'endoscopic mucous membrane resection' OR (endoscopic AND mucous AND ('membrane'/exp OR membrane) AND ('resection'/exp OR resection)) **(100)** |
| #38 | 'strip biopsy' OR (strip AND ('biopsy'/exp OR biopsy)) **(1199)** |
| #39 | 'endoscopic submucosal excavation'/exp OR 'endoscopic submucosal excavation' OR (endoscopic AND submucosal AND ('excavation'/exp OR excavation)) **(134)** |
| #40 | 'peroral endoscopic myotomy'/exp OR 'peroral endoscopic myotomy' OR (peroral AND endoscopic AND ('myotomy'/exp OR myotomy)) **(4374)** |
| #41 | 'percutaneous endoscopic gastrostomy'/exp OR 'percutaneous endoscopic gastrostomy' **(10738)** |
| #42 | 'endoscopic ultrasound-guided gastrojejunostomy' OR (endoscopic AND 'ultrasound guided' AND ('gastrojejunostomy'/exp OR gastrojejunostomy)) **(315)** |
| #43 | 'endoscopic retrograde cholangiopancreatography'/exp OR 'endoscopic retrograde cholangiopancreatography' **(55589)** |
| #44 | (#32–#43/OR) AND (#10 OR #24 OR #31) **(2380142)** |
| #45 | #44 AND [randomized controlled trial]/lim AND [humans]/lim AND [embase]/lim **(570)** |
| **Step** | **Search strategy** **in CENTRAL** |
| #1 | MeSH descriptor: [Hypnotics and Sedatives] explode all trees **(4850)** |
| #2 | MeSH descriptor: [Anesthetics, Intravenous] explode all trees **(4245)** |
| #3 | MeSH descriptor: [Propofol] explode all trees **(6250)** |
| #4 | MeSH descriptor: [Benzodiazepines] explode all trees **(11686)** |
| #5 | (Remimazolam):ti,ab,kw **(1149)** |
| #6 | MeSH descriptor: [Midazolam] explode all trees **(3695)** |
| #7 | MeSH descriptor: [Dexmedetomidine] explode all trees **(3088)** |
| #8 | MeSH descriptor: [Etomidate] explode all trees **(465)** |
| #9 | (Ciprofol):ti,ab,kw **(312)** |
| #10 | MeSH descriptor: [Anesthesia and Analgesia] explode all trees **(34941)** |
| #11 | MeSH descriptor: [Analgesics, Opioid] explode all trees **(10666)** |
| #12 | MeSH descriptor: [Fentanyl] explode all trees **(6719)** |
| #13 | MeSH descriptor: [Sufentanil] explode all trees **(1261)** |
| #14 | MeSH descriptor: [Remifentanil] explode all trees **(2154)** |
| #15 | MeSH descriptor: [Alfentanil] explode all trees **(778)** |
| #16 | MeSH descriptor: [Meperidine] explode all trees **(1277)** |
| #17 | MeSH descriptor: [Hydromorphone] explode all trees **(497)** |
| #18 | MeSH descriptor: [Nalbuphine] explode all trees **(359)** |
| #19 | MeSH descriptor: [Butorphanol] explode all trees **(220)** |
| #20 | MeSH descriptor: [Buprenorphine] explode all trees **(1584)** |
| #21 | MeSH descriptor: [Oxycodone] explode all trees **(1206)** |
| #22 | (Dezocine):ti,ab,kw **(337)** |
| #23 | MeSH descriptor: [Anesthetics, General] explode all trees **(6231)** |
| #24 | (Esketamine):ti,ab,kw **(1494)** |
| #25 | MeSH descriptor: [Ketamine] explode all trees **(3186)** |
| #26 | MeSH descriptor: [Tramadol] explode all trees **(1413)** |
| #27 | MeSH descriptor: [Lidocaine] explode all trees **(7432)** |
| #28 | (Ketofol):ti,ab,kw **(288)** |
| #29 | #1–#28/OR **(68020)** |
| #30 | MeSH descriptor: [Endoscopic Mucosal Resection] explode all trees **(230)** |
| #31 | MeSH descriptor: [Endosonography] explode all trees **(560)** |
| #32 | (Endoscopic Submucosal Dissection):ti,ab,kw **(1166)** |
| #33 | (Endoscopic Full Thickness Resection):ti,ab,kw **(48)** |
| #34 | (Submucosal Tunneling Endoscopic Resection):ti,ab,kw **(18)** |
| #35 | (Endoscopic Mucous Membrane Resection):ti,ab,kw **(15)** |
| #36 | (Strip Biopsy):ti,ab,kw **(105)** |
| #37 | (Endoscopic Submucosal Excavation):ti,ab,kw **(6)** |
| #38 | (PerOral Endoscopic Myotomy):ti,ab,kw **(189)** |
| #39 | (Percutaneous Endoscopic Gastrostomy):ti,ab,kw **(370)** |
| #40 | MeSH descriptor: [Cholangiopancreatography, Endoscopic Retrograde] explode all trees **(964)** |
| #41 | #30–#40/OR **(3401)** |
| #42 | #29 AND #41 (Filters: Trails) **(216)** |

**Table 2 Definitions for all outcome measures**

| **Study (Author Year)** | **Definition of Procedural Interference Events** |
| --- | --- |
| Amir 2024 | Vomiting during the procedure |
| Angsuwatcharakon 2012 | NA |
| Aqeel 2024 | NA |
| Ashikari 2021 | Restlessness during the procedure |
| Ates 2021 | Oropharyngeal reflex during the procedure |
| Babu 2024 | NA |
| Bahrami 2016 | NA |
| Chen 2022 | NA |
| Chun 2012 | NA |
| Dhingra 2023 | Movement during the procedure |
| Ding 2024 | NA |
| Dong 2023 | Nausea during the procedure |
| Eberl 2020 | NA |
| Fabbri 2012 | NA |
| Gao 2025 | NA |
| Garg 2019 | NA |
| Goyal 2016 | NA |
| Han 2017 | NA |
| Han 2019 | Felt pain during the procedure |
| Hasanein 2013 | Movement during the procedure |
| Haytural 2015 | NA |
| Heidari 2014 | Retching during the procedure |
| Jung 2000 | NA |
| Kilic 2011 | Gagging during the procedure |
| Kim 2015 | NA |
| Kim 2016 | Movement during the procedure |
| Koruk 2020 | Nausea-vomiting during the procedure |
| Lee 2011 | NA |
| Lee 2012 | NA |
| Lee 2014 | Agitation or intolerance during the procedure |
| Lee 2023 | NA |
| Lee SJ 2015 | NA |
| Lee SP 2015 | Interfering events during the procedure |
| Liu 2020 | Movement during the procedure |
| Lu 2018 | Movement during the procedure |
| Mukhopadhyay 2015 | NA |
| Muller 2008 | NA |
| Narayanan 2015 | NA |
| Nonaka 2018 | Restlessness during the procedure |
| Pushkarna 2019 | Gagging during the procedure |
| Ramkiran 2015 | NA |
| Riphaus 2005 | NA |
| Sasaki 2012 | Manual restraint required during the procedure |
| Sethi 2014 | Gagging during the procedure |
| Shin 2017 | Movement during the procedure |
| Singh 2022 | NA |
| Singh 2023 | Nausea during the procedure |
| Soliman 2024 | NA |
| Srivastava 2018 | Gagging during the procedure |
| Srivastava 2021 | Restlessness during the procedure |
| Sun 2024 | Nausea-vomiting during the procedure |
| Takimoto 2011 | Restlessness during the procedure |
| Tian 2024 | NA |
| Wang 2024 | Movement during the procedure |
| Wehrmann 1999 | NA |
| Xiao 2024 | NA |
| Xin 2024 | Movement during the procedure |
| Yüksel 2007 | Nausea during the procedure |
| Zhang 2024 | NA |
| Zhou 2024 | Movement during the procedure |
| **Study (Author, Year)** | **Definition of Induction time** |
| Amir 2024 | NA |
| Angsuwatcharakon 2012 | From sedation to scope intubation |
| Aqeel 2024 | From initiation of infusion to achieve an RSS score of 4 |
| Ashikari 2021 | NA |
| Ates 2021 | NA |
| Babu 2024 | NA |
| Bahrami 2016 | NA |
| Chen 2022 | NA |
| Chun 2012 | NA |
| Dhingra 2023 | From the start of induction to a BIS value of 50–60 |
| Ding 2024 | From the start of induction dose to the first MOAA/S ≤1 |
| Dong 2023 | NCR |
| Eberl 2020 | NA |
| Fabbri 2012 | NA |
| Gao 2025 | NA |
| Garg 2019 | NA |
| Goyal 2016 | NA |
| Han 2017 | NCR |
| Han 2019 | NA |
| Hasanein 2013 | NA |
| Haytural 2015 | NA |
| Heidari 2014 | NA |
| Jung 2000 | NA |
| Kilic 2011 | NA |
| Kim 2015 | NA |
| Kim 2016 | From propofol bolus injection to MOAA/ S score ≤ 3 |
| Koruk 2020 | NA |
| Lee 2011 | From sedation start to procedure start |
| Lee 2012 | From first injection to onset of effective sedation |
| Lee 2014 | NA |
| Lee 2023 | From first study medication to sedation |
| Lee SJ 2015 | From sedation start to procedure start |
| Lee SP 2015 | NA |
| Liu 2020 | From first injection to the insertion of the endoscope |
| Lu 2018 | From drug administration to achieving target sedation level |
| Mukhopadhyay 2015 | NA |
| Muller 2008 | NA |
| Narayanan 2015 | NA |
| Nonaka 2018 | NA |
| Pushkarna 2019 | From initiation of infusion to achieve an RSS score of 4 |
| Ramkiran 2015 | NA |
| Riphaus 2005 | From drug administration to insertion of the endoscope |
| Sasaki 2012 | NA |
| Sethi 2014 | From initiation of infusion to achieve an RSS score of 3–4 |
| Shin 2017 | NA |
| Singh 2022 | From initiation of infusion to achieve an RSS score of 3 |
| Singh 2023 | From initiation of infusion to achieve a BIS of 60–80 |
| Soliman 2024 | From drug administration to achieving target sedation level |
| Srivastava 2018 | NA |
| Srivastava 2021 | From initiation of infusion to achieve an RSS score of 3–4 |
| Sun 2024 | NA |
| Takimoto 2011 | NA |
| Tian 2024 | From the initial administration to achieve a MOAA/S ≤2 |
| Wang 2024 | NCR |
| Wehrmann 1999 | From drug administration to insertion of the endoscope |
| Xiao 2024 | NA |
| Xin 2024 | NA |
| Yüksel 2007 | NA |
| Zhang 2024 | From drug administration to achieving target sedation level |
| Zhou 2024 | From initiation of infusion to an RSS score ≥ 4 |
| **Study (Author, Year)** | **Definition of Hypoxia** |
| Amir 2024 | NCR |
| Angsuwatcharakon 2012 | SpO_2_ < 90% lasting for more than 10 seconds |
| Aqeel 2024 | NCR |
| Ashikari 2021 | SpO_2_ ≤ 94% |
| Ates 2021 | SpO_2_ < 90% |
| Babu 2024 | NA |
| Bahrami 2016 | NCR |
| Chen 2022 | SpO_2_ < 90% |
| Chun 2012 | SpO_2_ < 90% |
| Dhingra 2023 | SpO_2_ <92% lasting for more than 10 seconds |
| Ding 2024 | SpO_2_ <93% lasting for more than 15 seconds |
| Dong 2023 | SpO_2_ < 90% lasting for more than 10 seconds |
| Eberl 2020 | SpO_2_ < 90% |
| Fabbri 2012 | SpO_2_ < 92% |
| Gao 2025 | SpO_2_ < 90% |
| Garg 2019 | NCR |
| Goyal 2016 | SpO_2_ < 90% lasting for more than 10 seconds |
| Han 2017 | SpO_2_ < 90% |
| Han 2019 | SpO_2_ < 90% |
| Hasanein 2013 | SpO_2_ < 90% |
| Haytural 2015 | NA |
| Heidari 2014 | Need to supplemental oxygen by mask or intranasal |
| Jung 2000 | NA |
| Kilic 2011 | NA |
| Kim 2015 | SpO_2_ < 90% |
| Kim 2016 | SpO_2_ < 90% |
| Koruk 2020 | SpO_2_ < 92% |
| Lee 2011 | SpO_2_ < 90% |
| Lee 2012 | SpO_2_ < 90% |
| Lee 2014 | SpO_2_ < 90% |
| Lee 2023 | SpO_2_ < 90% |
| Lee SJ 2015 | SpO_2_ < 90% |
| Lee SP 2015 | SpO_2_ < 90% |
| Liu 2020 | SpO_2_ < 90% lasting for more than 10 seconds |
| Lu 2018 | SpO_2_ < 90% |
| Mukhopadhyay 2015 | NA |
| Muller 2008 | NA |
| Narayanan 2015 | NA |
| Nonaka 2018 | SpO_2_ ≤ 94% |
| Pushkarna 2019 | SpO_2_ < 94% for >30 s treated with supplemental oxygen |
| Ramkiran 2015 | SpO_2_ < 90% |
| Riphaus 2005 | SpO_2_ < 90% |
| Sasaki 2012 | SpO_2_ ≤ 94% |
| Sethi 2014 | SpO_2_ < 92% lasting for more than 10 seconds |
| Shin 2017 | SpO_2_ < 90% lasting for more than 10 seconds |
| Singh 2022 | SpO_2_ < 92% lasting for more than 10 seconds |
| Singh 2023 | SpO_2_ < 90% |
| Soliman 2024 | SpO_2_ < 92% lasting for more than 10 seconds |
| Srivastava 2018 | NA |
| Srivastava 2021 | SpO_2_ < 92% lasting for more than 10 seconds |
| Sun 2024 | SpO_2_ < 95% or RR < 8 bpm for more than 1 minute |
| Takimoto 2011 | SpO_2_ ≤ 90% |
| Tian 2024 | NA |
| Wang 2024 | SpO_2_ < 90% lasting for more than 30 seconds |
| Wehrmann 1999 | SpO_2_ < 90% |
| Xiao 2024 | NA |
| Xin 2024 | SpO_2_ < 90% lasting for more than 5 seconds |
| Yüksel 2007 | SpO_2_ < 90% |
| Zhang 2024 | SpO_2_ < 90% lasting for more than 10 seconds |
| Zhou 2024 | SpO_2_ < 90% |
| **Study (Author, Year)** | **Definition of Hypotension** |
| Amir 2024 | NA |
| Angsuwatcharakon 2012 | SBP < 90 mmHg or decreases more than 25% from the baseline |
| Aqeel 2024 | NA |
| Ashikari 2021 | SBP ≤ 80 mmHg |
| Ates 2021 | NCR |
| Babu 2024 | NA |
| Bahrami 2016 | NA |
| Chen 2022 | MAP < 65 mmHg or decreases > 20% from the baseline |
| Chun 2012 | BP < 90/50 mmHg or decreases > 20% from the baseline |
| Dhingra 2023 | MAP < 60 mmHg or decreases > 20% from the baseline |
| Ding 2024 | SBP < 90 mmHg or a MAP fall of 20 % relative to the baseline and the duration exceeding 2 min |
| Dong 2023 | MAP < 65 mmHg or decreases > 20% from the baseline |
| Eberl 2020 | BP decreases > 20% from the baseline |
| Fabbri 2012 | NA |
| Gao 2025 | SBP < 90 mmHg |
| Garg 2019 | BP decreases > 20% from the baseline |
| Goyal 2016 | MAP < 70 mmHg |
| Han 2017 | SBP < 90 mmHg |
| Han 2019 | SBP < 90 mmHg |
| Hasanein 2013 | MAP decreases > 20% from the baseline |
| Haytural 2015 | NA |
| Heidari 2014 | NA |
| Jung 2000 | NCR |
| Kilic 2011 | MAP < 60 mmHg or decreases > 20% from the baseline |
| Kim 2015 | BP decreases > 20% from the baseline |
| Kim 2016 | NA |
| Koruk 2020 | MAP decreases > 20% from the baseline |
| Lee 2011 | SBP < 90 mmHg |
| Lee 2012 | SBP < 90 mmHg |
| Lee 2014 | SBP < 90 mmHg or decreases more than 20% from the baseline |
| Lee 2023 | SBP < 90 mmHg or decreases more than 20% from the baseline |
| Lee SJ 2015 | SBP < 90 mmHg |
| Lee SP 2015 | NCR |
| Liu 2020 | SBP < 90 mmHg |
| Lu 2018 | MAP < 70 mmHg |
| Mukhopadhyay 2015 | NA |
| Muller 2008 | MAP decreases > 20% from the baseline |
| Narayanan 2015 | NA |
| Nonaka 2018 | SBP ≤ 80 mmHg |
| Pushkarna 2019 | NA |
| Ramkiran 2015 | MAP decreases > 20% from the baseline |
| Riphaus 2005 | NCR |
| Sasaki 2012 | SBP ≤ 90 mmHg |
| Sethi 2014 | MAP < 60 mmHg or decreases > 20% from the baseline |
| Shin 2017 | MAP < 60 mmHg or decreases > 20% from the baseline |
| Singh 2022 | NA |
| Singh 2023 | SBP < 90 mmHg |
| Soliman 2024 | MAP < 60 mmHg or decreases > 20% from the baseline |
| Srivastava 2018 | MAP decreases > 30% from the baseline |
| Srivastava 2021 | NA |
| Sun 2024 | BP < 90/50 mmHg or MAP decreases ≥ 20% from the baseline |
| Takimoto 2011 | BP decreases > 30% from the baseline |
| Tian 2024 | NA |
| Wang 2024 | NA |
| Wehrmann 1999 | SBP < 90 mmHg |
| Xiao 2024 | MAP <65 mmHg for at least 1 minute |
| Xin 2024 | MAP < 65 mmHg or decreases > 20% from the baseline |
| Yüksel 2007 | BP decreases > 20% from the baseline |
| Zhang 2024 | MAP <65 mmHg |
| Zhou 2024 | MAP < 65 mmHg or decreases > 30% from the baseline |
| **Study (Author, Year)** | **Definition of Bradycardia** |
| Amir 2024 | NA |
| Angsuwatcharakon 2012 | HR < 50 beats/min |
| Aqeel 2024 | NCR |
| Ashikari 2021 | HR ≤ 45 beats/min |
| Ates 2021 | NA |
| Babu 2024 | NA |
| Bahrami 2016 | NA |
| Chen 2022 | HR < 50 beats/min |
| Chun 2012 | NA |
| Dhingra 2023 | HR < 50 beats/min |
| Ding 2024 | NA |
| Dong 2023 | NCR |
| Eberl 2020 | HR decreases > 20% from the baseline |
| Fabbri 2012 | HR < 50 beats/min |
| Gao 2025 | NA |
| Garg 2019 | NA |
| Goyal 2016 | HR < 50 beats/min |
| Han 2017 | HR < 50 beats/min |
| Han 2019 | HR < 50 beats/min |
| Hasanein 2013 | HR < 55 beats/min |
| Haytural 2015 | NA |
| Heidari 2014 | NA |
| Jung 2000 | NCR |
| Kilic 2011 | HR < 50 beats/min or decreases > 20% from the baseline |
| Kim 2015 | HR decreases > 20% from the baseline |
| Kim 2016 | NA |
| Koruk 2020 | HR decreases > 20% from the baseline |
| Lee 2011 | HR < 50 beats/min |
| Lee 2012 | HR < 50 beats/min |
| Lee 2014 | HR < 50 beats/min or decreases > 25% from the baseline |
| Lee 2023 | HR < 60 beats/min or decreases > 25% from the baseline |
| Lee SJ 2015 | HR < 50 beats/min |
| Lee SP 2015 | HR < 45 beats/min or decreases > 25% from the baseline |
| Liu 2020 | HR < 50 beats/min |
| Lu 2018 | HR < 50 beats/min |
| Mukhopadhyay 2015 | NA |
| Muller 2008 | NA |
| Narayanan 2015 | NA |
| Nonaka 2018 | HR ≤ 45 beats/min |
| Pushkarna 2019 | NA |
| Ramkiran 2015 | HR < 50 beats/min |
| Riphaus 2005 | HR < 50 beats/min |
| Sasaki 2012 | HR ≤ 50 beats/min |
| Sethi 2014 | HR < 50 beats/min or decreases > 20% from the baseline |
| Shin 2017 | HR < 50 beats/min |
| Singh 2022 | NA |
| Singh 2023 | HR < 60 beats/min |
| Soliman 2024 | NA |
| Srivastava 2018 | NA |
| Srivastava 2021 | NA |
| Sun 2024 | NCR |
| Takimoto 2011 | NA |
| Tian 2024 | NA |
| Wang 2024 | NA |
| Wehrmann 1999 | HR < 50 beats/min |
| Xiao 2024 | HR < 50 beats/min |
| Xin 2024 | HR < 45 beats/min |
| Yüksel 2007 | NA |
| Zhang 2024 | HR < 45 beats/min |
| Zhou 2024 | HR < 50 beats/min |
| **Study (Author, Year)** | **Definition of Recovery Time** |
| Amir 2024 | NA |
| Angsuwatcharakon 2012 | From scope withdrawal to modified Aldrete score of 10 |
| Aqeel 2024 | From discontinuation of the infusion to achieving an MAS of 9 |
| Ashikari 2021 | NA |
| Ates 2021 | From the end of the procedure until Aldrete score of 8 |
| Babu 2024 | NA |
| Bahrami 2016 | From the end of the procedure until Aldrete score of 9 |
| Chen 2022 | NCR |
| Chun 2012 | From the cessation of the drug infusion to the recovery state |
| Dhingra 2023 | From scope withdrawal to modified Aldrete score of 10 |
| Ding 2024 | From the end of the procedure until Aldrete score of 9 |
| Dong 2023 | NCR |
| Eberl 2020 | From the end of the procedure to MOAA/S > 4 |
| Fabbri 2012 | From the end of drug infusion to achieving a PAR score of 9 |
| Gao 2025 | NCR |
| Garg 2019 | NA |
| Goyal 2016 | From scope withdrawal to modified Aldrete score of 9 |
| Han 2017 | From the end of the procedure until modified Aldrete score of 10 |
| Han 2019 | From procedure completion to reaching Aldrete score of 10 |
| Hasanein 2013 | From procedure completion to reaching Aldrete score of 9 |
| Haytural 2015 | NA |
| Heidari 2014 | Length of stay in recovery room |
| Jung 2000 | NA |
| Kilic 2011 | NA |
| Kim 2015 | NA |
| Kim 2016 | Length of stay in recovery room |
| Koruk 2020 | From procedure completion to reaching Aldrete score of 9 |
| Lee 2011 | From withdrawal of the endoscope to recovery |
| Lee 2012 | From procedure completion to reaching Aldrete score of 10 |
| Lee 2014 | From scope withdrawal to modified Aldrete score of 9 |
| Lee 2023 | From the end of the procedure to MOAA/S of 5 |
| Lee SJ 2015 | From procedure completion to reaching Aldrete score of 10 |
| Lee SP 2015 | From procedure completion to reaching Aldrete score of 9 |
| Liu 2020 | From scope withdrawal to full recovery |
| Lu 2018 | From the recovery unit to reaching a modified SRS of 6 |
| Mukhopadhyay 2015 | NCR |
| Muller 2008 | NA |
| Narayanan 2015 | NA |
| Nonaka 2018 | From the cessation of administration to the conscious state |
| Pushkarna 2019 | From the cessation of administration to modified Aldrete score of 9 |
| Ramkiran 2015 | From the end of the procedure until modified Aldrete score of 10 |
| Riphaus 2005 | NCR |
| Sasaki 2012 | NA |
| Sethi 2014 | NA |
| Shin 2017 | From the end of the procedure until modified Aldrete score of 10 |
| Singh 2022 | From the end of the procedure until modified Aldrete score of 9 |
| Singh 2023 | From the cessation of administration to modified Aldrete score of 9 |
| Soliman 2024 | From procedure completion to reaching Aldrete score of 9 |
| Srivastava 2018 | From procedure completion to reaching Aldrete score of 9 |
| Srivastava 2021 | NA |
| Sun 2024 | Length of stay in recovery room |
| Takimoto 2011 | NA |
| Tian 2024 | Length of stay in recovery room |
| Wang 2024 | NCR |
| Wehrmann 1999 | NCR |
| Xiao 2024 | From procedure completion to state date of birth |
| Xin 2024 | From the end of the procedure until modified Aldrete score of 9 |
| Yüksel 2007 | From procedure completion to reaching Aldrete score of 9 |
| Zhang 2024 | From the end of the procedure until modified Aldrete score of 9 |
| Zhou 2024 | Length of stay in recovery room |

Abbreviations: NA - not available; NCR - not comprehensively reported; BIS - bispectral index; MOAA/S - modified observer assessment of the alert/sedation; RSS - Ramsay Sedation Scale; RR - respiratory rate; BP - blood pressure; DBP - diastolic blood pressure; MAP - mean arterial pressure; SBP - systolic blood pressure; HR - heart rate; MAS - modified Aldrete score; PAR - post-anaesthetic recovery score; SRS - Steward Recovery Score

**Table 3 Studies included after full-text assessment**

| No. | Included Study |
| --- | --- |
| 1 | Amir, M., Nawaz, N., and Aijaz, M. (2024). Comparative Evaluation of Dexmedetomidine versus Propofol for Sedation in Endoscopic Retrograde Cholangiopancreatography (ERCP) Patients with Impaired Liver Function. *Int J Med Pub Health* 14: 306–11. doi: 10.70034/ijmedph.2024.4.59 |
| 2 | Angsuwatcharakon, P., Rerknimitr, R., Ridtitid, W., Kongkam, P., Poonyathawon, S., Ponauthai, Y., et al. (2012). Cocktail sedation containing propofol versus conventional sedation for ERCP: a prospective, randomized controlled study. *BMC Anesthesiol* 12: 20. doi: 10.1186/1471-2253-12-20 |
| 3 | Aqeel, N., Abbas, M. Q., Abbas, S. M., Farooq, M. F., Siddique, M., and Malik, S. (2024). Dexmedetomidine versus Ketofol for Moderate Sedation in Endoscopic Retrograde Cholangiopancreatography. *J Coll Physicians Surg Pak* 34: 1019–23. doi: 10.29271/jcpsp.2024.09.1019 |
| 4 | Ashikari, K., Nonaka, T., Higurashi, T., Takatsu, T., Yoshihara, T., Misawa, N., et al. (2021). Efficacy of sedation with dexmedetomidine plus propofol during esophageal endoscopic submucosal dissection. *J Gastroenterol Hepatol* 36: 1920–6. doi: 10.1111/jgh.15417 |
| 5 | Ates, I., Aydin, M. E., Albayrak, B., Disci, E., Ahiskalioglu, E. O., Celik, E. C., et al. (2021). Pre-procedure intravenous lidocaine administration on propofol consumption for endoscopic retrograde cholangiopancreatography: A prospective, randomized, double-blind study. *J Gastroenterol Hepatol* 36: 1286–1290. doi: 10.1111/jgh.15356 |
| 6 | Babu, T. R., Kumar, M. R. A., Anup, N. R., and Shetty, S. M. (2024). Dexmedetomidine as an Adjunct to Propofol in Patients Undergoing Elective Endoscopic Retrograde Cholangio-Pancreaticography - A Double-blind Randomized Controlled Study. *J Pharm Bioallied Sci* 16: S399–S402. doi: 10.4103/jpbs.jpbs_617_23 |
| 7 | Bahrami, Gorji, F., Amri, P., Shokri, J., Alereza, H., and Bijani, A. (2016). Sedative and Analgesic Effects of Propofol-Fentanyl Versus Propofol-Ketamine During Endoscopic Retrograde Cholangiopancreatography: A Double-Blind Randomized Clinical Trial. *Anesth Pain Med* 6: e39835. doi: 10.5812/aapm.39835 |
| 8 | Chen, M., Sun, Y., Li, X., Zhang, C., Huang, X., Xu, Y., et al. (2022). Effectiveness of single loading dose of dexmedetomidine combined with propofol for deep sedation of endoscopic retrograde cholangiopancreatography (ERCP) in elderly patients: a prospective randomized study. *BMC Anesthesiol* 22: 85. doi: 10.1186/s12871-022-01630-8 |
| 9 | Chun, S. Y., Kim, K. O., Park, D. S., Kim, S. Y., Park, J. W., Baek, I. H., et al. (2012). Safety and efficacy of deep sedation with propofol alone or combined with midazolam administrated by nonanesthesiologist for gastric endoscopic submucosal dissection. *Gut Liver* 6: 464–70. doi: 10.5009/gnl.2012.6.4.464 |
| 10 | Dhingra, U., Mantri, N., Pani, S., Tempe, D. K., and Arora, M. (2023). Etomidate Versus Propofol for Monitored Anesthesia Care During Endoscopic Retrograde Cholangiopancreatography: A Prospective Randomized Controlled Trial. *Cureus* 15: e43178. doi: 10.7759/cureus.43178 |
| 11 | Ding, G., Wang, L., Zhao, W., Diao, Y., and Song, D. (2024). Comparison of the efficacy and safety of ciprofol and propofol for ERCP anesthesia in older patients: A single-center randomized controlled clinical study. *J Clin Anesth* 99: 111609. doi: 10.1016/j.jclinane.2024.111609 |
| 12 | Dong, SA, Guo, Y, Liu, SS, Wu, LL, Wu, LN, Song, K, et al. (2023). A randomized, controlled clinical trial comparing remimazolam to propofol when combined with alfentanil for sedation during ERCP procedures. *J Clin Anesth* 86: 111077. doi: 10.1016/j.jclinane.2023.111077 |
| 13 | Eberl, S., Koers, L., van, Hooft, J., de, Jong, E., Hermanides, J., Hollmann, M. W., et al. (2020). The effectiveness of a low-dose esketamine versus an alfentanil adjunct to propofol sedation during endoscopic retrograde cholangiopancreatography: A randomised controlled multicentre trial. *Eur J Anaesthesiol* 37: 394–401. doi: 10.1097/EJA.0000000000001134 |
| 14 | Fabbri, L. P., Nucera, M., Marsili, M., Al, Malyan, M., and Becchi, C. (2012). Ketamine, propofol and low dose remifentanil versus propofol and remifentanil for ERCP outside the operating room: is ketamine not only a "rescue drug"? *Med Sci Monit* 18: CR575–80. doi: 10.12659/msm.883354 |
| 15 | Gao, H., Yin, Z. Y., Hao, L. X., Wang, J., Cai, H. L., Guo, J., et al. (2025). Intravenous lidocaine decreased the incidence of SRAEs for ERCP procedures in elderly frailty patients, a randomized controlled trial. *Surg Endosc* 39: 1635–42. doi: 10.1007/s00464-024-11451-0 |
| 16 | Garg, A., Prasad, M. K., Jheetay, G. S., Varshney, R. K., Choudhary, A. K., and Garg, H. S. (2019). Comparison of propofol alone and propofol-ketamine mixture for sedation during endoscopic retrograde cholangiopancreatography (ERCP): A randomised study. *J Clin Diagn Res* 13: UC01–3. doi: 10.7860/JCDR/2019/40683.12768 |
| 17 | Goyal, R., Hasnain, S., Mittal, S., and Shreevastava, S. (2016). A randomized, controlled trial to compare the efficacy and safety profile of a dexmedetomidine-ketamine combination with a propofol-fentanyl combination for ERCP. *Gastrointest Endosc* 83: 928–33. doi: 10.1016/j.gie.2015.08.077 |
| 18 | Han, S. J., Lee, T. H., Park, S. H., Cho, Y. S., Lee, Y. N., Jung, Y., et al. (2017). Efficacy of midazolam- versus propofol-based sedations by non-anesthesiologists during therapeutic endoscopic retrograde cholangiopancreatography in patients aged over 80 years. *Dig Endosc* 29: 369–76. doi: 10.1111/den.12841 |
| 19 | Han, S. J., Lee, T. H., Yang, J. K., Cho, Y. S., Jung, Y., Chung, I. K., et al. (2019). Etomidate Sedation for Advanced Endoscopic Procedures. *Dig Dis Sci* 64: 144–51. doi: 10.1007/s10620-018-5220-3 |
| 20 | Hasanein, R., and El-Sayed, W. (2013). Ketamine/propofol versus fentanyl/propofol for sedating obese patients undergoing endoscopic retrograde cholangiopancreatography (ERCP). *Egypt J Anaesth* 29: 207–11. doi: 10.1016/j.egja.2013.02.009 |
| 21 | Haytural, C., Aydınlı, B., Demir, B., Bozkurt, E., Parlak, E., Dişibeyaz, S., et al. (2015). Comparison of Propofol, Propofol-Remifentanil, and Propofol-Fentanyl Administrations with Each Other Used for the Sedation of Patients to Undergo ERCP. *Biomed Res Int* 2015: 465465. doi: 10.1155/2015/465465 |
| 22 | Heidari, S. M., and Loghmani, P. (2014). Assessment of the effects of ketamine-fentanyl combination versus propofol-remifentanil combination for sedation during endoscopic retrograde cholangiopancreatography. *J Res Med Sci* 19: 860–6 |
| 23 | Jung, M., Hofmann, C., Kiesslich, R., and Brackertz, A. (2000). Improved sedation in diagnostic and therapeutic ERCP: propofol is an alternative to midazolam. *Endoscopy* 32: 233–8. doi: 10.1055/s-2000-96 |
| 24 | Kilic, N., Sahin, S., Aksu, H., Yavascaoglu, B., Gurbet, A., Turker, G., et al. (2011). Conscious sedation for endoscopic retrograde cholangiopancreatography: dexmedetomidine versus midazolam. *Eurasian J Med* 43: 13–7. doi: 10.5152/eajm.2011.03 |
| 25 | Kim, J. E., Choi, J. B., Koo, B. N., Jeong, H. W., Lee, B. H., and Kim, S. Y. (2016). Efficacy of Intravenous Lidocaine During Endoscopic Submucosal Dissection for Gastric Neoplasm: A Randomized, Double-Blind, Controlled Study. *Medicine (Baltimore)* 95: e3593. doi: 10.1097/MD.0000000000003593 |
| 26 | Kim, N., Yoo, Y. C., Lee, S. K., Kim, H., Ju, H. M., and Min, K. T. (2015). Comparison of the efficacy and safety of sedation between dexmedetomidine-remifentanil and propofol-remifentanil during endoscopic submucosal dissection. *World J Gastroenterol* 21: 3671–8. doi: 10.3748/wjg.v21.i12.3671 |
| 27 | Koruk, S., Koruk, I., Arslan, A. M., Bilgi, M., Gul, R., and Bozgeyik, S. (2020). Dexmedetomidine or midazolam in combination with propofol for sedation in endoscopic retrograde cholangiopancreatography: a randomized double blind prospective study. *Wideochir Inne Tech Maloinwazyjne* 15: 526–32. doi: 10.5114/wiitm.2020.95066 |
| 28 | Lee, B. S., Ryu, J., Lee, S. H., Lee, M. G., Jang, S. E., Hwang, J. H., et al. (2014). Midazolam with meperidine and dexmedetomidine vs. midazolam with meperidine for sedation during ERCP: prospective, randomized, double-blinded trial. *Endoscopy* 46: 291–8. doi: 10.1055/s-0033-1358909 |
| 29 | Lee, C. K., Lee, S. H., Chung, I. K., Lee, T. H., Park, S. H., Kim, E. O., et al. (2011). Balanced propofol sedation for therapeutic GI endoscopic procedures: a prospective, randomized study. *Gastrointest Endosc* 73: 206–14. doi: 10.1016/j.gie.2010.09.035 |
| 30 | Lee, T. H., Lee, C. K., Park, S. H., Lee, S. H., Chung, I. K., Choi, H. J., et al. (2012). Balanced propofol sedation versus propofol monosedation in therapeutic pancreaticobiliary endoscopic procedures. *Dig Dis Sci* 57: 2113–21. doi: 10.1007/s10620-012-2234-0 |
| 31 | Lee, J., Jeong, S., Lee, D. H., and Park, J. S. (2023). Finding the ideal sedative: a non-inferiority study of remimazolam vs propofol in endoscopic retrograde cholangiopancreatography. *J Gastroenterol Hepatol* 38: 2160–6. doi: 10.1111/jgh.16354 |
| 32 | Lee, S. J., Lee, T. H., Park, S. H., Lee, Y. N., Jung, Y., Choi, H. J., et al. (2015). Efficacy of carbon dioxide versus air insufflation according to different sedation protocols during therapeutic endoscopic retrograde cholangiopancreatography: prospective, randomized, double-blind study. *Dig Endosc* 27: 512–21. doi: 10.1111/den.12448 |
| 33 | Lee, S. P., Sung, I. K., Kim, J. H., Lee, S. Y., Park, H. S., Shim, C. S., et al. (2015). Comparison of dexmedetomidine with on-demand midazolam versus midazolam alone for procedural sedation during endoscopic submucosal dissection of gastric tumor. *J Dig Dis* 16: 377–84. doi: 10.1111/1751-2980.12254 |
| 34 | Liu, J., Liu, X., Peng, L. P., Ji, R., Liu, C., and Li, Y. Q. (2020). Efficacy and safety of intravenous lidocaine in propofol-based sedation for ERCP procedures: a prospective, randomized, double-blinded, controlled trial. *Gastrointest Endosc* 92: 293–300. doi: 10.1016/j.gie.2020.02.050 |
| 35 | Lu, Z., Li, W., Chen, H., and Qian, Y. (2018). Efficacy of a Dexmedetomidine-Remifentanil Combination Compared with a Midazolam-Remifentanil Combination for Conscious Sedation During Therapeutic Endoscopic Retrograde Cholangio-Pancreatography: A Prospective, Randomized, Single-Blinded Preliminary Trial. *Dig Dis Sci* 63: 1633–40. doi: 10.1007/s10620-018-5034-3 |
| 36 | Mukhopadhyay, S., Niyogi, M., Sarkar, J., Mukhopadhyay, B. S., and Halder, S. K. (2015). The dexmedetomidine "augmented" sedato analgesic cocktail: An effective approach for sedation in prolonged endoscopic retrograde cholangio-pancreatography. *J Anaesthesiol Clin Pharmacol* 31: 201–6. doi: 10.4103/0970-9185.155149 |
| 37 | Muller, S., Borowics, S. M., Fortis, E. A., Stefani, L. C., Soares, G., Maguilnik, I., et al. (2008). Clinical efficacy of dexmedetomidine alone is less than propofol for conscious sedation during ERCP. *Gastrointest Endosc* 67: 651–9. doi: 10.1016/j.gie.2007.09.041 |
| 38 | Narayanan, S., Shannon, A., Nandalan, S., Jaitly, V., and Greer, S. (2015). Alternative sedation for the higher risk endoscopy: a randomized controlled trial of ketamine use in endoscopic retrograde cholangiopancreatography. *Scand J Gastroenterol* 50: 1293–303. doi: 10.3109/00365521.2015.1036113 |
| 39 | Nonaka, T., Inamori, M.., Miyashita, T, Inoh, Y., Kanoshima, K., Higurashi, T., et al. (2018). Can sedation using a combination of propofol and dexmedetomidine enhance the satisfaction of the endoscopist in endoscopic submucosal dissection? *Endosc Int Open* 6: E3–10. doi: 10.1055/s-0043-122228 |
| 40 | Pushkarna, G., Sarangal, P., Pushkarna, V., and Gupta, R. (2019). Comparative Evaluation of Dexmedetomidine versus Midazolam as Premedication to Propofol Anesthesia in Endoscopic Retrograde Cholangiopancreatography. *Anesth Essays Res* 13: 297–302. doi: 10.4103/aer.AER_62_19 |
| 41 | Ramkiran, S., Iyer, S. S., Dharmavaram, S., Mohan, C. V., Balekudru, A., and Kunnavil, R. (2015). BIS Targeted Propofol Sparing Effects of Dexmedetomidine Versus Ketamine in Outpatient ERCP: A Prospective Randomised Controlled Trial. *J Clin Diagn Res* 9: UC07–12. doi: 10.7860/JCDR/2015/12435.5991 |
| 42 | Riphaus, A., Stergiou, N., and Wehrmann, T. (2005). Sedation with propofol for routine ERCP in high-risk octogenarians: a randomized, controlled study. *Am J Gastroenterol* 100: 1957–63. doi: 10.1111/j.1572-0241.2005.41672.x |
| 43 | Sasaki, T., Tanabe, S., Azuma, M., Sato, A., Naruke, A., Ishido, K., et al. (2012). Propofol sedation with bispectral index monitoring is useful for endoscopic submucosal dissection: a randomized prospective phase II clinical trial. *Endoscopy* 44: 584–9. doi: 10.1055/s-0032-1306776 |
| 44 | Sethi, P., Mohammed, S., Bhatia, P. K., and Gupta, N. (2014). Dexmedetomidine versus midazolam for conscious sedation in endoscopic retrograde cholangiopancreatography: An open-label randomised controlled trial. *Indian J Anaesth* 58: 18–24. doi: 10.4103/0019-5049.126782 |
| 45 | Shin, S., Park, C. H., Kim, H. J., Park, S. H., Lee, S. K., Yoo, Y. C. (2017). Patient satisfaction after endoscopic submucosal dissection under propofol-based sedation: a small premedication makes all the difference. *Surg Endosc* 31: 2636–44. doi: 10.1007/s00464-016-5276-0 |
| 46 | Singh, A., Iyer, K. V., Maitra, S., Khanna, P., Sarkar, S., Ahuja, V., et al. (2022). Ketamine and dexmedetomidine (Keto-dex) or ketamine and propofol (Keto-fol) for procedural sedation during endoscopic retrograde cholangiopancreatography: Which is safer? A randomized clinical trial. *Indian J Gastroenterol* 41: 583–90. doi: 10.1007/s12664-022-01291-y |
| 47 | Singh, J., Pathania, J., Bodh, V., Sharma, R., Kumar, R., and Sharma, B. (2023). Etomidate-ketamine versus dexmedetomidine-ketamine for entropy-guided procedural sedation during endoscopic retrograde cholangiopancreatography procedures: A randomized single blind study. *Indian J Gastroenterol* 42: 177–84. doi: 10.1007/s12664-022-01326-4 |
| 48 | Soliman, A. M., Hamad, Y. M., Almaghraby, A. A., Mohamed, A. A., and Abdallah, S. R. (2024). Propofol versus Dexmedetomidine for Sedation of Cancer Patients Undergoing Endoscopic Retrograde Cholangiopancreatography: Randomized Single-Blinded Controlled Study. *Anesth Pain Med* 14: e148512. doi: 10.5812/aapm-148512 |
| 49 | Srivastava, N., Harjai, M., Kumar, S., Rai, S., Malviya, D., and Tripathi, M. (2021). A comparative study of dexmedetomidine and propofol infusion during monitored anesthesia care (MAC) in endoscopic retrograde cholangiopancreatography (ERCP): a randomized controlled trial. *Ain-Shams J Anesthesiol* 13: 48. doi: 10.1186/s42077-021-00168-0 |
| 50 | Srivastava, V., and Agrawal, S. (2018). Comparative Evaluation of Propofol Fentanyl, Propofol-Midazolam and Propofol-Dexmedetomidine on Haemodynamic and Postoperative Recovery for Endoscopic Retrograde Cholangiopancreatography. *J Clin Diagn Res* 12: UC01–5. doi: 10.7860/JCDR/2018/32201.11730 |
| 51 | Sun, Z., Shi, J., Liu, C., Zhang, J., Liu, Y., Wu, Y., et al. (2024). The Effect of Low-Dose Dexmedetomidine on Perioperative Neurocognitive Dysfunction in Elderly Patients Undergoing Endoscopic Retrograde Cholangiopancreatography (ERCP): A Randomized, Controlled, Double-Blind Trial. *Drug Des Devel Ther* 18: 3715–25. doi: 10.2147/DDDT.S470514 |
| 52 | Takimoto, K., Ueda, T., Shimamoto, F., Kojima, Y., Fujinaga, Y., Kashiwa, A., et al. (2011). Sedation with dexmedetomidine hydrochloride during endoscopic submucosal dissection of gastric cancer. *Dig Endosc* 23: 176–81. doi: 10.1111/j.1443-1661.2010.01080.x |
| 53 | Tian, Y. Q., Chen, D. K., Zhang, H. M., and Sun, Y. M. (2024). Comparative analysis of the therapeutic efficacy of remimazolam tosylate and propofol in older adults undergoing painless endoscopic retrograde cholangiopancreatography. *Front Pharmacol* 15: 1404536. doi: 10.3389/fphar.2024.1404536 |
| 54 | Wang, J., Wang, R., Ma, X., Zhu, W., Zhang, B., Ma, Y., et al. (2024). Comparative efficacy of ciprofol and propofol in reducing respiratory depression during ERCP anesthesia: a randomized controlled trial. *BMC Anesthesiol* 24: 404. doi: 10.1186/s12871-024-02791-4 |
| 55 | Wehrmann, T., Kokabpick, S., Lembcke, B., Caspary, W. F., and Seifert, H. (1999). Efficacy and safety of intravenous propofol sedation during routine ERCP: a prospective, controlled study. *Gastrointest Endosc* 49: 677–83. doi: 10.1016/s0016-5107(99)70281-6 |
| 56 | Xiao, Y. Y., Zou, H. D., Qin, X. N., Zhu, R., and Dai, R. P. (2025). A Comparison of Remimazolam versus Propofol on Blood Pressure Changes During Therapeutic Endoscopic Retrograde Cholangiopancreatography: A Randomized Controlled Trial. *Anesth Analg* 141: 90–9. doi: 10.1213/ANE.0000000000007309 |
| 57 | Xin, W. D., Cui, B., Zhaozhu, Z. Z., Diao, Y. G., and Song, D. D. (2024). [Comparison of the efficacy of remimazolam and propofol on hemodynamics and quality of early postoperative recovery in elderly patients with frailty undergoing endoscopic retrograde cholangiopancreatography]. *Zhonghua Yi Xue Za Zhi* 104: 2936–42. Chinese. doi: 10.3760/cma.j.cn112137-20240102-00014 |
| 58 | Yüksel, O., Parlak, E., Köklü, S., Ertugrul, I., Tunç, B., and Sahin, B. (2007). Conscious sedation during endoscopic retrograde cholangiopancreatography: midazolam or midazolam plus meperidine? *Eur J Gastroenterol Hepatol* 19: 1002–6. doi: 10.1097/MEG.0b013e3282cf5167 |
| 59 | Zhang, W., Wang, L., Zhu, N., Wu, W., and Liu, H. (2024). A prospective, randomized, single-blinded study comparing the efficacy and safety of dexmedetomidine and propofol for sedation during endoscopic retrograde cholangiopancreatography. *BMC Anesthesiol* 24: 191. doi: 10.1186/s12871-024-02572-z |
| 60 | Zhou, B., Li, S., Luo, A., and Zheng, H. (2024). The efficacy and safety of remimazolam tosilate compared with propofol for endoscopic retrograde cholangiopancreatography under monitored anesthesia care: A single-center randomized controlled clinical trial. *Heliyon* 10: e38349. doi: 10.1016/j.heliyon.2024.e38349 |

**Table 4 Studies excluded after full-text assessment**

| No. | Excluded Study | Reason for Exclusion |
| --- | --- | --- |
| 1 | Abdalla MW, El Shal SM, El Sombaty AI, Abdalla NM, Zeedan RB. Propofol dexmedetomidine versus propofol ketamine for anesthesia of endoscopic retrograde cholangiopancreatography (ERCP) (A randomized comparative study). *Egypt J Anaesth* 2015; 31: 97–105. | Use of atracurium and endotracheal intubation. |
| 2 | Agostoni M, Fanti L, Arcidiacono PG, et al. Midazolam and pethidine versus propofol and fentanyl patient controlled sedation/analgesia for upper gastrointestinal tract ultrasound endoscopy: a prospective randomized controlled trial. *Dig Liver Dis* 2007; 39: 1024–9. | Diagnostic upper gastrointestinal endoscopy performed; inconsistency in outcome reporting (unit of induction time reported as minutes in text and seconds in table). |
| 3 | Arun N, Kumar R. Dexmedetomidine versus midazolam for conscious sedation in endoscopic retrograde cholangiopancreatography: An open-label randomised controlled trial. *Indian J Anaesth* 2014; 58: 789. | Published as a comment; not an original research article. |
| 4 | Baik SJ, Yi SY, Jung HK, Kim SE. Safety of sedated therapeutic endoscopic retrograde cholangiopancreatography in patients older than 70 years old. *Ewha Med J* 2014; 37: 92–7. | Control group did not receive anesthesia. |
| 5 | Bonta PI, Kok MF, Bergman JJ, et al. Conscious sedation for EUS of the esophagus and stomach: a double-blind, randomized, controlled trial comparing midazolam with placebo. *Gastrointest Endosc* 2003; 57: 842–7. | Control group did not receive anesthesia. |
| 6 | Breazu CM, Alexa AL, Urs O, et al. Sedation for Endoscopic Retrograde Cholangiopancreatography in Elderly Patients - the Effect of Intravenous Lidocaine Infusion. A Randomised, Double-Blind, Placebo Controlled Trial. *J Gastrointestin Liver Dis* 2022; 31: 453–8. | Inconsistent baseline data: 41 patients in group L, but sex distribution reported as 20 males and 34 females. |
| 7 | Chen WX, Lin HJ, Zhang WF, et al. Sedation and safety of propofol for therapeutic endoscopic retrograde cholangiopancreatography. *Hepatobiliary Pancreat Dis Int* 2005; 4: 437–40. | Control group did not receive anesthesia. |
| 8 | Cho YS, Seo E, Han JH, et al. Comparison of midazolam alone versus midazolam plus propofol during endoscopic submucosal dissection. *Clin Endosc* 2011; 44: 22–6. | Article retracted. |
| 9 | Choe JW, Chung MJ, Park SW, et al. Safety and efficacy of remimazolam versus propofol during EUS: a multicenter randomized controlled study. *Gastrointest Endosc* 2024; 100: 183–91. e1. | Diagnostic upper EUS performed. |
| 10 | Demir M, Balkiz Soyal Ö, Aytaç BG. Assessment of Optic Nerve Sheath Diameter in Patients Undergoing Endoscopic Retrograde Cholangiopancreatography: A Prospective, Randomized, Controlled Double-Blinded Comparison of Propofol and Ketofol Anesthesia. *Niger J Clin Pract* 2024; 27: 22–8. | Relevant outcomes not reported. |
| 11 | Dewitt J, McGreevy K, Sherman S, Imperiale TF. Nurse-administered propofol sedation compared with midazolam and meperidine for EUS: a prospective, randomized trial. *Gastrointest Endosc* 2008; 68: 499–509. | Possible inclusion of diagnostic upper EUS. |
| 12 | el-Bitar N, Sfeir S. Evaluation of remifentanil in endoscopic retrograde cholangio-pancreatography. *Middle East J Anaesthesiol* 2006; 18: 1209–16. | Fentanyl vs. Remifentanil; both are opioids but not classifiable into separate intervention nodes. |
| 13 | Fanti L, Agostoni M, Arcidiacono PG, et al. Target-controlled infusion during monitored anesthesia care in patients undergoing EUS: propofol alone versus midazolam plus propofol. A prospective double-blind randomised controlled trial. *Dig Liver Dis* 2007; 39: 81–6. | Incorrect propofol TCI dosage reported (target plasma concentration stated as 4 mg/mL, which is clinically implausible) |
| 14 | Fassoulaki A, Iatrelli I, Vezakis A, Polydorou A. Deep sedation for endoscopic cholangiopancreatography with or without pre or intraprocedural opioids: A double-blind randomised controlled trial. *Eur J Anaesthesiol* 2015, 32: 602–8. | Intranasal administration. |
| 15 | Garg I, Hasnain S. A Comparative Study of Propofol-Dexmedetomidine Versus Propofol-Ketamine for the Anesthetic Management of Patients During Endoscopic Retrograde Cholangiopancreatography. *Cureus* 2024; 16: e74472. | General anesthesia with atracurium and endotracheal intubation. |
| 16 | Ghomeishi A, Akhondzadeh R, Baghbanian R, Mahmoudi K, Bakhtiari N. A Comparison of the Sedative Effect of Dexmedetomidine and Midazolam on Patients Undergoing Gastrointestinal Endosonography Outside the Operating Room. *Anesth Pain Med* 2023; 13: e109721. | Quasi-randomization based on odd/even medical record numbers |
| 17 | Güitrón-Cantú A, Adalid-Martínez R, Gutiérrez-Bermúdez JA, Segura-López FK, García Vázquez A. [Does the use of fentanyl make Vater's ampulla cannulation difficult? A prospective and comparative study]. *Rev Gastroenterol Mex* 2010; 75: 142–8. | Full text unavailable. |
| 18 | Guo P, Wu H, Liu L, Zhao Q, Jin Z. Efficacy of an Oxycodone-Propofol Combination versus a Fentanyl-Propofol Combination in Conscious Sedation during Therapeutic Endoscopic Retrograde Cholangiopancreatography in Elderly Patients. *Gerontology* 2021; 67: 9–16. | Fentanyl vs. Oxycodone; both are opioids but not classifiable into separate intervention nodes. |
| 19 | Hannallah MS, David M, Carroll J, Haddad N, Charabaty A, Barton F. Comparison of propofol vs. Propofol/ remifentanil anesthesia in upper GI endoscopic ultrasound examination (EUS). *Ambul Surg* 2012; 18: 42–4. | Diagnostic upper EUS performed. |
| 20 | Hu J, Gu X, Zhu W, et al. Comparison of anesthetic effects of different doses of alfentanil combined with ciprofol in elderly patients undergoing ERCP: a randomized controlled trial. *BMC Anesthesiol* 2023; 23: 353. | Comparison of different doses of the same drug (alfentanil). |
| 21 | Javaid H, Riasat MI, Shah SJ, Hashmi M, Khurana S, Sultan F. Fentanyl-Propofol Versus Ketamine-Propofol Combination for Sedation and Recovery in ERCP: A Double-Blinded Randomized Clinical Trial. *Pak J Med Health Sci* 2023; 17: 558–61. | Implausible fentanyl dose reported (1 mg/kg), far exceeding clinically acceptable range. |
| 22 | Kim MG, Park SW, Kim JH, et al. Etomidate versus propofol sedation for complex upper endoscopic procedures: a prospective double-blinded randomized controlled trial. *Gastrointest Endosc* 2017; 86: 452–61. | Possible inclusion of diagnostic EUS. |
| 23 | Kinugasa H, Higashi R, Miyahara K, et al. Dexmedetomidine for conscious sedation with colorectal endoscopic submucosal dissection: a prospective double-blind randomized controlled study. *Clin Transl Gastroenterol* 2018; 9: 167. | One group received pethidine (meperidine) alone. |
| 24 | Kiriyama S, Gotoda T, Sano H, et al. Safe and effective sedation in endoscopic submucosal dissection for early gastric cancer: a randomized comparison between propofol continuous infusion and intermittent midazolam injection. *J Gastroenterol* 2010; 45: 831–7. | Quasi-randomization using odd–even hospital registration numbers. |
| 25 | Kongkam P, Rerknimitr R, Punyathavorn S, et al. Propofol infusion versus intermittent meperidine and midazolam injection for conscious sedation in ERCP. *J Gastrointestin Liver Dis* 2008; 17: 291–7. | Allocation performed after ERCP scheduling with alternate patient assignment. |
| 26 | Krugliak P, Ziff B, Rusabrov Y, Rosenthal A, Fich A, Gurman GM. Propofol versus midazolam for conscious sedation guided by processed EEG during endoscopic retrograde cholangiopancreatography: a prospective, randomized, double-blind study. *Endoscopy* 2000; 32: 677–82. | Quasi-randomization based on the last digit of patient ID. |
| 27 | Lin S, Wei Y, Zhuo Y, et al. Comparing Cognitive Recovery of Remimazolam versus Propofol in Elderly Patients Undergoing Colonoscopy: A Randomized Controlled Trial. *Clin Interv Aging* 2024; 19: 2133–43. | Diagnostic colonoscopy only. |
| 28 | Liu C, Wang Y, Yin Y, et al. Comparative Efficacy of Esketamine vs Sufentanil with Propofol for Sedation in EUS: A Randomized, Controlled Study. *J Pain Res* 2025; 18: 205–15. | Possible inclusion of diagnostic EUS. |
| 29 | Liu D, Gao X, Zhuo Y, et al. Effect of Esketamine on Cognitive Recovery After Propofol Sedation for Outpatient Colonoscopy: A Randomized Clinical Trial. *Drug Des Devel Ther* 2025; 19: 425–37. | Diagnostic colonoscopy only. |
| 30 | Luo X, Hou HJ, Chen PS, et al. Addition of Dexmedetomidine to the Anesthesia Regimen Attenuates Pain and Improves Early Recovery After Esophageal Endoscopic Submucosal Dissection: A Randomized Controlled Trial. *Drug Des Devel Ther* 2024; 18: 4551–62. | General anesthesia with rocuronium and endotracheal intubation. |
| 31 | Madácsy L, Bertalan V, Szepes A, Lonovics J. Effect of nalbuphine on the motility of the sphincter of Oddi in patients with suspected sphincter of Oddi dysfunction. *Gastrointest Endosc* 2003; 57: 319–23. | Relevant outcomes not reported. |
| 32 | Magni VC, Frost RA, Leung JW, Cotton PB. A randomized comparison of midazolam and diazepam for sedation in upper gastrointestinal endoscopy. *Br J Anaesth* 1983; 55: 1095–101. | Diazepam used for sedation; intervention not consistent with current clinical practice. |
| 33 | Mazanikov M, Udd M, Kylänpää L, et al. Patient-controlled sedation with propofol and remifentanil for ERCP: a randomized, controlled study. *Gastrointest Endosc* 2011; 73: 260–6. | Fentanyl vs. Remifentanil; both are opioids but not classifiable into separate intervention nodes. |
| 34 | Mazanikov M, Udd M, Kylänpää L, et al. Patient-controlled sedation for ERCP: a randomized double-blind comparison of alfentanil and remifentanil. *Endoscopy* 2012; 44: 487–92. | Alfentanil vs. Remifentanil; both are opioids but not classifiable into separate intervention nodes. |
| 35 | Netinatsunton N, Attasaranya S, Sottisuporn J, Witeerungrot T, Piratvisuth T, Ovartlarnporn B. Efficacy of propofol with midazolam and meperidine versus midazolam and meperidine for ERCP by endoscopists with variable experience. *J Gastroenterol Hepatol Res* 2014; 3: 1121–6. | Full text unavailable. |
| 36 | Nilsson A, Grossmann B, Kullman E, Uustal E, Sjöberg F, Nilsson L. Sedation during endoscopic retrograde cholangiopancreatography: a randomized controlled study of patient-controlled propofol sedation and that given by a nurse anesthetist. *Scand J Gastroenterol* 2015; 50: 1285–92. | Comparison focused on modes of drug administration (patient-controlled, nurse-controlled, operator-controlled) rather than drug type; not compatible with intervention grouping in this network meta-analysis. |
| 37 | Ominami M, Nagami Y, Shiba M, et al. Comparison of propofol with midazolam in endoscopic submucosal dissection for esophageal squamous cell carcinoma: a randomized controlled trial. *J Gastroenterol* 2018; 53: 397–406. | Full text unavailable. |
| 38 | Ong WC, Santosh D, Lakhtakia S, Reddy DN. A randomized controlled trial on use of propofol alone versus propofol with midazolam, ketamine, and pentazocine "sedato-analgesic cocktail" for sedation during ERCP. *Endoscopy* 2007; 39: 807–12. | Participants included individuals <18 years. |
| 39 | Park CH, Shin S, Lee SK, et al. Assessing the stability and safety of procedure during endoscopic submucosal dissection according to sedation methods: a randomized trial. *PLoS One* 2015; 10: e0120529. | Intramuscular administration used. |
| 40 | Park CH, Park SW, Hyun B, et al. Efficacy and safety of etomidate-based sedation compared with propofol-based sedation during ERCP in low-risk patients: a double-blind, randomized, noninferiority trial. *Gastrointest Endosc* 2018; 87: 174–84. | Intramuscular administration used. |
| 41 | Pun A, Dhungana A, Pariyar J, Upadhyay HP. Effectiveness of Fentanyl in Facilitating Cannulation of Ampulla of Vater during Endoscopic Retrograde Cholangiopancreatography. *J Nepal Health Res Counc* 2021; 18: 753–7. | Relevant outcomes not reported. |
| 42 | Qiu Y, Gu W, Zhao M, Zhang Y, Wu J. The hemodynamic stability of remimazolam compared with propofol in patients undergoing endoscopic submucosal dissection: A randomized trial. *Front Med (Lausanne)* 2022; 9: 938940. | General anesthesia with rocuronium and endotracheal intubation. |
| 43 | Rembacken BJ, Axon AT. The role of pethidine in sedation for colonoscopy. *Endoscopy* 1995; 27: 244–7. | Diazepam used for sedation. |
| 44 | Rizzo J, Bernstein D, Gress F. A randomized double-blind placebo-controlled trial evaluating the cost-effectiveness of droperidol as a sedative premedication for EUS. *Gastrointest Endosc* 1999; 50: 178–82. | Droperidol used for sedation. |
| 45 | Samreen A, Waseem A, Azam M, Kazmi IH, Bashir A, Masud S. Comparison of recovery time of propofol and midazolam with propofol alone for sedation in endoscopic retrograde cholangiopancreatography. *Pak J Med Health Sci* 2021; 15: 1024–7. | Inconsistency in reported age data; baseline characteristics not reliable. |
| 46 | Schilling D, Rosenbaum A, Schweizer S, Richter H, Rumstadt B. Sedation with propofol for interventional endoscopy by trained nurses in high-risk octogenarians: a prospective, randomized, controlled study. *Endoscopy* 2009; 41: 295–8. | Possible inclusion of diagnostic EUS. |
| 47 | Seifert H, Schmitt TH, Gültekin T, Caspary WF, Wehrmann T. Sedation with propofol plus midazolam versus propofol alone for interventional endoscopic procedures: a prospective, randomized study. *Aliment Pharmacol Ther* 2000; 14: 1207–14. | Crossover design. |
| 48 | Shin S, Oh TG, Chung MJ, et al. Conventional versus Analgesia-Oriented Combination Sedation on Recovery Profiles and Satisfaction after ERCP: A Randomized Trial. *PLoS One* 2015; 10: e0138422. | Fentanyl vs. Pethidine; both are opioids but not classifiable into separate intervention nodes. |
| 49 | Singh SA, Prakash K, Sharma S, Dhakate G, Bhatia V. Comparison of propofol alone and in combination with ketamine or fentanyl for sedation in endoscopic ultrasonography. *Korean J Anesthesiol* 2018; 71: 43–7. | Possible inclusion of diagnostic EUS. |
| 50 | Song JC, Lu ZJ, Jiao YF, et al. Etomidate Anesthesia during ERCP Caused More Stable Haemodynamic Responses Compared with Propofol: A Randomized Clinical Trial. *Int J Med Sci* 2015; 12: 559–65. | Intramuscular administration used. |
| 51 | Sui Y, Chen X, Ma T, et al. Comparison of three sedation models for same-day painless bidirectional endoscopy: A multicenter randomized controlled trial. *J Gastroenterol Hepatol* 2022; 37: 1603–9. | Intervention focused on sedation strategy during colonoscopy (sedated vs. unsedated). |
| 52 | Sun GQ, Gao BF, Li GJ, Lei YL, Li J. Application of remifentanil for conscious sedation and analgesia in short-term ERCP and EST surgery. *Medicine (Baltimore)* 2017; 96: e6567. | Control group did not receive anesthesia. |
| 53 | Varadarajulu S, Eloubeidi MA, Tamhane A, Wilcox CM. Prospective randomized trial evaluating ketamine for advanced endoscopic procedures in difficult to sedate patients. *Aliment Pharmacol Ther* 2007; 25: 987–97. | Diazepam used for sedation; intervention not consistent with current clinical practice. |
| 54 | Vargo JJ, Zuccaro G Jr, Dumot JA, et al. Gastroenterologist-administered propofol versus meperidine and midazolam for advanced upper endoscopy: a prospective, randomized trial. *Gastroenterology* 2002; 123: 8–16. | Possible inclusion of diagnostic EUS. |
| 55 | Wang P, Chen Y, Guo Y, et al. Comparison of propofol-nalbuphine and propofol-fentanyl sedation for patients undergoing endoscopic retrograde cholangiopancreatography: a double-blind, randomized controlled trial. *BMC Anesthesiol* 2022; 22: 47. | Fentanyl vs. Nalbuphine; both are opioids but not classifiable into separate intervention nodes. |
| 56 | Wille RT, Barnett JL, Chey WD, Scheiman JM, Elta GH. Routine droperidol pre-medication improves sedation for ERCP. *Gastrointest Endosc* 2000; 52: 362–6. | Diazepam used for sedation; intervention not consistent with current clinical practice. |
| 57 | Wu CS, Meng B, Ren HZ. Clinical effects of intravenous anesthesia with etomidate plus propofol for subpyloric endoscopic ultrasonography. *World Chin J Digestol* 2017; 25: 1405–9. | Possible inclusion of diagnostic EUS. |
| 58 | Wu J, Li N, Zhang J, Tang X, Cao X. Safety and efficacy of remifentanil-propofol combination on "muscle relaxant-free" general anesthesia for therapeutic endoscopic retrograde cholangiopancreatography: a randomized controlled trial. *Am J Transl Res* 2023; 15: 5292–303. | Use of cis–atracurium and endotracheal intubation. |
| 59 | Zhang J, Huang Y, Li Z, Li J, Liu K, Li C. Sedation and use of analgesics in endoscopic retrograde cholangiopancreatography: a double-blind comparison study of meperidine/midazolam, remifentanil/ midazolam, and remifentanil alone. *Int J Clin Pharmacol Ther* 2016; 54: 872–9. | Remifentanil vs. Pethidine; both are opioids but not classifiable into separate intervention nodes. |
| 60 | Zhang J, Zhang H, Zheng J, et al. Safety and Effectiveness of Electroacupuncture During Colon Endoscopic Submucosal Dissection: A Randomized Controlled Trial. *J Pain Res* 2025; 18: 1221–9. | Implausible remifentanil dose reported (0.1 mg/kg/min). |
| 61 | Zhao L, Zhang Y, Xu S, Wang X. Comparison Effects of Propofol-Dexmedetomidine versus Propofol-Remifentanil for Endoscopic Ultrasonography: A Prospective Randomized Comparative Trial. *Biomed Res Int* 2022; 2022: 3305696. | Possible inclusion of diagnostic EUS. |
| 62 | Zhong J, Zhang J, Fan Y, et al. Efficacy and safety of Ciprofol for procedural sedation and anesthesia in non-operating room settings. *J Clin Anesth* 2023; 85: 111047. | Study included bronchoscopy. |
| 63 | Zhu H, Qin H, Li DM, Wang B, Zhang ZC, Zhao Q. Analgesic, sedative effects and safety of dezocine in patients undergoing endoscopic retrograde cholangiopancreatography. *World Chin J Digestol* 2015; 23: 1333–7. | Intramuscular administration used. |
| 64 | Zuo XL, Li Z, Liu XP, et al. Propofol vs midazolam plus fentanyl for upper gastrointestinal endomicroscopy: a randomized trial. *World J Gastroenterol* 2012; 18: 1814–27. | Diagnostic upper gastrointestinal endoscopy performed. |

**Table 5 Summary of main characteristics of included studies**

| **Study (Author, Year)** | **Country** | **Intervention groups** | **Sample size**  **(*n*)** | **Age**  **(years)** | **Sex**  **(M/F)** | **Endoscopy type** | **Procedural duration**  **(min)** |
| --- | --- | --- | --- | --- | --- | --- | --- |
| Amir 2024 | India | Propofol + Pentazocine  Dexmedetomidine + Pentazocine | 25  25 | 47.5 ± 13.0  44.4 ± 13.0 | 12/13  14/11 | ERCP | NA |
| Angsuwatcharakon 2012 | Thailand | Midazolam + Pethidine  Midazolam + Propofol + Pethidine | 102  103 | 57.3 ± 14.5  59.6 ± 13.7 | 51/51  52/51 | ERCP | 31.6 ± 17.6  27.9 ±14.4 |
| Aqeel 2024 | Pakistan | Dexmedetomidine + Midazolam  Ketamine + Midazolam + Propofol | 31  31 | 39.2 ± 9.8 | 18/13  15/16 | ERCP | NA |
| Ashikari 2021 | Japan | Propofol  Dexmedetomidine + Propofol | 33  33 | 71.0 [48.0, 88.0]  71.0 [49.0, 88.0] | 28/5  25/8 | ESD | 50.0 [14.0, 153.0]  49.0 [16.0, 108.0] |
| Ates 2021 | Turkey | Midazolam + Propofol  Lidocaine + Midazolam + Propofol | 40  40 | 65.0 [48.0, 72.5]  65.5 [47.0, 73.0] | 18/22  24/16 | ERCP | 19.5 [17.0, 21.5]  20.0 [17.0, 25.0] |
| Babu 2024 | India | Propofol + Fentanyl  Dexmedetomidine + Propofol + Fentanyl | 40  40 | 45.4 ± 13.4  48.1 ± 15.4 | 24/16  27/13 | ERCP | NA |
| Bahrami 2016 | Iran | Ketamine + Midazolam + Propofol  Midazolam + Propofol + Fentanyl | 30  42 | 56.0 ± 19.8  60.5 ± 15.7 | 13/17  20/22 | ERCP | 11.3 ± 7.3  8.9 ± 6.2 |
| Chen 2022 | China | Dexmedetomidine + Propofol + Sufentanil  Propofol + Sufentanil | 24  25 | 72.0 ± 6.1  73.0 ± 5.9 | 10/14  11/14 | ERCP | 49.0 ± 18.2  47.4 ± 18.1 |
| Chun 2012 | Korea | Propofol  Midazolam + Propofol | 67  68 | 64.0 ± 9.3  63.0 ± 11.7 | 45/22  49/19 | ESD | 57.0 ± 34.3  53.0 ± 34.8 |
| Dhingra 2023 | India | Midazolam + Propofol + Fentanyl  Etomidate + Midazolam + Fentanyl | 50  50 | 48.9 ± 13.0  48.8 ± 14.3 | 34/16  34/16 | ERCP | 35.5 ± 11.1  32.2 ± 12.1 |
| Ding 2024 | China | Ciprofol + Sufentanil  Propofol + Sufentanil | 142  142 | 73.6 ± 7.2  73.7 ± 7.2 | 73/69  72/70 | ERCP | 47.4 ± 17.5  48.2 ± 18.3 |
| Dong 2023 | China | Remimazolam + Alfentanil  Propofol + Alfentanil | 250  255 | 67.0 [58.0, 74.0]  68.0 [58.0, 75.0] | 144/106  156/99 | ERCP | 30.0 [24.5, 42.0]  30.0 [21.0, 37.0] |
| Eberl 2020 | Netherlands | Propofol + Alfentanil  Esketamine + Propofol | 79  83 | 58.0 [43.0, 70.0]  63.0 [52.0, 73.0] | 39/40  48/35 | ERCP | 45.0 ± 30.0  47.0 ± 24.0 |
| Fabbri 2012 | Italy | Propofol + Remifentanil  Ketamine + Propofol + Remifentanil | 162  160 | 72.0 ± 8.6  73.0 ± 5.6 | 73/89  75/85 | ERCP | 65.0 ± 25.0  75.0 ± 30.0 |
| Gao 2025 | China | Midazolam + Propofol + Sufentanil  Lidocaine + Midazolam + Propofol + Sufentanil | 95  96 | 73.7 ± 6.8  74.1 ± 7.5 | 55/40  59/37 | ERCP | 45.8 ± 24.0  46.0 ± 19.1 |
| Garg 2019 | India | Propofol  Ketamine + Propofol | 30  30 | 42.7 ± 8.0  39.0 ± 10.7 | 21/9  17/13 | ERCP | 48.3 ± 8.2  46.5 ± 7.5 |
| Goyal 2016 | India | Propofol + Fentanyl  Ketamine + Dexmedetomidine | 42  41 | 59.2 ± 14.4  60.4 ± 13.8 | 29/13  30/11 | ERCP | 42.6 ± 13.7  41.8 ± 15.6 |
| Han 2017 | Korea | Midazolam + Fentanyl  Propofol + Fentanyl | 50  50 | 84.2 ± 4.3  84.0 ± 3.6 | 30/20  22/28 | ERCP | 24.9 ± 10.3  28.4 ± 14.1 |
| Han 2019 | Korea | Etomidate + Midazolam + Fentanyl  Midazolam + Propofol + Fentanyl | 94  92 | 63.9 ± 13.1  60.6 ± 12.8 | 62/32  61/31 | ESD or EMR or ERCP | 28.5 ± 15.4  24.2 ± 15.8 |
| Hasanein 2013 | Egypt | Ketamine + Propofol  Propofol + Fentanyl | 100  100 | 57.7 ± 13.3  56.9 ± 11.9 | 49/51  50/50 | ERCP | 31.6 ± 17.7  27.9 ± 14.4 |
| Haytural 2015 | Turkey | Propofol  Propofol + Remifentanil  Propofol + Fentanyl | 30  30  30 | 51.6 ± 12.9  52.1 ± 16.4  50.3 ± 13.3 | 18/12  18/12  11/19 | ERCP | 25.0 (15.0 to 50.0)  25.0 (15.0 to 50.0)  25.0 (15.0 to 50.0) |
| Heidari 2014 | Iran | Ketamine+ Fentanyl  Propofol + Remifentanil | 34  37 | 55.3 ± 15.1  54.5 ± 11.1 | 18/16  18/19 | ERCP | NA |
| Jung 2000 | Germany | Midazolam  Propofol | 40  40 | 63.0 (37.0 to 88.0)  62.0 (33.0 to 86.0) | 13/27  21/19 | ERCP | 23.0 (9.0 to 50.0)  27.0 (8.0 to 60.0) |
| Kilic 2011 | Turkey | Midazolam + Fentanyl  Dexmedetomidine + Fentanyl | 25  25 | 53.7±18.3  57.0±14.6 | 16/9  15/10 | ERCP | 25.1 ± 8.0  25.8 ± 8.9 |
| Kim 2015 | Korea | Dexmedetomidine + Remifentanil  Propofol + Remifentanil | 29  30 | 62.1 ± 10.3  62.9 ± 12.3 | 19/10  22/8 | ESD | 42.8 ± 26.7  37.6 ± 18.5 |
| Kim 2016 | Korea | Propofol + Fentanyl  Lidocaine + Propofol + Fentanyl | 31  30 | 65.0 ± 9.0  65.2 ± 8.5 | 21/10  24/6 | ESD | 40.0 ± 15.0  41.0 ± 14.0 |
| Koruk 2020 | Turkey | Midazolam + Propofol  Dexmedetomidine + Propofol | 20  20 | 50.8 ±16.1  53.3 ±11.0 | 9/11  9/11 | ERCP | 16.7 ± 4.2  18.8 ± 5.5 |
| Lee 2011 | Korea | Midazolam + Pethidine  Midazolam + Propofol + Pethidine | 104  102 | 62.5 ± 13.0  62.7 ± 13.4 | 56/48  60/42 | Therapeutic EGD or ERCP | 26.6 ± 15.6  30.6 ± 20.7 |
| Lee 2012 | Korea | Propofol  Midazolam + Propofol + Fentanyl | 104  102 | 67.5 ± 13.6  65.1 ± 15.4 | 61/43  59/43 | Therapeutic PEP | 27.4 ± 14.4  28.7 ± 15.6 |
| Lee 2014 | Korea | Midazolam + Pethidine  Dexmedetomidine + Midazolam + Pethidine | 57  53 | 60.1 ± 12.0  61.3 ± 12.3 | 33/24  31/22 | ERCP | 19.0 ± 10.0  16.0 ± 9.0 |
| Lee 2023 | Korea | Remimazolam  Propofol | 55  55 | 69.0 (38.0 to 88.0)  70.0 (27.0 to 92.0) | 32/23  32/23 | ERCP | 16.2 ± 7.2  16.5 ± 7.7 |
| Lee SJ 2015 | Korea | Propofol + Fentanyl  Midazolam + Propofol + Fentanyl | 80  80 | 66.5 ± 9.2  65.4 ± 8.4 | 42/38  54/26 | ERCP | 24.0 ± 11.2  26.4 ± 12.3 |
| Lee SP 2015 | Korea | Midazolam + Pethidine  Dexmedetomidine + Midazolam + Pethidine | 40  40 | 64.4 ± 9.9  64.0 ± 9.4 | 27/13  31/9 | ESD | 100.5 ± 68.5  114.4 ± 74.8 |
| Liu 2020 | China | Midazolam + Propofol + Sufentanil  Lidocaine + Midazolam + Propofol + Sufentanil | 24  24 | 62.2 ± 15.4  60.6 ± 13.7 | 17/7  18/6 | ERCP | 34.8 ± 17.5  37.3 ± 18.1 |
| Lu 2018 | China | Midazolam + Remifentanil  Dexmedetomidine + Midazolam + Remifentanil | 86  108 | 60.6 ± 13.1  60.5 ± 14.5 | 47/39  55/53 | ERCP | 41.5 ± 19.6  35.5 ± 16.2 |
| Mukhopadhyay 2015 | India | Midazolam + Propofol  Ketamine + Midazolam + Propofol + Pentazocine  Ketamine + Dexmedetomidine + Midazolam + Propofol + Pentazocine | 15  15  15 | 61.9 ± 3.8  64.0 ± 8.4  62.1 ± 10.0 | 7/8  6/9  7/8 | ERCP | 86.3 ± 14.4  80.0 ± 17.2  84.0 ± 16.0 |
| Muller 2008 | Brazil | Propofol + Fentanyl  Dexmedetomidine + Fentanyl | 14  12 | 56.6 ± 15.6  57.3 ± 15.0 | 2/12  5/7 | ERCP | 19.9 ± 5.9  24.3 ± 7.0 |
| Narayanan 2015 | UK | Midazolam + Pethidine  Ketamine + Midazolam | 18  19 | 69.0 [64.0, 75.0]  64.0 [59.0, 79.0] | 11/7  6/13 | ERCP | 25.5 [17.0, 30.0]  21.0 [15.0, 34.0] |
| Nonaka 2018 | Japan | Propofol  Dexmedetomidine + Propofol | 29  29 | 74.0 (60.0 to 86.0)  71.0 (52.0 to 86.0) | 24/5  23/6 | ESD | 41.0 (11.0 to 405.0)  39 (5.0 to 304.0) |
| Pushkarna 2019 | India | Midazolam + Propofol  Dexmedetomidine + Propofol | 30  30 | 47.2 ± 14.3  45.3 ± 16.4 | 15/15  17/13 | ERCP | 32.4 ± 9.5  30.6 ± 10.2 |
| Ramkiran 2015 | India | Midazolam + Propofol  Dexmedetomidine + Midazolam + Propofol  Ketamine + Midazolam + Propofol | 24  24  24 | 52.7 ± 15.2  55.0 ± 13.5  44.8 ± 14.5 | 7/17  13/11  9/15 | ERCP | 29.2 ± 11.6  35.2 ± 13.2  28.3 ± 11.9 |
| Riphaus 2005 | Germany | Propofol  Midazolam + Pethidine | 77  78 | 83.7 ± 7.8  85.6 ± 8.1 | 35/42  32/46 | ERCP | 29.0 ± 19.0  30.0 ± 19.0 |
| Sasaki 2012 | Japan | Midazolam + Pethidine  Propofol + Pethidine | 90  88 | 71.0 (45.0 to 84.0)  70.0 (46.0 to 83.0) | 60/30  64/24 | ESD | 77.3 ± 37.0  82.1 ± 36.1 |
| Sethi 2014 | India | Dexmedetomidine + Fentanyl  Midazolam + Fentanyl | 30  30 | 44.0 ± 12.0  42.0 ± 13.0 | 14/16  13/17 | ERCP | NA |
| Shin 2017 | Korea | Propofol + Fentanyl  Midazolam + Propofol + Fentanyl | 36  36 | 64.0 (49.0 to 77.0)  60.0 (45.0 to 78.0) | 24/12  26/10 | ESD | 29.7 ± 16.0  35.3 ± 25.2 |
| Singh 2022 | India | Ketamine + Dexmedetomidine + Midazolam  Ketamine + Midazolam + Propofol | 42  42 | 44.0 [34.0, 54.0]  51 [38.0, 60.0] | 22/20  22/20 | ERCP | 45.0 [35.0, 60.0]  45.0 [35.0, 60.0] |
| Singh 2023 | India | Ketamine + Etomidate + Midazolam  Ketamine + Dexmedetomidine + Midazolam | 30  30 | 50.1 ± 13.0  50.6 ± 12.6 | 10/20  6/24 | ERCP | 24.5 ± NCR  21.8 ± NCR |
| Soliman 2024 | Egypt | Propofol  Dexmedetomidine | 101  101 | 46.0 ± 12.0  45.2 ± 12.8 | 58/43  53/48 | ERCP | 44.0 ± 2.0  45.0 ± 1.0 |
| Srivastava 2018- | India | Propofol + Fentanyl  Midazolam + Propofol  Dexmedetomidine + Propofol | 33  32  32 | 49.1 ± 10.8  44.7 ± 13.0  46.5 ± 11.4 | 19/16  17/18  15/20 | ERCP | 30.5 ± 6.0  30.8 ± 6.9  29.8 ± 6.5 |
| Srivastava 2021- | India | Propofol + Fentanyl  Dexmedetomidine + Fentanyl | 30  30 | 46.1 ± 11.4  48.6 ± 10.5 | 16/14  20/10 | ERCP | NA |
| Sun 2024 | China | Propofol + Nalbuphine  Dexmedetomidine + Propofol + Nalbuphine | 40  40 | 68.0 ± 1.9  68.2 ± 2.2 | 20/20  19/21 | ERCP | 89.6 ± 12.0  92.0 ± 11.9 |
| Takimoto 2011 | Japan | Dexmedetomidine  Propofol  Midazolam | 30  30  30 | 70.0 (52.0 to 80.0)  69.0 (47.0 to 79.0)  72.0 (48.0 to 80.0) | 13/17  14/16  15/15 | ESD | NCR |
| Tian 2024 | China | Propofol + Sufentanil + Remifentanil  Remimazolam + Sufentanil + Remifentanil | 70  70 | 78.0 ± 3.6  78.1 ± 4.0 | 39/31  40/30 | ERCP | 36.8 ± 5.6  36.5 ± 6.2 |
| Wang 2024 | China | Dexmedetomidine + Propofol + Sufentanil  Dexmedetomidine + Ciprofol + Sufentanil | 153  153 | 54.8 ± 13.6  55.0 ± 13.0 | 86/67 77/76 | ERCP | NA |
| Wehrmann 1999 | Germany | Midazolam + Pentazocine  Propofol | 98  99 | 61.2 ± 20.5  63.6 ± 23.3 | 40/60  42/58 | ERCP | 32.0 ± 14.0  27.0 ± 16.0 |
| Xiao 2024 | China | Remimazolam + Remifentanil  Propofol + Remifentanil | 40  40 | 54.0 [45.0, 63.0]  59.0 [51.0, 67.0] | 22/18  24/16 | ERCP | 40.0 [32.0, 64.0]  35.0 [30.0, 35.0] |
| Xin 2024 | China | Remimazolam + Alfentanil  Propofol + Alfentanil | 53  55 | 81.5 ±4.9  82.3 ± 6.0 | 33/20  26/29 | ERCP | 38.8 ± 8.9  38.1 ± 9.8 |
| Yüksel 2007 | Turkey | Midazolam  Midazolam + Pethidine | 48  48 | 55.5 ± 14.7  54.6 ± 18.4 | 27/21  28/20 | ERCP | 29.5 ± 15.7  30.5 ± 15.3 |
| Zhang 2024 | China | Dexmedetomidine + Midazolam + Sufentanil  Midazolam + Propofol + Sufentanil | 20  21 | 57.1 ± 16.1  61.0 ± 12.8 | 9/11  9/12 | ERCP | 23.3 ± 11.5  25.5 ± 14.7 |
| Zhou 2024 | China | Remimazolam + Remifentanil + Butorphanol  Propofol + Remifentanil + Butorphanol | 51  51 | 61.2 ±12.2  60.0 ±12.4 | 23/28  26/25 | ERCP | 75.5 ±34.1  77.0 ±33.3 |

Abbreviations: EGD - esophagogastroduodenoscopy; EMR - endoscopic mucosal resection; ERCP - endoscopic retrograde cholangiopancreatography; ESD - endoscopic submucosal dissection; PEP - pancreaticobiliary endoscopic procedures; Note: Values are presented as median [interquartile range] or median (range), as reported in the original studies.

**Table 6 Pharmacological Interventions and Abbreviations**

| **No.** | **Pharmacological Intervention** | **Abbreviation** |
| --- | --- | --- |
| 1 | Ciprofol-opioid | CFL-OP |
| 2 | Dexmedetomidine | DEX |
| 3 | Dexmedetomidine-ciprofol-opioid | DEX-CFL-OP |
| 4 | Dexmedetomidine-midazolam | DEX-MDZ |
| 5 | Dexmedetomidine-midazolam-opioid | DEX-MDZ-OP |
| 6 | Dexmedetomidine-midazolam-propofol | DEX-MDZ-PF |
| 7 | Dexmedetomidine-opioid | DEX-OP |
| 8 | Dexmedetomidine-propofol | DEX-PF |
| 9 | Dexmedetomidine-propofol-opioid | DEX-PF-OP |
| 10 | Esktamine-propofol | ESK-PF |
| 11 | Etomidate-midazolam-opioid | ETM-MDZ-OP |
| 12 | Ketamine-dexmedetomidine | KET-DEX |
| 13 | Ketamine-dexmedetomidine-midazolam | KET-DEX-MDZ |
| 14 | Ketamine-dexmedetomidine-midazolam-propofol-opioid | KET-DEX-MDZ-PF-OP |
| 15 | Ketamine-etomidate-midazolam | KET-ETM-MDZ |
| 16 | Ketamine-midazolam | KET-MDZ |
| 17 | Ketamine-midazolam-propofol | KET-MDZ-PF |
| 18 | Ketamine-midazolam-propofol-opioid | KET-MDZ-PF-OP |
| 19 | Ketamine-opioid | KET-OP |
| 20 | Ketamine-propofol | KET-PF |
| 21 | Ketamine-propofol-opioid | KET-PF-OP |
| 22 | Lidocaine-midazolam-propofol | LIDO-MDZ-PF |
| 23 | Lidocaine-midazolam-propofol-opioid | LIDO-MDZ-PF-OP |
| 24 | Lidocaine-propofol-opioid | LIDO-PF-OP |
| 25 | Midazolam | MDZ |
| 26 | Midazolam-opioid | MDZ-OP |
| 27 | Midazolam-propofol | MDZ-PF |
| 28 | Midazolam-propofol-opioid | MDZ-PF-OP |
| 29 | Propofol | PF |
| 30 | Propofol-opioid | PF-OP |
| 31 | Remimazolam | RMZ |
| 32 | Remimazolam-opioid | RMZ-OP |

**Table 7 Pharmacological regimens and dosage details**

| **Study (Author, Year)** | **Induction Agents** | **Induction Doses** | **Maintenance Agents** | **Maintenance Doses** | **Rescue Agents** | **Rescue Doses** |
| --- | --- | --- | --- | --- | --- | --- |
| Amir 2024 | Propofol  Pentazocine | 1.0 mg kg^-1^  0.3–0.5 mg kg^-1^ | Propofol | 1.0 mg kg^-1^ h^-1^ | – | – |
|  | Dexmedetomidine  Pentazocine | 1.0 μg kg^-1^  0.3–0.5 mg kg^-1^ | Dexmedetomidine | 0.5 μg kg^-1^ h^-1^ | – | – |
| Angsuwatcharakon 2012 | Midazolam  Pethidine | 2.5 mg  25.0 mg | Midazolam  Pethidine | 2.5 mg  25.0 mg | Midazolam  Pethidine | 2.5 mg  25.0 mg |
|  | Midazolam  Propofol  Pethidine | 2.5 mg  1.0 mg kg^-1^  25.0 mg | Propofol | 1.0 mg kg^-1^ h^-1^ | Propofol | 0.5 mg kg^-1^ |
| Aqeel 2024 | Dexmedetomidine  Midazolam | 1.0 μg kg^-1^  0.05 mg kg^-1^ | Dexmedetomidine | 0.5 μg kg^-1^ h^-1^ | – | – |
|  | Ketamine  Midazolam  Propofol | 1.0 mg kg^-1^  0.05 mg kg^-1^  1.0 mg kg^-1^ | Ketamine  Propofol | 1.0 mg kg^-1^  1.0 mg kg^-1^ | Ketamine  Propofol | 1.0 mg kg^-1^  1.0 mg kg^-1^ |
| Ashikari 2021 | Propofol | 20.0 mg bolus^-1^ | Propofol | 2.0 mg kg^-1^ h^-1^ | Propofol | 1.0 mg/kg/h |
|  | Dexmedetomidine  Propofol | 6 μg kg^-1^ h^-1^  20.0 mg bolus^-1^ | Dexmedetomidine  Propofol | 0.7 μg kg^-1^ h^-1^  2.0 mg kg^-1^ h^-1^ | Propofol | 1.0 mg kg^-1^ h^-1^ |
| Ates 2021 | Midazolam  Propofol | 1.0 mg  1.0 mg kg^-1^ | Propofol | 10.0–20.0 mg | Ketamine | 0.25 mg kg^-1^ |
|  | Lidocaine  Midazolam  Propofol | 1.5 mg kg^-1^  1.0 mg  1.0 mg kg^-1^ | Propofol | 10.0–20.0 mg | Ketamine | 0.25 mg kg^-1^ |
| Babu 2024 | Propofol  Fentanyl | 1.0 mg kg^-1^  1.0 μg kg^-1^ | Propofol | 50.0 μg kg^-1^ min^-1^ | Propofol | 20.0 mg |
|  | Dexmedetomidine  Propofol  Fentanyl | 1.0 μg kg^-1^  1.0 mg kg^-1^  1.0 μg kg^-1^ | Propofol | 50.0 μg kg^-1^ min^-1^ | Propofol | 20.0 mg |
| Bahrami 2016 | Ketamine  Midazolam  Propofol | 0.5 mg kg^-1^  0.5–1.0 mg  0.5 mg kg^-1^ | Propofol | 75.0 μg kg^-1^ min^-1^ | Propofol | 20.0 mg |
|  | Midazolam  Propofol  Fentanyl | 0.5–1.0 mg  0.5 mg kg^-1^  50.0–100.0 μg | Propofol | 75.0 μg kg^-1^ min^-1^ | Propofol | 20.0 mg |
| Chen 2022 | Propofol  Sufentanil | 1.0–2.0 mg kg^-1^  0.1 μg/kg | Propofol | 1.0–2.0μg ml^-1^ | – | – |
|  | Dexmedetomidine  Propofol  Sufentanil | 0.5 μg kg^-1^  1.0–2.0 mg kg^-1^  0.1 μg kg^-1^ | Propofol | 1.0–2.0μg ml^-1^ | – | – |
| Chun 2012 | Propofol | 0.3 mg kg^-1^ min^-1^ | Propofol | 0.3 mg kg^-1^ min^1^ | – | – |
|  | Midazolam  Propofol | 2 mg  0.3 mg kg^-1^ min^-1^ | Propofol | 0.3 mg kg^-1^ min^-1^ | – | – |
| Demir 2024 | Midazolam  Propofol | 0.02 mg kg^-1^  1.0 mg kg^-1^ | Propofol | 0.5 mg kg^-1^ h^-1^ | Propofol | 0.25 mg kg^-1^ |
|  | Ketamine  Midazolam  Propofol | 0.34 mg kg^-1^ h^-1^  0.02 mg kg^-1^  1.0 mg kg^-1^ | Ketamine  Propofol | 0.17 mg kg^-1^ h^-1^  0.5 mg kg^-1^ h^-1^ | Propofol | 0.25 mg kg^-1^ |
| Dhingra 2023 | Midazolam  Propofol  Fentanyl | 0.02 mg kg^-1^  1.5–2.0 mg kg^-1^  1.0 μg kg^-1^ | Propofol | 50.0–75.0 μg kg^-1^ min^-1^ | – | – |
|  | Etomidate  Midazolam  Fentanyl | 0.15–0.2 mg kg^-1^  0.02 mg kg^-1^  1.0 μg kg^-1^ | Etomidate | 5.0–10.0 μg kg^-1^ min^-1^ | – | – |
| Ding 2024 | Ciprofol  Sufentanil | 0.3–0.4 mg kg^-1^  0.1 μg kg^-1^ | Ciprofol | 0.8–1.2 mg kg^-1^ h^-1^ | Ciprofol | 0.15–0.2 mg kg^-1^ |
|  | Propofol  Sufentanil | 1.5–2.0 mg kg^-1^  0.1 μg kg^-1^ | Propofol | 4.0–12.0 mg kg^-1^ h^-1^ | Propofol | 0.75–1.0 mg kg^-1^ |
| Dong 2023 | Remimazolam  Alfentanil | 0.3 mg kg^-1^  10.0 μg kg^-1^ | Remimazolam  Alfentanil | 0.2–1.0 mg kg^-1^ h^-1^  0–1.0 μg kg^-1^ min^-1^ | Remimazolam  Alfentanil | 0.1 mg kg^-1^  5.0 μg kg^-1^ |
|  | Propofol  Alfentanil | 1.5–2.0 mg kg^-1^  10.0 μg kg^-1^ | Propofol  Alfentanil | 2.0–6.0 mg kg^-1^ h^-1^  0–1.0 μg kg^-1^ min^-1^ | Propofol  Alfentanil | 0.5 mg kg^-1^  5.0 μg kg^-1^ |
| Eberl 2020 | Propofol  Alfentanil | 1.5 μg ml^-1^  2.0 μg kg^-1^ | Propofol | 1.5–2.5 μg ml^-1^ | Propofol  Alfentanil | 0.5 μg/ml  1.0 μg kg^-1^ |
|  | Esketamine  Propofol | 0.15 mg kg^-1^  1.5 μg ml^-1^ | Propofol | 1.5–2.5 μg ml^-1^ | Esketamine  Propofol | 0.05 mg kg^-1^  0.5 μg/ml |
| Fabbri 2012 | Propofol  Remifentanil | 1.0 mg kg^-1^  0.25 μg kg^-1^ min^-1^ | Propofol  Remifentanil | 1.0 mg kg^-1^ h^-1^  0.25 μg kg^-1^ min^-1^ | Propofol | 0.25 mg kg^-1^ |
|  | Ketamine  Propofol  Remifentanil | 5.0 μg kg^-1^ min^-1^  1.0 mg kg^-1^  0.1 μg kg^-1^ min^-1^ | Ketamine  Propofol  Remifentanil | 5.0 μg kg^-1^ min^-1^  0.1 μg kg^-1^ min^-1^ | Propofol | 0.25 mg kg^-1^ |
| Gao 2025 | Midazolam  Propofol  Sufentanil | 0.01 mg kg^-1^  2.0 μg ml^-1^  0.1 μg kg^-1^ | Propofol | 2.0μg ml^-1^ | – | – |
|  | Lidocaine  Midazolam  Propofol  Sufentanil | 1.0 mg kg^-1^  0.01 mg kg^-1^  2.0 μg ml^-1^  0.1 μg kg^-1^ | Lidocaine  Propofol | 2.0 mg kg^-1^ h^-1^  2.0 μg ml^-1^ | – | – |
| Garg 2019 | Propofol | 20 mg | Propofol | 50–150 μg kg^-1^ h^-1^ | – | – |
|  | Ketamine  Propofol | 25 mg  20 mg | Propofol | 50–150 μg kg^-1^ h^-1^ | – | – |
| Goyal 2016 | Propofol  Fentanyl | 1.0 mg kg^-1^  1.0 μg kg^-1^ | Propofol | 2.0–4.0 mg kg^-1^ h^-1^ | Propofol | 0.25 mg kg^-1^ |
|  | Ketamine  Dexmedetomidine | 1.0 mg kg^-1^  0.5 μg kg^-1^ | Ketamine  Dexmedetomidine | 1.0–2.0 mg kg^-1^ h^-1^  0.5 μg kg^-1^ h^-1^ | Ketamine | 0.25 mg kg^-1^ |
| Han 2017 | Midazolam  Fentanyl | 0.05 mg kg^-1^  12.5–25.0 μg | Midazolam | 0.5–1.0 mg | Midazolam | 0.5–1.0 mg |
|  | Propofol  Fentanyl | 0.5 mg kg^-1^  12.5–25.0 μg | Propofol | 5.0–10.0 mg | Propofol | 5.0–10.0 mg |
| Han 2019 | Midazolam  Propofol  Fentanyl | 0.05 mg kg^-1^  10.0–20.0 mg  0.5–1.0 μg kg^-1^ | Propofol | 10.0–20.0 mg | Propofol | 10.0–20.0 mg |
|  | Etomidate  Midazolam  Fentanyl | 1.0–4.0 mg  0.05 mg kg^-1^  0.5–1.0 μg kg^-1^ | Etomidate | 1.0–4.0 mg | Etomidate | 1.0–4.0 mg |
| Hasanein 2013 | Ketamine  Propofol | 0.125 mg kg^-1^  0.5 mg kg^-1^ | Ketamine  Propofol | 12.5 μg kg^-1^ min^-1^  50.0 μg kg^-1^ min^-1^ | – | – |
|  | Propofol  Fentanyl | 0.5 mg kg^-1^  1.5 μg kg^-1^ | Propofol | 50.0 μg kg^-1^ min^-1^ | – | – |
| Haytural 2015 | Propofol | 1.5 mg kg^-1^ | Propofol | 1.0 mg kg^-1^ h^-1^ | Propofol | 0.5 mg kg^-1^ |
|  | Propofol  Remifentanil | 1.5 mg kg^-1^  0.05 μg kg^-1^ min^-1^ | Propofol | 1.0 mg kg^-1^ h^-1^ | Propofol | 0.5 mg kg^-1^ |
|  | Propofol  Fentanyl | 1.5 mg kg^-1^  1.0 μg kg^-1^ | Propofol | 1.0 mg kg^-1^ h^-1^ | Propofol | 0.5 mg kg^-1^ |
| Heidari 2014 | Ketamine  Fentanyl | 0.5 mg kg^-1^  1.0 μg kg^-1^ | Propofol | 50.0 μg kg^-1^ min^-1^ | – | – |
|  | Propofol  Remifentanil | 1.0 mg kg^-1^  0.05 μg kg^-1^ min^-1^ | Propofol | 50.0 μg kg^-1^ min^-1^ | – | – |
| Jung 2000 | Midazolam | 3.0–5.0 mg | Midazolam | 3.0–5.0 mg  (max 15 mg) | – | – |
|  | Propofol | 0.5–3.0 mg kg^-1^ | Propofol | 4.0–8.0 mg kg^-1^ h^-1^ | – | – |
| Kilic 2011 | Midazolam  Fentanyl | 0.04 mg kg^-1^  1.0 μg kg^-1^ | Midazolam | 0.5 mg | Midazolam | 0.5 mg |
|  | Dexmedetomidine  Fentanyl | 1.0 μg kg^-1^ h^-1^  1.0 μg kg^-1^ | Dexmedetomidine | 0.2–0.7 μg kg^-1^ h^-1^ | – | – |
| Kim 2015 | Dexmedetomidine  Remifentanil | 0.5 μg kg^-1^  6.0 μg kg^-1^ h^-1^ | Dexmedetomidine | 0.3–0.7 μg kg^-1^ h^-1^ | Propofol  Remifentanil | 10.0 mg  0.1 μg kg^-1^ |
|  | Propofol  Remifentanil | 0.5 mg kg^-1^  6.0 μg kg^-1^ h^-1^ | Propofol | 30.0 μg kg^-1^ min^-1^ | Propofol  Remifentanil | 10.0 mg  0.1 μg kg^-1^ |
| Kim 2016 | Propofol  Fentanyl | 0.5 mg kg^-1^  1.0 μg kg^-1^ | Propofol  Fentanyl | 60.0 μg kg^-1^ min^-1^  0.5μg kg^-1^ | – | – |
|  | Lidocaine  Propofol  Fentanyl | 1.5 mg kg^-1^  0.5 mg kg^-1^  1.0 μg kg^-1^ | Lidocaine  Propofol  Fentanyl | 2.0 mg kg^-1^ h^-1^  60.0 μg kg^-1^ min^-1^  0.5 μg kg^-1^ | – | – |
| Koruk 2020 | Midazolam  Propofol | 0.05 mg kg^-1^  1.0–1.5 mg kg^-1^ | Propofol | 20.0 mg | Propofol | 20.0 mg |
|  | Dexmedetomidine  Propofol | 1.0 μg kg^-1^  1.0–1.5 mg kg^-1^ | Propofol | 20.0 mg | Propofol | 20.0 mg |
| Lee 2011 | Midazolam  Pethidine | 0.05 mg kg^-1^  12.5–25.0 mg | Midazolam | 1.0–2.0 mg | Midazolam | 1.0–2.0 mg |
|  | Midazolam  Propofol  Pethidine | 0.05 mg kg^-1^  0.5 mg kg^-1^  12.5–25.0 mg | Propofol | 10.0–20.0 mg | Propofol | 10.0–20.0 mg |
| Lee 2012 | Propofol | 0.5 mg kg^-1^ | Propofol | 10.0–20.0 mg | Propofol | 10.0–20.0 mg |
|  | Midazolam  Propofol  Fentanyl | 1.0 mg  10.0–20.0 mg  25.0–50.0 μg | Propofol | 10.0–20.0 mg | Propofol | 10.0–20.0 mg |
| Lee 2014 | Midazolam  Pethidine | 0.06 mg kg^-1^  25.0–50.0 mg | Midazolam | 1.0 mg | Midazolam | 1.0 mg |
|  | Dexmedetomidine  Midazolam  Pethidine | 1.0 μg kg^-1^ h^-1^  0.06 mg kg^-1^  25.0–50.0 mg | Midazolam | 1.0 mg | Midazolam | 1.0 mg |
| Lee 2023 | Remimazolam | 5.0 mg | Remimazolam | 2.5 mg | Remimazolam | 2.5 mg |
|  | Propofol | 0.5 mg kg^-1^ | Propofol | 0.5 mg kg^-1^ | Propofol | 0.5 mg kg^-1^ |
| Lee SJ 2015 | Propofol  Fentanyl | 0.5 mg kg^-1^  25.0–50.0 μg | Propofol | 10.0–20.0 mg | Propofol | 10.0–20.0 mg |
|  | Midazolam  Propofol  Fentanyl | 0.05 mg kg^-1^  10.0–20.0 mg  25.0–50.0 μg | Propofol | 10.0–20.0 mg | Propofol | 10.0–20.0 mg |
| Lee SP 2015 | Midazolam  Pethidine | 0.05 mg kg^-1^  50.0 mg | Midazolam  Pethidine | NA  25.0–50.0 mg | – | – |
|  | Dexmedetomidine  Pethidine | 1.0 μg kg^-1^  50.0 mg | Dexmedetomidine  or  Midazolam  Pethidine | 0.2–0.7 μg kg^-1^ h^-1^  or  0.5–1.0 mg  25.0–50.0 mg | – | – |
| Liu 2020 | Midazolam  Propofol  Sufentanil | 0.02 mg kg^-1^  0.25–0.75 mg kg^-1^  0.1 μg kg^-1^ | Propofol | 2.0–4.0 mg kg^-1^ h^-1^ | Propofol | 20.0–30.0 mg |
|  | Lidocaine  Midazolam  Propofol  Sufentanil | 1.5 mg kg^-1^  0.02 mg kg^-1^  0.25–0.75 mg kg^-1^  0.1 μg kg^-1^ | Lidocaine  Propofol | 2.0 mg kg^-1^ h^-1^  2.0–4.0 mg kg^-1^ h^-1^ | Propofol | 20.0–30.0 mg |
| Lu 2018 | Midazolam Remifentanil | 0.05 mg kg^-1^  0.05–0.2 μg kg^-1^ min^-1^ | Midazolam  Remifentanil | 0.02 mg kg^-1^  0.05–0.2 μg kg^-1^ min^-1^ | – | – |
|  | Dexmedetomidine  Remifentanil | 0.5–1.0 μg kg^-1^  0.05–0.2 μg kg^-1^ min^-1^ | Midazolam  Remifentanil | 0.02 mg kg^-1^  0.05–0.2 μg kg^-1^ min^-1^ | – | – |
| Mukhopadhyay 2015 | Midazolam  Propofol | 1.0 mg  0.75–1.0 mg kg^-1^ | Propofol | 10.0–20.0 mg | Propofol | 10.0–20.0 mg |
|  | Ketamine  Midazolam  Propofol  Pentazocine | 25.0 mg  0.5 mg  0.75–1.0 mg kg^-1^  6.0 mg | Propofol | 10.0–20.0 mg | Propofol | 10.0–20.0 mg |
|  | Ketamine  Dexmedetomidine  Midazolam  Propofol  Pentazocine | 25.0 mg  1.0 μg kg^-1^  0.5 mg  0.75–1.0 mg kg^-1^  6.0 mg | Dexmedetomidine  Propofol | 0.2–0.5 μg kg^-1^ h^-1^  10.0–20.0 mg | Propofol | 10.0–20.0 mg |
| Muller 2008 | Propofol  Fentanyl | 4.0 μg ml^-1^  1.0 μg kg^-1^ | Propofol  Fentanyl | 1.0–4.0 μg ml^-1^  50.0–100.0 μg | Propofol  Fentanyl | 1.0–2.0 mg kg^-1^  50.0–100.0 μg |
|  | Dexmedetomidine | 1.0 μg kg^-1^ | Dexmedetomidine  Fentanyl | 0.2–0.5 μg kg^-1^ min^-1^  50.0–100.0 μg | Propofol  Fentanyl | 1.0–2.0 mg kg^-1^  50.0–100.0 μg |
| Narayanan 2015 | Midazolam  Pethidine | 1.0–2.0 mg  25.0 mg | Midazolam  Pethidine | 1.0–2.0 mg  25.0 mg | Midazolam  Pethidine | 1.0–2.0 mg  25.0 mg |
|  | Ketamine  Midazolam | 12.5–25.0 mg  1.0–2.0 mg | Ketamine  Midazolam | 12.5–25.0 mg  1.0–2.0 mg | Ketamine  Midazolam | 12.5–25.0 mg  1.0–2.0 mg |
| Nonaka 2018 | Propofol | 20.0 mg | Propofol | 2.0 mg kg^-1^ h^-1^ | Propofol | 20.0 mg |
|  | Dexmedetomidine  Propofol | 6.0 μg kg^-1^ h^-1^  20.0 mg | Dexmedetomidine  Propofol | 0.5 μg kg^-1^ h^-1^  2.0 mg kg^-1^ h^-1^ | Propofol | 20.0 mg |
| Pushkarna 2019 | Midazolam | 0.05 mg kg^-1^ | Propofol | 10.0 mg | Propofol | 10.0 mg |
|  | Dexmedetomidine | 1.0 μg kg^-1^ | Dexmedetomidine  Propofol | 0.5 μg kg^-1^ h^-1^  10.0 mg | Propofol | 10.0 mg |
| Ramkiran 2015 | Midazolam  Propofol | 0.05 mg kg^-1^  0.5–1.5 mg kg^-1^ | Propofol | 20.0 mg | Propofol | 20.0 mg |
|  | Dexmedetomidine  Midazolam  Propofol | 1.0 μg kg^-1^  0.05 mg kg^-1^  0.5–1.5 mg kg^-1^ | Dexmedetomidine  Propofol | 0.5 μg kg^-1^ h^-1^  20.0 mg | Propofol | 20.0 mg |
|  | Ketamine  Midazolam  Propofol | 0.25 mg kg^-1^  0.05 mg kg^-1^  0.5–1.5 mg kg^-1^ | Ketamine  Propofol | 5.0 μg kg^-1^ min^-1^  20.0 mg | Propofol | 20.0 mg |
| Riphaus 2005 | Propofol | 40.0–60.0 mg | Propofol | 20.0 mg | Propofol | 20.0 mg |
|  | Midazolam  Pethidine | 0.05 mg kg^-1^  25.0 mg | Midazolam  Pethidine | 2.5–3.5 mg  25.0 mg | Midazolam  Pethidine | 2.5–3.5 mg  25.0 mg |
| Sasaki 2012 | Midazolam  Pethidine | 1.0–2.0 mg  35.0 mg | Midazolam | 1.0–2.0 mg | Midazolam | 1.0–2.0 mg |
|  | Propofol  Pethidine | 20.0 mg  35.0 mg | Propofol | 10.0–20.0 mg | Propofol | 10.0–20.0 mg |
| Sethi 2014 | Dexmedetomidine  Fentanyl | 1.0 μg kg^-1^  1.0 μg kg^-1^ | Dexmedetomidine | 0.5 μg kg^-1^ h^-1^ | Propofol | 10.0 mg |
|  | Midazolam  Fentanyl | 0.04 mg kg^-1^  1.0 μg kg^-1^ | Midazolam | 0.5 mg | Propofol | 10.0 mg |
| Shin 2017 | Propofol  Fentanyl | 0.5 mg kg^-1^  1.0 μg kg^-1^ | Propofol | 2.0 mg kg^-1^ h^-1^ | Propofol  Fentanyl | 0.25 mg kg^-1^  0.5 μg kg^-1^ |
|  | Midazolam  Propofol  Fentanyl | 0.02 mg kg^-1^  0.5 mg kg^-1^  1.0 μg kg^-1^ | Propofol | 2.0 mg kg^-1^ h^-1^ | Propofol  Fentanyl | 0.25 mg kg^-1^  0.5 μg kg^-1^ |
| Singh 2022 | Ketamine  Dexmedetomidine  Midazolam | 0.5 mg kg^-1^  1.0 μg kg^-1^  0.02 mg kg^-1^ | Ketamine  Dexmedetomidine | 0.5 mg kg^-1^ h^-1^  0.5 μg kg^-1^ h^-1^ | Ketamine | 10.0 mg |
|  | Ketamine  Midazolam  Propofol | 0.5 mg kg^-1^  0.02 mg kg^-1^  1.0 mg kg^-1^ | Ketamine  Propofol | 0.5 mg kg^-1^ h^-1^  2.0 mg kg^-1^ h^-1^ | Ketamine | 10.0 mg |
| Singh 2023 | Ketamine  Etomidate  Midazolam | 1.0 mg kg^-1^  0.15 mg kg^-1^  1.0 mg | Etomidate | 0.01 mg kg^-1^ min^-1^ | Ketamine | 0.5 mg kg^-1^ |
|  | Ketamine  Dexmedetomidine  Midazolam | 1.0 mg kg^-1^  1.0 μg kg^-1^  1.0 mg | Dexmedetomidine | 0.5 μg kg^-1^ h^-1^ | Ketamine | 0.5 mg kg^-1^ |
| Soliman 2024 | Propofol | 1.0–2.0 mg kg^-1^ | Propofol | 0.05–1.0 μg kg^-1^ h^-1^ | Fentanyl | 1.0 μg kg^-1^ |
|  | Dexmedetomidine | 1.0 μg kg^-1^ | Dexmedetomidine | 0.2–0.7 μg kg^-1^ h^-1^ | Fentanyl | 1.0 μg kg^-1^ |
| Srivastava 2018 | Propofol  Fentanyl | 1.0–2.0 mg kg^-1^  1.0 μg kg^-1^ | Propofol | 1.0–5.0 mg kg^-1^ h^-1^ | Propofol | 10.0–20.0 mg |
|  | Midazolam  Propofol | 0.04 mg kg^-1^  1.0–2.0 mg kg^-1^ | Propofol | 1.0–5.0 mg kg^-1^ h^-1^ | Propofol | 10.0–20.0 mg |
|  | Dexmedetomidine  Propofol | 1.0 μg kg^-1^  1.0–2.0 mg kg^-1^ | Propofol | 1.0–5.0 mg kg^-1^ h^-1^ | Propofol | 10.0–20.0 mg |
| Srivastava 2021 | Propofol  Fentanyl | 25.0–75.0 μg kg^-1^ min^-1^  1.0 μg kg^-1^ | Propofol | 25.0–75.0 μg kg^-1^ min^-1^ | Propofol | 10.0 mg |
|  | Dexmedetomidine  Fentanyl | 1.0 μg kg^-1^  1.0 μg kg^-1^ | Dexmedetomidine | 0.5 μg kg^-1^ h^-1^ | Propofol | 10.0 mg |
| Sun 2024 | Propofol  Nalbuphine | 1.5 mg kg^-1^  1.5–2.0 mg kg^-1^ | Propofol | 1.0–2.0 mg kg^-1^ h^-1^ | Propofol | 0.5 mg kg^-1^ |
|  | Dexmedetomidine  Propofol  Nalbuphine | 0.4 μg kg^-1^ h^-1^  1.5 mg kg^-1^  1.5–2.0 mg kg^-1^ | Dexmedetomidine  Propofol | 0.4 μg kg^-1^ h^-1^  1.0–2.0 mg kg^-1^ h^-1^ | Propofol | 0.5 mg kg^-1^ |
| Takimoto 2011 | Dexmedetomidine  Pentazocine | 3.0 μg kg^-1^  7.5 mg | Dexmedetomidine | 0.4 μg kg^-1^ h^-1^ | Midazolam | 1.0 mg |
|  | Propofol  Pentazocine | 5.0 mg  7.5 mg | Propofol | 3.0 mg kg^-1^ h^-1^ | Midazolam | 1.0 mg |
|  | Midazolam  Pentazocine | 0.1 mg kg^-1^  7.5 mg | Midazolam | 1.0 mg | Midazolam | 1.0 mg |
| Tian 2024 | Propofol  Sufentanil | 1.5 mg kg^-1^  5.0 μg | Propofol  Remifentanil | NA | Propofol | 0.5 mg kg^-1^ |
|  | Remimazolam  Sufentanil | 0.2 mg kg^-1^  5.0 μg | Remimazolam  Remifentanil | NA | Remimazolam | 2.5 mg |
| Wang 2024 | Dexmedetomidine  Propofol  Sufentanil | 0.15 μg kg^-1^  1.0 mg kg^-1^  0.2 μg kg^-1^ | Propofol | 4.0–6.0 mg kg^-1^ h^-1^ | Propofol | 0.5 mg kg^-1^ |
|  | Dexmedetomidine  Ciprofol  Sufentanil | 0.15 μg kg^-1^  0.2 mg kg^-1^  0.2 μg kg^-1^ | Ciprofol | 0.6–0.8 mg kg^-1^ h^-1^ | Ciprofol | 0.1 mg kg^-1^ |
| Wehrmann 1999 | Propofol | 40.0–60.0 mg | Propofol | 20.0 mg | Propofol | 20.0 mg |
|  | Midazolam  Pentazocine | 2.5 mg  30.0 mg | Midazolam | 2.5 mg | Midazolam | 2.5 mg |
| Xiao 2024 | Remimazolam  Remifentanil | 0.2 mg kg^-1^  2.0 μg kg^-1^ | Remimazolam  Remifentanil | 0.8 mg kg^-1^ h^-1^  0.15 μg kg^-1^ h^-1^ | Remimazolam  Remifentanil | 5.0 mg  25.0 μg |
|  | Propofol  Remifentanil | 1.5 mg kg^-1^  2.0 μg kg^-1^ | Propofol  Remifentanil | 4.0 mg kg^-1^ h^-1^  0.15 μg kg^-1^ h^-1^ | Propofol  Remifentanil | 50.0 mg  25.0 μg |
| Xin 2024 | Remimazolam  Alfentanil | 0.15–0.2 mg kg^-1^  5.0 μg kg^-1^ | Remifentanil  Alfentanil | 0.4–0.8 mg kg^-1^ h^-1^  0.5 μg kg^-1^ min^-1^ | Remifentanil | 0.05 mg kg^-1^ |
|  | Propofol  Alfentanil | 1.0–1.5 mg kg^-1^  5.0 μg kg^-1^ | Propofol  Alfentanil | 2.0–6.0 mg kg^-1^ h^-1^  0.5 μg/ kg^-1^ min^-1^ | Propofol | 0.5 mg kg^-1^ |
| Yüksel 2007 | Midazolam | 2.0–3.0 mg | Midazolam | 1.0 mg | Midazolam | 1.0 mg |
|  | Midazolam  Pethidine | 1.0–2.0 mg  25.0–50.0 mg | Midazolam  Pethidine | 1.0 mg  25.0 mg | Midazolam  Pethidine | 1.0 mg  25.0 mg |
| Zhang 2024 | Dexmedetomidine  Midazolam  Sufentanil | 0.6 μg kg^-1^  0.02 mg kg^-1^  0.2 μg kg^-1^ | Dexmedetomidine | 1.2 μg kg^-1^ h^-1^ | Midazolam  Sufentanil | 0.01 mg kg^-1^  0.1 μg kg^-1^ |
|  | Midazolam  Propofol  Sufentanil | 0.02 mg kg^-1^  1.0–2.0 mg kg^-1^  0.2 μg kg^-1^ | Propofol | 2.0–3.0 mg kg^-1^ h^-1^ | Midazolam  Sufentanil | 0.01 mg kg^-1^  0.1 μg kg^-1^ |
| Zhou 2024 | Remimazolam  Remifentanil  Butorphanol | 0.2–0.4 mg kg^-1^  0.3 μg kg^-1^ h^-1^  0.02 mg kg^-1^ | Remimazolam | 0.2–0.4 mg kg^-1^ h^-1^ | Remimazolam | 3.0–6.0 mg |
|  | Propofol  Remifentanil  Butorphanol | 1.0–2.5 mg kg^-1^  0.3 μg kg^-1^ h^-1^  0.02 mg kg^-1^ | Propofol | 2.0–4.0 mg kg^-1^ h^-1^ | Propofol | 20.0–50.0 mg |

**Table 8** **Risk of bias assessment for included studies using the Cochrane RoB 2.0 Tool**

| **Study (Author, Year)** | **D1** | **D2** | **D3** | **D4** | **D5** | **Overall** |
| --- | --- | --- | --- | --- | --- | --- |
| Amir 2024 | Unclear | High | Low | Low | Unclear | High |
| Angsuwatcharakon 2012 | Low | Low | Low | Low | Low | Low |
| Aqeel 2024 | Unclear | Unclear | Low | Unclear | Unclear | Unclear |
| Ashikari 2021 | Low | Low | Low | Low | Low | Low |
| Ates 2021 | Unclear | Low | Low | Low | Unclear | Unclear |
| Babu 2024 | Low | Low | Low | Low | Unclear | Unclear |
| Bahrami 2016 | Unclear | Unclear | Unclear | Unclear | Low | Unclear |
| Chen 2022 | Low | Low | Low | Low | Low | Low |
| Chun 2012 | Unclear | Unclear | Low | High | Unclear | High |
| Dhingra 2023 | Low | High | Low | Unclear | Low | High |
| Ding 2024 | Low | Low | Low | Low | Low | Low |
| Dong 2023 | Low | Low | Low | Low | Low | Low |
| Eberl 2020 | Low | Low | Low | Low | Low | Low |
| Fabbri 2012 | Low | Low | Low | Low | Unclear | Unclear |
| Gao 2025 | Low | Low | Low | Low | Low | Low |
| Garg 2019 | Low | Low | Low | Unclear | Unclear | Unclear |
| Goyal 2016 | Low | High | Unclear | Low | Low | High |
| Han 2017 | Low | Low | Low | Low | Low | Low |
| Han 2019 | Low | Low | Unclear | Low | Unclear | Unclear |
| Hasanein 2013 | Low | Low | Low | Low | Unclear | Unclear |
| Haytural 2015 | Unclear | High | Low | Unclear | Unclear | High |
| Heidari 2014 | Unclear | Low | High | Low | High | High |
| Jung 2000 | Unclear | High | Low | High | Unclear | High |
| Kilic 2011 | Unclear | Unclear | Low | High | Unclear | High |
| Kim 2015 | Unclear | High | Low | High | Low | High |
| Kim 2016 | Low | Low | Low | Low | Low | Low |
| Koruk 2020 | Low | Low | High | Low | Unclear | High |
| Lee 2011 | Low | High | Low | Unclear | Unclear | High |
| Lee 2012 | Low | Low | Low | Low | Low | Low |
| Lee 2014 | Low | Low | Unclear | Low | Low | Unclear |
| Lee 2023 | Unclear | High | Low | High | Unclear | High |
| Lee SJ 2015 | Low | Low | Low | Low | Low | Low |
| Lee SP 2015 | Low | Low | Low | Low | Low | Low |
| Liu 2020 | Low | Low | Low | Low | Low | Low |
| Lu 2018 | Low | Low | Low | High | Low | High |
| Mukhopadhyay 2015 | Unclear | High | Low | Low | Unclear | High |
| Muller 2008 | Low | Low | Low | Low | High | High |
| Narayanan 2015 | Low | High | Low | Unclear | Low | High |
| Nonaka 2018 | Low | High | Low | High | Low | High |
| Pushkarna 2019 | Unclear | Unclear | Low | Low | Low | Unclear |
| Ramkiran 2015 | Unclear | Low | Low | Low | Unclear | Unclear |
| Riphaus 2005 | Low | Low | Low | Unclear | Unclear | Unclear |
| Sasaki 2012 | Low | High | Low | High | Low | High |
| Sethi 2014 | Low | High | Low | High | Unclear | High |
| Shin 2017 | Low | Low | Low | Low | Low | Low |
| Singh 2022 | Low | High | Low | Unclear | Low | High |
| Singh 2023 | Low | Low | Low | Low | Unclear | Unclear |
| Soliman 2024 | Low | High | Low | Unclear | Low | High |
| Srivastava 2018 | Low | Low | Unclear | Unclear | Low | Unclear |
| Srivastava 2021 | Low | High | Low | Unclear | Low | High |
| Sun 2024 | Low | Low | Low | Low | Low | Low |
| Takimoto 2011 | Low | Low | Low | Low | Unclear | Unclear |
| Tian 2024 | Unclear | Unclear | Low | Unclear | Low | Unclear |
| Wang 2024 | Unclear | High | Low | Unclear | Low | High |
| Wehrmann 1999 | Unclear | High | Low | Unclear | Unclear | High |
| Xiao 2024 | Low | Low | Low | Low | Low | Low |
| Xin 2024 | Unclear | High | Low | High | Low | High |
| Yüksel 2007 | Unclear | Unclear | Low | Low | Unclear | Unclear |
| Zhang 2024 | Unclear | Unclear | Low | High | Low | High |
| Zhou 2024 | Unclear | Unclear | Low | Unclear | Low | Unclear |

**Figure 1 Risk of bias across RoB 2.0 domains**

**
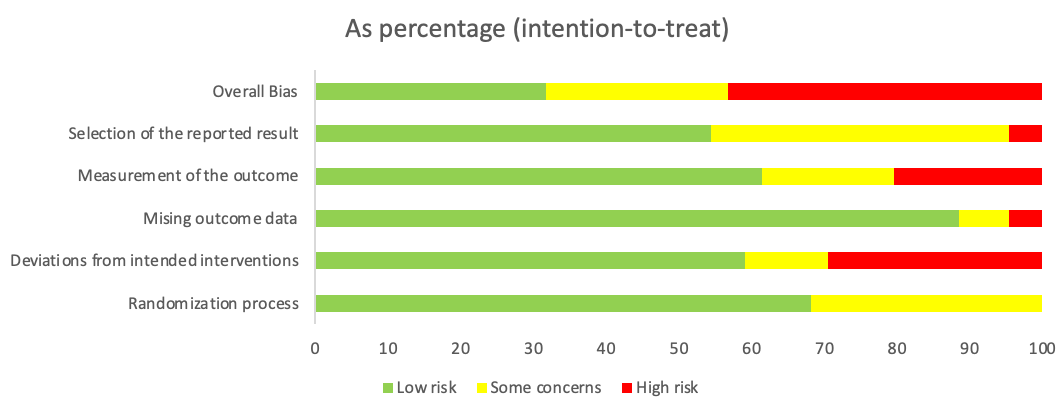
**

**Figure 2 Comparison-adjusted funnel plot for procedural interference events**

**Figure 3 Comparison-adjusted funnel plot for hypoxia**

**Figure 4 Comparison-adjusted funnel plot for hypotension**

**Figure 5 Comparison-adjusted funnel plot for bradycardia**

**Figure 6 Comparison-adjusted funnel plot for recovery time**

**Table 9 Egger’s test**

| **Egger’s test for procedural interference events** | | | | | |
| --- | --- | --- | --- | --- | --- |
| Std_Eff | Coef. | Std. Err. | *t*-value | *P*-value | 95% Conf. Interval |
| slope | 0.791 | 0.217 | 0.364 | 0.718 | -0.365 to 0.523 |
| bias | 0.805 | 0.564 | 1.429 | 0.163 | -0.346 to 1.956 |
| **Egger’s test for hypoxia** | | | | | |
| Std_Eff | Coef. | Std. Err. | *t*-value | *P*-value | 95% Conf. Interval |
| slope | -0.040 | 0.173 | -0.231 | 0.816 | -0.387 to 0.307 |
| bias | 0.884 | 0.331 | 2.667 | 0.010 | 0.219 to 1.548 |
| **Egger’s test for hypotension** | | | | | |
| Std_Eff | Coef. | Std. Err. | *t*-value | *P*-value | 95% Conf. Interval |
| slope | -0.349 | 0.158 | -2.212 | 0.032 | -0.666 to -0.032 |
| bias | 0.584 | 0.307 | 1.903 | 0.063 | -0.032 to 1.200 |
| **Egger’s test for bradycardia** | | | | | |
| Std_Eff | Coef. | Std. Err. | *t*-value | *P*-value | 95% Conf. Interval |
| slope | -0.777 | 0.237 | -3.279 | 0.002 | -1.258 to -2.959 |
| bias | 0.520 | 0.306 | 1.696 | 0.099 | -0.103 to 1.142 |
| **Egger’s test for recovery time** | | | | | |
| Std_Eff | Coef. | Std. Err. | *t*-value | *P*-value | 95% Conf. Interval |
| slope | 0.328 | 0.373 | 0.878 | 0.384 | -0.422 to 1.077 |
| bias | -2.051 | 1.959 | -1.047 | 0.300 | -5.986 to 1.884 |

**Table 10 SUCRA rankings for procedural interference events**

| Treatment | SUCRA | PrBest | MeanRank |
| --- | --- | --- | --- |
| PF-OP | 49.0 | 0 | 9.7 |
| DEX-CFL-OP | 78.8 | 14.3 | 4.6 |
| DEX-MDZ-OP | 33.3 | 0 | 12.3 |
| DEX-OP | 42.1 | 0.1 | 10.8 |
| DEX-PF | 70.1 | 1.8 | 6.1 |
| DEX-PF-OP | 74.8 | 4.1 | 5.3 |
| ETM-MDZ-OP | 63.5 | 0.3 | 7.2 |
| KET-OP | 33.0 | 0 | 12.4 |
| KET-PF | 61.1 | 1.1 | 7.6 |
| LIDO-MDZ-PF | 57.2 | 1.0 | 8.3 |
| LIDO-MDZ-PF-OP | 86.3 | 22.4 | 3.3 |
| LIDO-PF-OP | 91.8 | 53.9 | 2.4 |
| MDZ | 28.8 | 1.0 | 13.1 |
| MDZ-OP | 8.1 | 0 | 16.6 |
| MDZ-PF | 22.8 | 0 | 14.1 |
| MDZ-PF-OP | 57.6 | 0 | 8.2 |
| PF | 16.6 | 0 | 15.2 |
| RMZ-OP | 25.3 | 0 | 13.7 |

**Table 11 Pairwise comparisons for procedural interference events**

| **Treatment/Comparison** | **Direct Coef.** | **Std. Err.** | **Indirect Coef.** | **Std. Err.** | **Difference Coef.** | **Std. Err.** | **p value** | **Tau** |
| --- | --- | --- | --- | --- | --- | --- | --- | --- |
| PF-OP vs RMZ-OP | – | – | – | – | – | – | – | – |
| DEX-CFL-OP vs DEX-PF-OP* | 0.170 | 0.393 | -1.825 | 129.429 | 1.995 | 129.429 | 0.988 | 0.315 |
| DEX-MDZ-OP vs MDZ-OP* | 0.720 | 0.262 | 2.300 | 36.278 | -1.579 | 36.279 | 0.965 | 0.315 |
| DEX-OP vs PF-OP | -0.628 | 0.470 | 0.474 | 0.525 | -1.102 | 0.709 | 0.120 | 0.234 |
| DEX-OP vs MDZ-OP | 1.202 | 0.316 | -0.228 | 0.651 | 1.429 | 0.715 | 0.046 | 0.226 |
| DEX-PF vs PF-OP* | 0.223 | 0.703 | 1.858 | 1.395 | -1.634 | 1.593 | 0.305 | 0.318 |
| DEX-PF vs MDZ-PF* | 1.233 | 0.377 | 1.443 | 74.601 | -0.210 | 74.602 | 0.998 | 0.315 |
| DEX-PF vs PF* | 1.602 | 0.652 | 0.590 | 105.082 | 1.011 | 105.087 | 0.992 | 0.315 |
| DEX-PF-OP vs PF-OP* | 0.916 | 0.866 | -0.081 | 64.708 | 0.998 | 64.713 | 0.988 | 0.315 |
| ETM-MDZ-OP vs MDZ-PF-OP* | 0.152 | 0.321 | -0.467 | 70.063 | 0.621 | 70.064 | 0.993 | 0.315 |
| KET-OP vs PF-OP | – | – | – | – | – | – | – | – |
| KET-PF vs PF-OP | – | – | – | – | – | – | – | – |
| LIDO-MDZ-PF vs MDZ-PF* | 0.916 | 0.532 | 1.521 | 106.016 | -0.604 | 106.016 | 0.995 | 0.315 |
| LIDO-MDZ-PF-OP vs MDZ-PF-OP* | 1.204 | 0.670 | -0.130 | 103.773 | 1.334 | 103.773 | 0.990 | 0.315 |
| LIDO-PF-OP vs PF-OP | – | – | – | – | – | – | – | – |
| MDZ vs MDZ-OP* | 0.405 | 0.944 | 2.225 | 269.824 | -1.819 | 269.825 | 0.995 | 0.315 |
| MDZ-OP vs PF-OP | -0.786 | 0.340 | -2.004 | 0.618 | 1.218 | 0.704 | 0.084 | 0.231 |
| MDZ-PF vs PF-OP* | -0.470 | 0.607 | -2.100 | 1.522 | 1.630 | 1.592 | 0.306 | 0.318 |
| MDZ-PF-OP vs PF-OP* | 0.236 | 0.404 | -0.186 | 29.027 | 0.422 | 29.030 | 0.988 | 0.315 |
| **** Warning: all the evidence about these contrasts comes from the trials which directly compare them.*** | | | | | | | | |

**Table 12 Assessment of local heterogeneity for procedural interference events**

| **Loop** | | | **ROR** | **z_value** | **p_value** | **RoR 95% CI** | **Loop Heterogeneity (tau2)** |
| --- | --- | --- | --- | --- | --- | --- | --- |
| DEX-OP | MDZ-OP | PF-OP | 2.560 | 1.163 | 0.245 | (1.00, 12.48) | 0.138 |
| DEX-PF | MDZ-PF | PF-OP | 2.229 | 0.901 | 0.367 | (1.00, 12.74) | 0.000 |

**Figure 7 Cumulative ranking curves for procedural interference events**

**Table 13 SUCRA rankings for hypoxia**

| Treatment | SUCRA | PrBest | MeanRank |
| --- | --- | --- | --- |
| PF-OP | 14.2 | 0 | 25.0 |
| CFL-OP | 41.1 | 0 | 17.5 |
| DEX | 57.1 | 0 | 13.0 |
| DEX-CFL-OP | 71.2 | 2.6 | 9.1 |
| DEX-MDZ | 56.4 | 12.4 | 13.2 |
| DEX-MDZ-OP | 65.8 | 0.2 | 10.6 |
| DEX-MDZ-PF | 54.4 | 6.1 | 13.8 |
| DEX-OP | 70.2 | 1.2 | 9.3 |
| DEX-PF | 58.3 | 0 | 12.7 |
| DEX-PF-OP | 27.3 | 0 | 21.4 |
| ESK-PF | 11.9 | 0 | 25.7 |
| ETM-MDZ-OP | 64.4 | 2.4 | 11.0 |
| KET-DEX | 90.1 | 46.2 | 3.8 |
| KET-DEX-MDZ | 66.5 | 1.9 | 10.4 |
| KET-ETM-MDZ | 71.7 | 9.8 | 8.9 |
| KET-MDZ-PF | 61.1 | 0.1 | 11.9 |
| KET-OP | 60.2 | 0.2 | 12.1 |
| KET-PF | 76.7 | 2.8 | 7.5 |
| KET-PF-OP | 48.9 | 0 | 15.3 |
| LIDO-MDZ-PF | 87.3 | 14.1 | 4.6 |
| LIDO-MDZ-PF-OP | 35.3 | 0 | 19.1 |
| LIDO-PF-OP | 29.5 | 0 | 20.7 |
| MDZ | 17.1 | 0 | 24.2 |
| MDZ-OP | 24.2 | 0 | 22.2 |
| MDZ-PF | 58.2 | 0 | 12.7 |
| MDZ-PF-OP | 14.1 | 0 | 25.0 |
| PF | 36.4 | 0 | 18.8 |
| RMZ | 38.6 | 0 | 18.2 |
| RMZ-OP | 41.7 | 0 | 17.3 |

**Table 14 Pairwise comparisons for hypoxia**

| **Treatment/Comparison** | **Direct Coef.** | **Std. Err.** | **Indirect Coef.** | **Std. Err.** | **Difference Coef.** | **Std. Err.** | **p value** | **Tau** |
| --- | --- | --- | --- | --- | --- | --- | --- | --- |
| PF-OP vs RMZ-OP | – | – | – | – | – | – | – | – |
| CFL-OP vs PF-OP | – | – | – | – | – | – | – | – |
| DEX vs PF* | 0.588 | 0.459 | -1.206 | 123.438 | 1.794 | 123.439 | 0.988 | 0.356 |
| DEX-CFL-OP vs DEX-PF-OP* | 1.540 | 0.720 | -0.113 | 260.740 | 1.653 | 260.740 | 0.995 | 0.356 |
| DEX-MDZ vs KET-MDZ-PF* | 1.15×10^-11^ | 2.016 | -3.102 | 501.872 | 3.102 | 501.876 | 0.995 | 0.356 |
| DEX-MDZ-OP vs MDZ-OP | 1.152 | 0.502 | 2.263 | 1.510 | -1.111 | 1.580 | 0.482 | 0.354 |
| DEX-MDZ-OP vs MDZ-PF-OP | 2.518 | 1.479 | 1.407 | 0.557 | 1.112 | 1.580 | 0.482 | 0.354 |
| DEX-MDZ-PF vs KET-MDZ-PF* | -2.33×10^-12^ | 2.013 | -0.606 | 4.391 | 0.606 | 4.831 | 0.900 | 0.365 |
| DEX-MDZ-PF vs MDZ-PF* | -2.13×10^-12^ | 2.013 | 0.606 | 4.391 | -0.606 | 4.831 | 0.900 | 0.365 |
| DEX-OP vs PF-OP | 1.752 | 0.703 | 1.618 | 1.177 | 0.134 | 1.195 | 0.911 | 0.381 |
| DEX-OP vs MDZ-OP | 1.529 | 0.961 | 1.353 | 0.856 | 0.177 | 1.156 | 0.879 | 0.388 |
| DEX-PF vs MDZ-PF | 0.199 | 0.657 | -0.754 | 1.113 | 0.954 | 1.293 | 0.461 | 0.365 |
| DEX-PF vs PF | 0.562 | 0.395 | 1.514 | 1.231 | -0.952 | 1.292 | 0.461 | 0.365 |
| DEX-PF-OP vs PF-OP* | 0.328 | 0.565 | -0.499 | 130.374 | 0.827 | 130.355 | 0.995 | 0.356 |
| ESK-PF vs PF-OP | – | – | – | – | – | – | – | – |
| ETM-MDZ-OP vs MDZ-PF-OP* | 1.657 | 0.963 | 0.520 | 178.816 | 1.137 | 178.840 | 0.995 | 0.356 |
| KET-DEX vs PF-OP | – | – | – | – | – | – | – | – |
| KET-DEX-MDZ vs KET-ETM-MDZ* | -0.405 | 0.945 | 3.637 | 209.816 | -4.042 | 209.817 | 0.985 | 0.356 |
| KET-DEX-MDZ vs KET-MDZ-PF* | 0.288 | 0.609 | -2.212 | 84.257 | 2.500 | 84.263 | 0.976 | 0.356 |
| KET-MDZ-PF vs MDZ-PF* | 1.56×10^-12^ | 2.013 | 0.303 | 1.335 | -0.303 | 2.415 | 0.900 | 0.365 |
| KET-MDZ-PF vs MDZ-PF-OP | 1.609 | 1.104 | 1.304 | 2.147 | 0.306 | 2.414 | 0.899 | 0.365 |
| KET-OP vs PF-OP | – | – | – | – | – | – | – | – |
| KET-PF vs PF-OP | 2.708 | 1.499 | 1.847 | 0.949 | 0.861 | 1.774 | 0.627 | 0.365 |
| KET-PF vs PF | 1.253 | 0.842 | 2.114 | 1.562 | -0.861 | 1.774 | 0.628 | 0.365 |
| KET-PF-OP vs PF-OP | – | – | – | – | – | – | – | – |
| LIDO-MDZ-PF vs MDZ-PF* | 1.609 | 0.823 | -2.095 | 150.549 | 3.704 | 150.548 | 0.980 | 0.356 |
| LIDO-MDZ-PF-OP vs MDZ-PF-OP* | 0.610 | 0.430 | 0.774 | 107.120 | 0.532 | 107.121 | 0.996 | 0.356 |
| LIDO-PF-OP vs PF-OP | – | – | – | – | – | – | – | – |
| MDZ vs MDZ-OP* | -0.318 | 0.549 | -0.567 | 127.308 | 0.249 | 127.310 | 0.998 | 0.356 |
| MDZ-OP vs PF-OP | 0.159 | 0.314 | 0.806 | 0.622 | -0.647 | 0.702 | 0.357 | 0.322 |
| MDZ-OP vs MDZ-PF-OP | 0.626 | 0.201 | -0.433 | 0.428 | 1.059 | 0.471 | 0.024 | 0.123 |
| MDZ-OP vs PF | -0.576 | 0.350 | 0.336 | 0.626 | -0.912 | 0.720 | 0.205 | 0.286 |
| MDZ-PF vs PF | 1.401 | 1.162 | 0.422 | 0.727 | 0.979 | 1.371 | 0.475 | 0.361 |
| MDZ-PF-OP vs PF-OP | 0.625 | 0.633 | -0.294 | 0.423 | 0.919 | 0.763 | 0.229 | 0.306 |
| MDZ-PF-OP vs PF | 0.135 | 0.595 | -1.089 | 0.420 | 1.224 | 0.729 | 0.093 | 0.253 |
| PF vs RMZ* | -2.86×10^-13^ | 0.870 | 1.292 | 263.304 | -1.292 | 263.305 | 0.996 | 0.356 |
| **** Warning: all the evidence about these contrasts comes from the trials which directly compare them.*** | | | | | | | | |

**Table 15 Assessment of local heterogeneity for hypoxia**

| **Loop** | | | | **ROR** | **z_value** | **p_value** | **RoR 95% CI** | **Loop Heterogeneity (tau2)** |
| --- | --- | --- | --- | --- | --- | --- | --- | --- |
| MDZ-OP | MDZ-PF-OP | PF |  | 3.855 | 2.142 | 0.032 | (1.12, 13.25) | 0.000 |
| MDZ-OP | MDZ-PF-OP | PF-OP |  | 3.155 | 1.801 | 0.072 | (1.00, 11.02) | 0.000 |
| DEX-PF | MDZ-PF | PF |  | 2.847 | 0.819 | 0.413 | (1.00, 34.79) | 0.000 |
| KET-PF | MDZ-PF-OP | PF | PF-OP | 2.594 | 0.525 | 0.600 | (1.00, 91.33) | 0.000 |
| DEX-OP | MDZ-OP | PF-OP |  | 2.561 | 0.698 | 0.485 | (1.00, 35.86) | 0.000 |
| KET-PF | MDZ-OP | PF | PF-OP | 2.124 | 0.448 | 0.654 | (1.00, 57.36) | 0.000 |
| KET-MDZ-PF | MDZ-PF | MDZ-PF-OP | PF | 1.409 | 0.134 | 0.893 | (1.00, 209.82) | 0.000 |
| DEX-MDZ-OP | MDZ-OP | MDZ-PF-OP |  | 1.335 | 0.135 | 0.893 | (1.00, 88.37) | 0.696 |
| **** Note: Loop (DEX-MDZ-PF, KET-MDZ-PF, MDZ-PF) is formed only by multi-arm trial(s) - Consistent by definition* | | | | | | | | |

**Figure 8 Network Plot for hypoxia**

**Figure 9 Cumulative ranking curves for hypoxia**

**Table 16 SUCRA rankings for hypotension**

| Treatment | SUCRA | PrBest | MeanRank |
| --- | --- | --- | --- |
| PF-OP | 39.5 | 0 | 14.3 |
| CFL-OP | 45.6 | 0 | 13.0 |
| DEX | 27.1 | 0 | 17.0 |
| DEX-MDZ | 49.5 | 12.7 | 12.1 |
| DEX-MDZ-OP | 21.5 | 0 | 18.3 |
| DEX-MDZ-PF | 51.9 | 5.9 | 11.6 |
| DEX-OP | 38.5 | 0 | 14.5 |
| DEX-PF | 23.1 | 0 | 17.9 |
| DEX-PF-OP | 58.4 | 0.2 | 10.1 |
| ESK-PF | 62.4 | 0.1 | 9.3 |
| ETM-MDZ-OP | 62.6 | 0.4 | 9.2 |
| KET-DEX | 90.4 | 38.6 | 3.1 |
| KET-MDZ-PF | 49.9 | 3.5 | 12.0 |
| KET-PF | 80.8 | 0.7 | 5.2 |
| LIDO-MDZ-PF | 89.6 | 27.9 | 3.3 |
| LIDO-MDZ-PF-OP | 34.2 | 0 | 15.5 |
| MDZ | 58.4 | 0 | 10.2 |
| MDZ-OP | 30.6 | 0 | 16.3 |
| MDZ-PF | 57.0 | 0 | 10.5 |
| MDZ-PF-OP | 9.3 | 0 | 20.9 |
| PF | 36.3 | 0 | 15.0 |
| RMZ | 62.9 | 10.0 | 9.2 |
| RMZ-OP | 70.3 | 0 | 7.5 |

**Table 17 Pairwise comparisons for hypotension**

| **Treatment/Comparison** | **Direct Coef.** | **Std. Err.** | **Indirect Coef.** | **Std. Err.** | **Difference Coef.** | **Std. Err.** | **p value** | **Tau** |
| --- | --- | --- | --- | --- | --- | --- | --- | --- |
| PF-OP vs RMZ-OP | – | – | – | – | – | – | – | – |
| CFL-OP vs PF-OP | – | – | – | – | – | – | – | – |
| DEX vs PF* | -0.251 | 0.413 | 0.234 | 145.817 | -0.485 | 145.817 | 0.997 | 0.252 |
| DEX-MDZ vs KET-MDZ-PF* | 1.77×10^-11^ | 2.000 | -0.861 | 502.110 | 0.861 | 502.114 | 0.999 | 0.252 |
| DEX-MDZ-OP vs MDZ-OP | -0.262 | 0.776 | -0.392 | 0.700 | 0.130 | 1.045 | 0.901 | 0.272 |
| DEX-MDZ-OP vs MDZ-PF-OP | 0.288 | 0.565 | 0.418 | 0.879 | -0.130 | 1.045 | 0.901 | 0.272 |
| DEX-MDZ-PF vs KET-MDZ-PF* | 6.33×10^-12^ | 1.996 | 0.861 | 501.918 | -0.861 | 501.922 | 0.999 | 0.252 |
| DEX-MDZ-PF vs MDZ-PF* | 2.74×10^-13^ | 1.996 | -0.861 | 332.006 | 0.861 | 332.012 | 0.998 | 0.252 |
| DEX-OP vs PF-OP | 0.245 | 0.482 | -1.327 | 0.975 | 1.572 | 1.064 | 0.140 | 0.122 |
| DEX-OP vs MDZ-OP | -0.137 | 0.586 | 0.904 | 0.949 | -1.041 | 1.135 | 0.359 | 0.181 |
| DEX-PF vs PF-OP | -1.649 | 1.479 | -0.260 | 0.610 | -1.390 | 11.580 | 0.379 | 0.258 |
| DEX-PF vs MDZ-PF | -1.148 | 0.825 | -7.699 | 0.540 | -3.783 | 0.982 | 0.700 | 0.270 |
| DEX-PF vs PF | -0.280 | 0.319 | -0.778 | 0.843 | 0.498 | 0.900 | 0.580 | 0.269 |
| DEX-PF-OP vs PF-OP | – | – | – | – | – | – | – | – |
| ESK-PF vs PF-OP | – | – | – | – | – | – | – | – |
| ETM-MDZ-OP vs MDZ-PF-OP* | 1.572 | 0.710 | 2.290 | 169.996 | -0.718 | 169.996 | 0.997 | 0.252 |
| KET-DEX vs PF-OP | – | – | – | – | – | – | – | – |
| KET-MDZ-PF vs MDZ-PF* | -4.97×10^-11^ | 1.996 | -0.535 | 218.357 | 0.535 | 218.366 | 0.998 | 0.252 |
| KET-PF vs PF-OP | 1.386 | 0.685 | 1.076 | 0.992 | 0.310 | 1.205 | 0.797 | 0.269 |
| KET-PF vs PF | 1.253 | 0.805 | 1.563 | 0.896 | -0.310 | 1.205 | 0.797 | 0.269 |
| LIDO-MDZ-PF vs MDZ-PF* | 2.078 | 1.067 | 0.068 | 177.641 | 2.012 | 177.639 | 0.991 | 0.252 |
| LIDO-MDZ-PF-OP vs MDZ-PF-OP* | 0.717 | 0.431 | 1.846 | 116.599 | -1.130 | 116.599 | 0.992 | 0.252 |
| MDZ vs MDZ-OP | 0.606 | 0.531 | 1.248 | 1.712 | -0.642 | 1.792 | 0.720 | 0.257 |
| MDZ vs PF | 1.099 | 1.628 | 0.456 | 0.727 | 0.642 | 1.792 | 0.720 | 0.257 |
| MDZ-OP vs PF-OP | -0.142 | 0.342 | -0.465 | 0.706 | 0.322 | 0.788 | 0.683 | 0.287 |
| MDZ-OP vs MDZ-PF-OP | 0.863 | 0.482 | 0.347 | 0.602 | 0.515 | 0.772 | 0.505 | 0.250 |
| MDZ-OP vs PF | -0.640 | 0.692 | 0.384 | 0.661 | -1.026 | 0.953 | 0.282 | 0.261 |
| MDZ-PF vs PF-OP | 3.46×10^-10^ | 2.003 | 0.479 | 0.664 | -0.479 | 2.110 | 0.820 | 0.259 |
| MDZ-PF vs PF | 0.507 | 0.437 | 0.673 | 0.887 | -0.166 | 0.989 | 0.867 | 0.272 |
| MDZ-PF-OP vs PF-OP | -0.838 | 0.986 | -0.873 | 0.509 | 0.034 | 1.110 | 0.975 | 0.263 |
| MDZ-PF-OP vs PF | -0.019 | 1.020 | -1.067 | 0.653 | 1.048 | 1.211 | 0.387 | 0.243 |
| PF vs RMZ* | -1.099 | 1.641 | -0.506 | 468.973 | -0.593 | 468.973 | 0.999 | 0.252 |
| **** Warning: all the evidence about these contrasts comes from the trials which directly compare them.*** | | | | | | | | |

**Table 18 Assessment of local heterogeneity for hypotension**

| **Loop** | | | | **ROR** | **z_value** | **p_value** | **RoR 95% CI** | **Loop Heterogeneity (tau2)** |
| --- | --- | --- | --- | --- | --- | --- | --- | --- |
| DEX-PF | MDZ-OP | PF | PF-OP | 6.463 | 1.001 | 0.317 | (1.00, 249.36) | 0.225 |
| MDZ-OP | MDZ-PF-OP | PF |  | 4.446 | 1.128 | 0.259 | (1.00, 59.38) | 0.051 |
| DEX-PF | KET-PF | PF | PF-OP | 4.394 | 0.805 | 0.421 | (1.00, 161.50) | 0.000 |
| MDZ | MDZ-OP | PF |  | 3.179 | 0.639 | 0.523 | (1.00, 110.12) | 0.000 |
| MDZ-OP | MDZ-PF | PF | PF-OP | 2.955 | 0.446 | 0.656 | (1.00, 347.12) | 0.456 |
| KET-PF | MDZ-PF-OP | PF | PF-OP | 2.587 | 0.559 | 0.576 | (1.00, 72.31) | 0.000 |
| DEX-OP | MDZ-OP | PF-OP |  | 2.492 | 1.176 | 0.240 | (1.11, 11.41) | 0.000 |
| DEX-PF | MDZ-PF | PF-OP |  | 2.166 | 0.288 | 0.774 | (1.00, 420.86) | 0.000 |
| KET-PF | MDZ-PF | PF | PF-OP | 1.898 | 0.286 | 0.775 | (1.00, 154.22) | 0.000 |
| DEX-PF | MDZ-PF-OP | PF | PF-OP | 1.698 | 0.255 | 0.799 | (1.00, 99.98) | 0.000 |
| KET-PF | MDZ-OP | PF | PF-OP | 1.557 | 0.263 | 0.792 | (1.00, 41.97) | 0.456 |
| DEX-MDZ-OP | MDZ-OP | MDZ-PF-OP |  | 1.381 | 0.323 | 0.747 | (1.00, 9.79) | 0.000 |
| MDZ-PF | MDZ-PF-OP | PF | PF-OP | 1.363 | 0.127 | 0.899 | (1.00, 164.18) | 0.000 |
| DEX-PF | MDZ-PF | PF |  | 1.333 | 0.312 | 0.755 | (1.00, 8.14) | 0.000 |
| MDZ-OP | MDZ-PF-OP | PF-OP |  | 1.113 | 0.090 | 0.928 | (1.00, 11.37) | 0.137 |
| DEX-MDZ-PF | KET-MDZ-PF | MDZ-PF |  | – | – | – | – | 0.000 |
| ****** Note: Loop (DEX-MDZ-PF, KET-MDZ-PF, MDZ-PF) is formed only by multi-arm trial(s) - Consistent by definition*** | | | | | | | | |

**Figure 10 Network Plot for hypotension**

**Figure 11 Cumulative ranking curves for hypotension**

**Table 19 SUCRA rankings for bradycardia**

| Treatment | SUCRA | PrBest | MeanRank |
| --- | --- | --- | --- |
| PF-OP | 42.8 | 0 | 11.9 |
| DEX-MDZ | 41.6 | 10.2 | 12.1 |
| DEX-MDZ-OP | 27.5 | 0 | 14.8 |
| DEX-MDZ-PF | 42.0 | 5.1 | 12.0 |
| DEX-OP | 49.2 | 0.7 | 10.6 |
| DEX-PF | 13.5 | 0 | 17.4 |
| DEX-PF-OP | 15.3 | 0 | 17.1 |
| ESK-PF | 35.5 | 0.3 | 13.3 |
| ETM-MDZ-OP | 78.3 | 9.5 | 5.1 |
| KET-DEX | 73.1 | 14.1 | 6.1 |
| KET-MDZ-PF | 40.3 | 3.0 | 12.3 |
| KET-PF | 85.0 | 21.0 | 3.8 |
| LIDO-MDZ-PF-OP | 79.9 | 23.4 | 4.8 |
| MDZ | 67.3 | 11.0 | 7.2 |
| MDZ-OP | 53.7 | 0 | 9.8 |
| MDZ-PF | 39.6 | 0.9 | 12.5 |
| MDZ-PF-OP | 54.2 | 0 | 9.7 |
| PF | 46.8 | 0 | 11.1 |
| RMZ | 46.9 | 0.8 | 11.1 |
| RMZ-OP | 67.5 | 0 | 7.2 |

**Table 20 Pairwise comparisons for bradycardia**

| **Treatment/Comparison** | **Direct Coef.** | **Std. Err.** | **Indirect Coef.** | **Std. Err.** | **Difference Coef.** | **Std. Err.** | **p value** | **Tau** |
| --- | --- | --- | --- | --- | --- | --- | --- | --- |
| PF-OP vs RMZ-OP | – | – | – | – | – | – | – | – |
| DEX-MDZ vs KET-MDZ-PF* | 4.90×10^-12^ | 1.984 | 0.737 | 502.093 | -0.737 | 502.097 | 0.999 | 2.00×10^-9^ |
| DEX-MDZ-OP vs MDZ-OP | -0.991 | 0.853 | -0.880 | 1.110 | -0.111 | 1.400 | 0.937 | 5.80×10^-11^ |
| DEX-MDZ-OP vs MDZ-PF-OP | -0.965 | 0.776 | -1.076 | 1.165 | 0.111 | 1.400 | 0.937 | 1.00×10^-9^ |
| DEX-MDZ-PF vs KET-MDZ-PF* | -7.20×10^-12^ | 1.980 | -0.737 | 501.781 | 0.737 | 501.784 | 0.999 | 7.23×10^-10^ |
| DEX-MDZ-PF vs MDZ-PF* | 4.71×10^-12^ | 1.980 | 0.737 | 332.009 | -0.737 | 332.015 | 0.998 | 2.44×10^-10^ |
| DEX-OP vs PF-OP | -0.033 | 1.984 | 0.335 | 1.430 | -0.368 | 2.446 | 0.880 | 7.19×10^-9^ |
| DEX-OP vs MDZ-OP | 8.90×10^-12^ | 1.402 | -0.368 | 2.004 | 0.368 | 2.446 | 0.880 | 5.88×10^-9^ |
| DEX-PF vs MDZ-PF* | -1.099 | 1.604 | -1.917 | 143.170 | 0.818 | 143.177 | 0.995 | 4.85×10^-12^ |
| DEX-PF vs PF* | -1.555 | 0.515 | -0.102 | 68.074 | -1.453 | 68.078 | 0.983 | 2.44×10^-8^ |
| DEX-PF-OP vs PF-OP | – | – | – | – | – | – | – | – |
| ESK-PF vs PF-OP | – | – | – | – | – | – | – | – |
| ETM-MDZ-OP vs MDZ-PF-OP* | 1.295 | 0.918 | -0.384 | 276.690 | 1.679 | 276.690 | 0.995 | 3.12×10^-8^ |
| KET-DEX vs PF-OP | – | – | – | – | – | – | – | – |
| KET-MDZ-PF vs MDZ-PF* | 7.75×10^-13^ | 1.980 | 0.458 | 218.346 | -0.458 | 218.355 | 0.998 | 5.35×10^-10^ |
| KET-PF vs PF-OP | – | – | – | – | – | – | – | – |
| LIDO-MDZ-PF-OP vs MDZ-PF-OP* | 1.609 | 1.523 | -0.171 | 250.334 | 1.781 | 250.332 | 0.994 | 2.40×10^-10^ |
| MDZ vs PF* | 1.099 | 1.618 | 0.112 | 399.971 | 0.986 | 399.971 | 0.998 | 9.60×10^-10^ |
| MDZ-OP vs PF-OP | 0.320 | 0.291 | 0.511 | 1.256 | -0.191 | 1.289 | 0.882 | 7.88×10^-10^ |
| MDZ-OP vs MDZ-PF-OP | 0.005 | 1.151 | -0.075 | 0.794 | 0.080 | 1.398 | 0.954 | 1.38×10^-9^ |
| MDZ-OP vs PF | 0.267 | 0.553 | -0.107 | 2.108 | 0.374 | 2.179 | 0.864 | 3.17×10^-10^ |
| MDZ-PF-OP vs PF-OP | 0.658 | 1.258 | 0.264 | 0.811 | 0.394 | 1.497 | 0.792 | 7.54×10^-10^ |
| MDZ-PF-OP vs PF | -0.019 | 1.995 | 0.353 | 0.884 | -0.372 | 2.182 | 0.865 | 3.31×10^-10^ |
| PF vs RMZ* | 1.12×10^-10^ | 0.982 | 0.175 | 325.292 | -0.175 | 325.294 | 1.000 | 1.23×10^-10^ |
| **** Warning: all the evidence about these contrasts comes from the trials which directly compare them.*** | | | | | | | | |

**Table 21 Assessment of local heterogeneity for bradycardia**

| **Loop** | | | **ROR** | **z_value** | **p_value** | **RoR 95% CI** | **Loop Heterogeneity (tau2)** |
| --- | --- | --- | --- | --- | --- | --- | --- |
| DEX-OP | MDZ-OP | PF-OP | 1.424 | 0.144 | 0.885 | (1.00, 172.02) | 0.000 |
| MDZ-OP | MDZ-PF-OP | PF-OP | 1.408 | 0.198 | 0.843 | (1.00, 41.77) | 0.000 |
| MDZ-OP | MDZ-PF-OP | PF | 1.328 | 0.119 | 0.905 | (1.00, 140.97) | 0.027 |
| DEX-MDZ-OP | MDZ-OP | MDZ-PF-OP | 1.021 | 0.013 | 0.990 | (1.00, 24.89) | 0.000 |
| ****** Note: Loop (DEX-MDZ-PF, KET-MDZ-PF, MDZ-PF) is formed only by multi-arm trial(s) - Consistent by definition*** | | | | | | | |

**Figure 12 Network Plot for bradycardia**

*
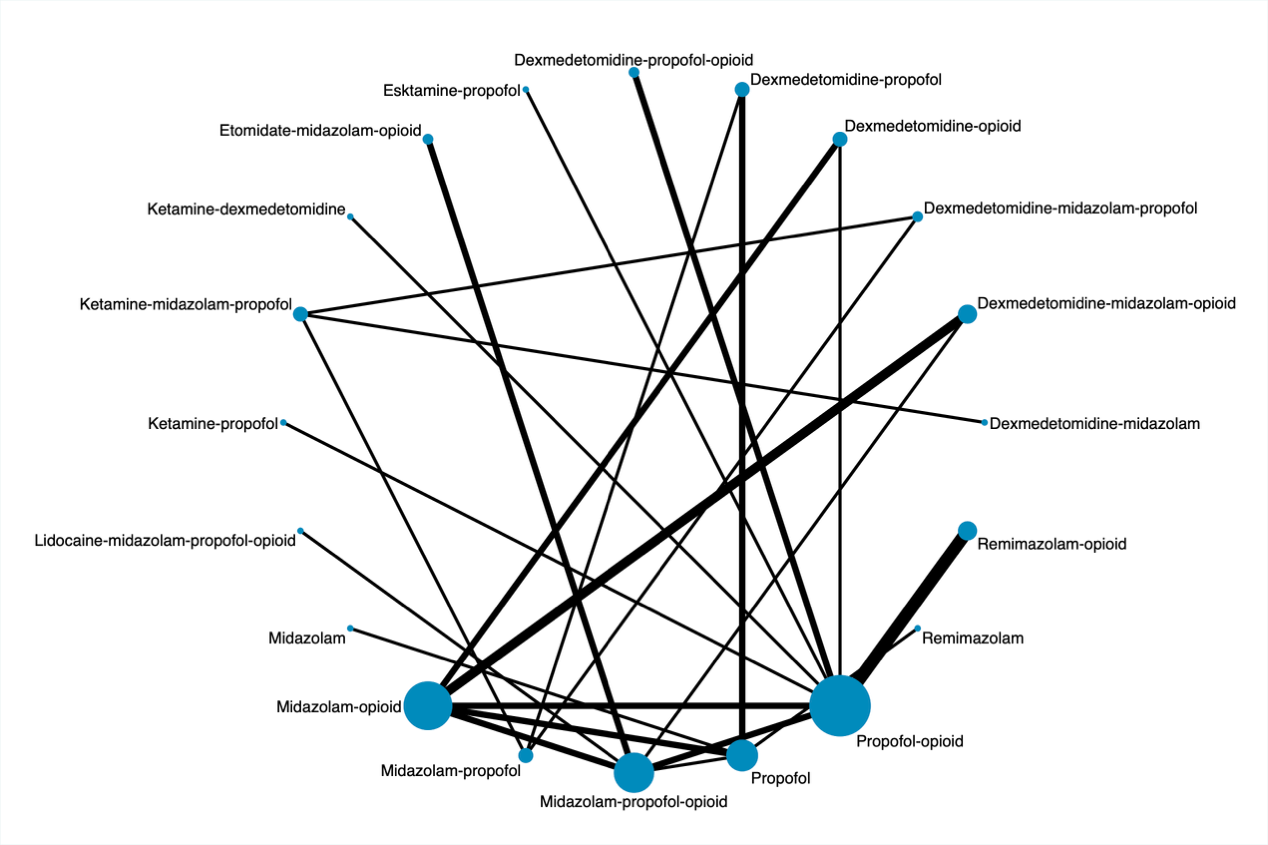
*

**Figure 13 Cumulative ranking curves for bradycardia**

*
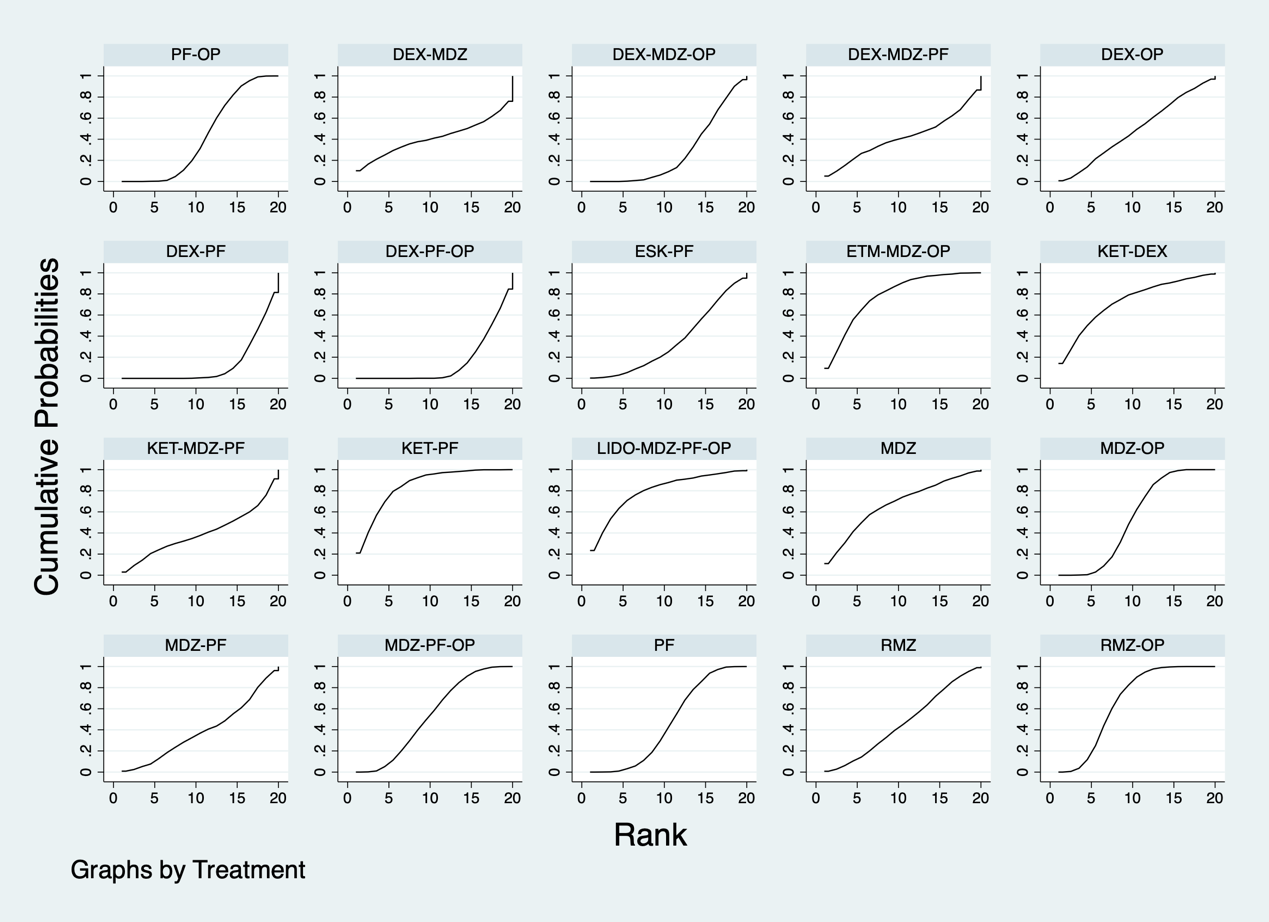
*

**Table 22 SUCRA rankings for recovery time**

| Treatment | SUCRA | PrBest | MeanRank |
| --- | --- | --- | --- |
| PF-OP | 58.4 | 0 | 13.1 |
| CFL-OP | 77.6 | 0.4 | 7.5 |
| DEX | 84.6 | 1.4 | 5.5 |
| DEX-CFL-OP | 32.1 | 0 | 20.7 |
| DEX-MDZ | 42.3 | 0 | 17.7 |
| DEX-MDZ-OP | 53.7 | 0 | 14.4 |
| DEX-MDZ-PF | 2.2 | 0 | 29.4 |
| DEX-PF | 65.1 | 0 | 11.1 |
| DEX-PF-OP | 49.4 | 0 | 15.7 |
| ESK-PF | 56.1 | 0 | 13.7 |
| ETM-MDZ-OP | 39.3 | 0 | 18.6 |
| KET-DEX | 26.4 | 0 | 22.4 |
| KET-DEX-MDZ | 3.3 | 0 | 29.0 |
| KET-DEX-MDZ-PF-OP | 71.3 | 0.9 | 9.3 |
| KET-ETM-MDZ | 49.8 | 1.3 | 15.6 |
| KET-MDZ-PF | 21.5 | 0 | 23.8 |
| KET-MDZ-PF-OP | 70.8 | 1.1 | 9.5 |
| KET-OP | 32.3 | 0 | 20.6 |
| KET-PF | 52.6 | 0 | 14.7 |
| KET-PF-OP | 99.7 | 94.5 | 1.1 |
| LIDO-MDZ-PF | 65.5 | 0.4 | 11.0 |
| LIDO-MDZ-PF-OP | 61.9 | 0 | 12.1 |
| LIDO-PF-OP | 57.1 | 0 | 13.4 |
| MDZ | 38.4 | 0 | 18.9 |
| MDZ-OP | 33.6 | 0 | 20.3 |
| MDZ-PF | 38.2 | 0 | 18.9 |
| MDZ-PF-OP | 31.4 | 0 | 20.9 |
| PF | 72.0 | 0 | 9.1 |
| RMZ | 42.8 | 0 | 17.6 |
| RMZ-OP | 70.9 | 0 | 9.4 |

**Table 23 Pairwise comparisons for recovery time**

| **Treatment/Comparison** | **Direct Coef.** | **Std. Err.** | **Indirect Coef.** | **Std. Err.** | **Difference Coef.** | **Std. Err.** | **p value** | **Tau** |
| --- | --- | --- | --- | --- | --- | --- | --- | --- |
| PF-OP vs RMZ-OP | – | – | – | – | – | – | – | – |
| CFL-OP vs PF-OP | – | – | – | – | – | – | – | – |
| DEX vs PF* | 0.677 | 0.707 | -0.702 | 63.265 | 1.379 | 63.268 | 0.983 | 0.693 |
| DEX-CFL-OP vs DEX-PF-OP* | -0.598 | 0.702 | 0.474 | 63.264 | -1.072 | 63.268 | 0.986 | 0.693 |
| DEX-MDZ vs KET-MDZ-PF* | 0.576 | 0.740 | 2.150 | 63.270 | -1.573 | 63.274 | 0.980 | 0.693 |
| DEX-MDZ-OP vs MDZ-OP | 0.056 | 0.371 | 2.255 | 0.801 | -2.199 | 0.883 | 0.013 | 0.614 |
| DEX-MDZ-OP vs MDZ-PF-OP | 1.960 | 0.726 | -0.239 | 0.503 | 2.199 | 0.883 | 0.013 | 0.614 |
| DEX-MDZ-PF vs KET-MDZ-PF* | -1.826 | 0.784 | -2.331 | 2.333 | 0.505 | 2.444 | 0.836 | 0.712 |
| DEX-MDZ-PF vs MDZ-PF* | -2.518 | 0.799 | -2.008 | 2.318 | -0.509 | 2.443 | 0.835 | 0.712 |
| DEX-PF vs PF-OP | -0.593 | 0.712 | 0.967 | 0.719 | -1.559 | 1.012 | 0.123 | 0.669 |
| DEX-PF vs MDZ-PF | 0.811 | 0.440 | -0.190 | 0.956 | 0.830 | 1.052 | 0.430 | 0.701 |
| DEX-PF vs PF | -0.425 | 0.757 | 0.016 | 0.645 | -0.442 | 0.994 | 0.657 | 0.709 |
| DEX-PF-OP vs PF-OP* | -0.237 | 0.522 | 0.299 | 31.630 | -0.536 | 31.635 | 0.986 | 0.693 |
| ESK-PF vs PF-OP | – | – | – | – | – | – | – | – |
| ETM-MDZ-OP vs MDZ-PF-OP* | 0.153 | 0.505 | 1.297 | 44.735 | -1.144 | 44.738 | 0.980 | 0.693 |
| KET-DEX vs PF-OP | – | – | – | – | – | – | – | – |
| KET-DEX-MDZ vs KET-ETM-MDZ* | -2.529 | 0.777 | -5.530 | 63.289 | 3.002 | 63.294 | 0.962 | 0.693 |
| KET-DEX-MDZ vs KET-MDZ-PF* | -1.691 | 0.738 | 0.279 | 28.302 | -1.970 | 28.312 | 0.945 | 0.693 |
| KET-DEX-MDZ-PF-OP vs KET-MDZ-PF-OP | – | – | – | – | – | – | – | – |
| KET-DEX-MDZ-PF-OP vs MDZ-PF* | 0.998 | 0.791 | 0.634 | 63.267 | 0.364 | 63.272 | 0.995 | 0.693 |
| KET-MDZ-PF vs MDZ-PF* | -0.692 | 0.770 | -0.438 | 0.948 | -0.254 | 1.222 | 0.835 | 0.712 |
| KET-MDZ-PF vs MDZ-PF-OP | -0.333 | 0.751 | -0.578 | 0.963 | 0.246 | 1.222 | 0.841 | 0.712 |
| KET-MDZ-PF-OP vs MDZ-PF* | 0.998 | 0.791 | 0.634 | 63.266 | 0.364 | 63.271 | 0.995 | 0.693 |
| KET-OP vs PF-OP | – | – | – | – | – | – | – | – |
| KET-PF vs PF-OP | – | – | – | – | – | – | – | – |
| KET-PF-OP vs PF-OP | – | – | – | – | – | – | – | – |
| LIDO-MDZ-PF vs MDZ-PF* | 0.803 | 0.731 | 0.967 | 63.267 | -0.164 | 63.271 | 0.998 | 0.693 |
| LIDO-MDZ-PF-OP vs MDZ-PF-OP* | 0.758 | 0.519 | 1.297 | 44.738 | -0.540 | 44.741 | 0.990 | 0.693 |
| LIDO-PF-OP vs PF-OP | – | – | – | – | – | – | – | – |
| MDZ vs MDZ-OP* | 0.014 | 0.722 | 1.216 | 63.262 | -1.202 | 63.266 | 0.985 | 0.693 |
| MDZ-OP vs PF-OP | -0.692 | 0.743 | -0.557 | 0.560 | -0.135 | 0.930 | 0.885 | 0.713 |
| MDZ-OP vs MDZ-PF-OP | -0.234 | 0.507 | 0.318 | 0.498 | -0.562 | 0.711 | 0.429 | 0.704 |
| MDZ-OP vs PF | -1.218 | 0.509 | -0.543 | 0.644 | -0.675 | 0.821 | 0.411 | 0.701 |
| MDZ-PF vs PF-OP | -0.195 | 0.747 | -0.734 | 0.694 | 0.538 | 1.020 | 0.598 | 0.708 |
| MDZ-PF vs PF | -0.383 | 0.722 | -1.153 | 0.605 | 0.770 | 0.942 | 0.414 | 0.701 |
| MDZ-PF-OP vs PF-OP | -0.453 | 0.520 | -0.940 | 0.634 | 0.487 | 0.820 | 0.553 | 0.707 |
| MDZ-PF-OP vs PF | -0.699 | 0.724 | -1.161 | 0.527 | 0.463 | 0.895 | 0.605 | 0.709 |
| PF vs RMZ* | 0.763 | 0.720 | 0.702 | 63.264 | 0.610 | 63.268 | 0.999 | 0.693 |
| **** Warning: all the evidence about these contrasts comes from the trials which directly compare them.*** | | | | | | | | |

**Table 24 Assessment of local heterogeneity for recovery time**

| **Loop** | | | | **IF** | **seIF** | **z_value** | **p_value** | **95% CI** | **Loop Heterogeneity (tau2)** |
| --- | --- | --- | --- | --- | --- | --- | --- | --- | --- |
| DEX-MDZ-OP | MDZ-OP | MDZ-PF-OP |  | 2.139 | 0.807 | 2.651 | 0.008 | (0.56, 3.72) | 0.148 |
| DEX-PF | MDZ-PF | PF-OP |  | 1.849 | 0.410 | 4.511 | 0.000 | (1.05, 2.65) | 0.000 |
| DEX-PF | MDZ-PF | PF |  | 0.876 | 1.760 | 0.498 | 0.619 | (0.00, 4.33) | 1.272 |
| DEX-PF | MDZ-OP | PF | PF-OP | 0.697 | 0.431 | 1.619 | 0.106 | (0.00, 1.54) | 0.000 |
| MDZ-OP | MDZ-PF | PF | PF-OP | 0.343 | 0.379 | 0.906 | 0.365 | (0.00, 1.09) | 0.000 |
| MDZ-OP | MDZ-PF-OP | PF |  | 0.279 | 0.210 | 1.332 | 0.183 | (0.00, 0.69) | 0.000 |
| DEX-PF | MDZ-PF-OP | PF | PF-OP | 0.270 | 0.411 | 0.657 | 0.511 | (0.00, 1.07) | 0.000 |
| MDZ-PF | MDZ-PF-OP | PF | PF-OP | 0.084 | 0.356 | 0.237 | 0.813 | (0.00, 0.78) | 0.000 |
| KET-MDZ-PF | MDZ-PF | MDZ-PF-OP | PF | 0.043 | 0.442 | 0.098 | 0.922 | (0.00, 0.91) | 0.000 |
| KET-MDZ-PF | MDZ-PF | MDZ-PF-OP | PF-OP | 0.041 | 0.470 | 0.088 | 0.930 | (0.00, 0.96) | 0.000 |
| MDZ-OP | MDZ-PF-OP | PF-OP |  | 0.034 | 0.654 | 0.051 | 0.959 | (0.00, 1.32) | 0.120 |
| ****** Note: Loop (DEX-MDZ-PF, KET-MDZ-PF, MDZ-PF) is formed only by multi-arm trial(s) - Consistent by definition***  ****** Note: Loop (KET-DEX-MDZ-PF-OP, KET-MDZ-PF-OP, MDZ-PF) is formed only by multi-arm trial(s) - Consistent by definition*** | | | | | | | | | |

**Figure 14 Network Plot for recovery time**

*
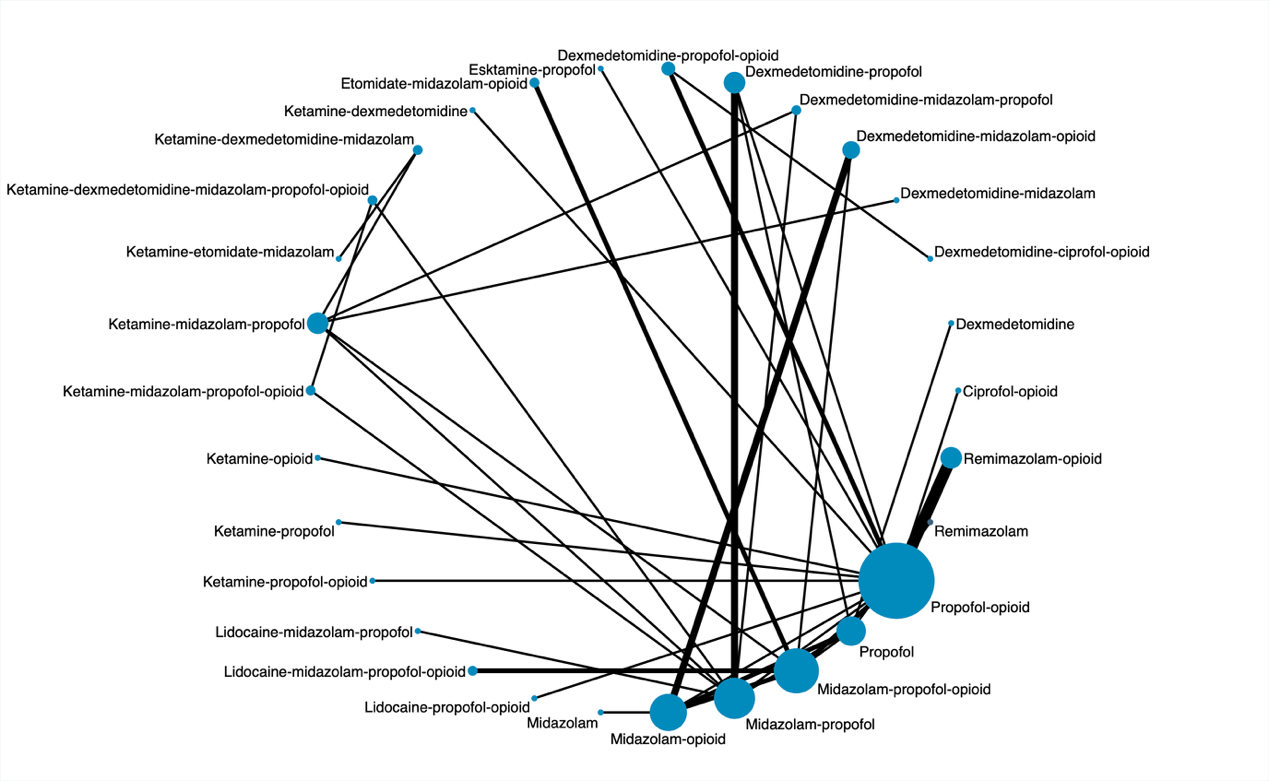
*

**Figure 15 Cumulative ranking curves for recovery time**

**
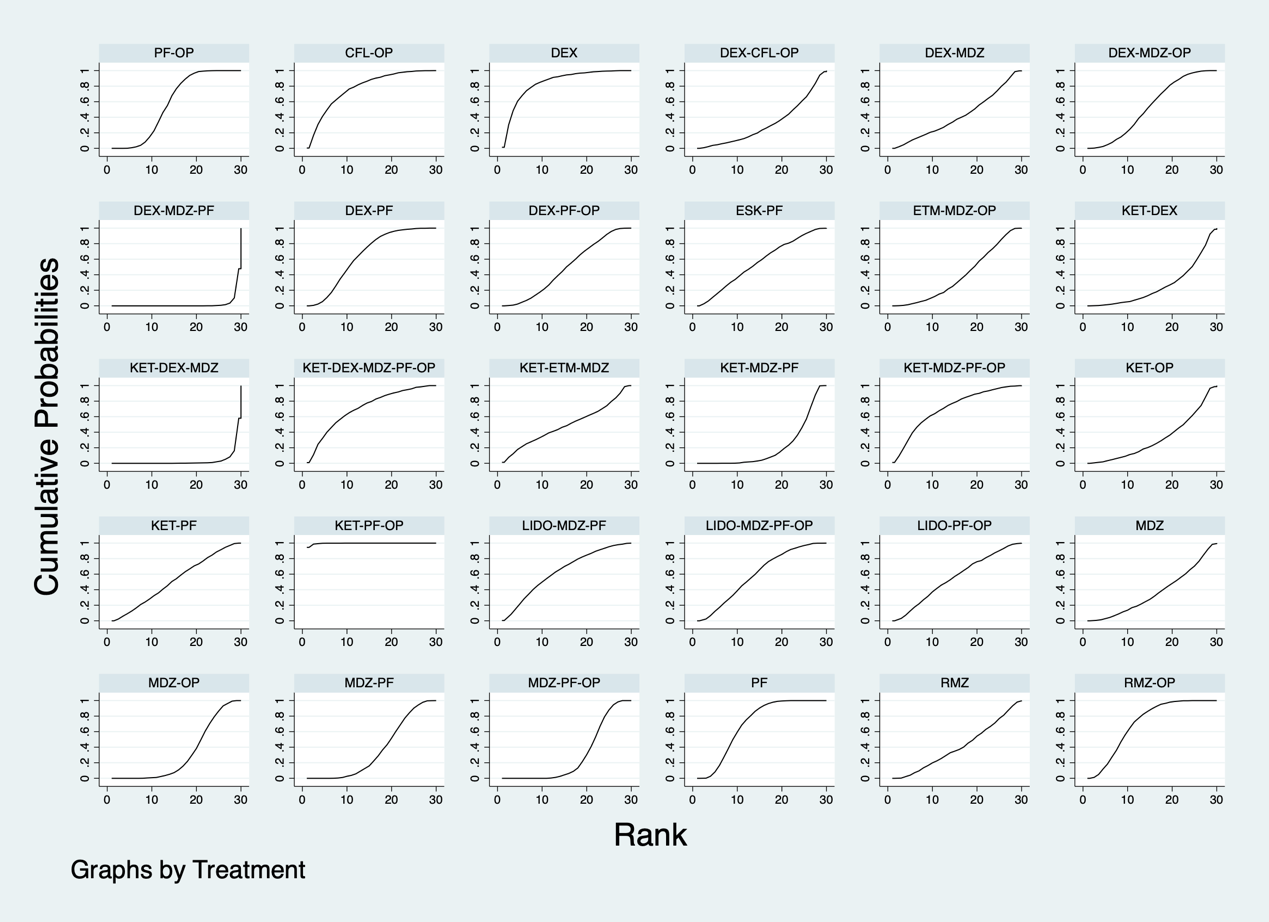
**

**Table 25 Meta-regression Analyses of Prespecified Covariates for Outcomes with Substantial Heterogeneity**

| **Outcomes** | **Covariates** | | | | | | | |
| --- | --- | --- | --- | --- | --- | --- | --- | --- |
|  | **type of endoscopic procedure**  **(ERCP vs. ESD vs. Other types)** | | **mean age**  **(years)** | | **procedural time**  **(min)** | | **Risk of Bias**  **(Low vs. unclear or high)** | |
|  | *t*-value | *P*-value | *t*-value | *P*-value | *t*-value | *P*-value | *t*-value | *P*-value |
| Procedural interference events | 1.000 | 0.327 | -0.150 | 0.880 | 0.780 | 0.442 | 0.620 | 0.542 |
| Hypotension | -0.010 | 0.992 | 0.420 | 0.678 | 1.480 | 0.145 | -0.110 | 0.914 |
| Bradycardia | -0.220 | 0.826 | 1.380 | 0.176 | 1.290 | 0.207 | -1.950 | 0.059 |
| Recovery Time | 0.270 | 0.788 | -0.470 | 0.640 | -1.040 | 0.302 | 0.080 | 0.940 |
